# Impact of Ligand Design on an Iron NHC Epoxidation Catalyst

**DOI:** 10.1002/open.202400071

**Published:** 2024-09-24

**Authors:** Tim P. Schlachta, Greta G. Zámbó, Michael J. Sauer, Isabelle Rüter, Fritz E. Kühn

**Affiliations:** ^1^ Technical University of Munich School of Natural Sciences Department of Chemistry and Catalysis Research Center, Molecular Catalysis Lichtenbergstraße 4 85748 Garching Germany; ^2^ Institut für Anorganische Chemie Georg-August-Universität Göttingen Tammannstraße 4 37077 Göttingen Germany

**Keywords:** Non-heme iron complexes, Carbene ligands, Epoxidation, Iron NHC complexes, Tailoring catalytic properties

## Abstract

An open‐chain iron pyridine‐NHC framework is expanded utilizing a benzimidazole moiety to deepen the understanding of the impact of electronic variations on iron NHC epoxidation catalysts, especially regarding the stability. The thereby newly obtained iron(II) NHC complex is characterized and employed in olefin epoxidation. It is remarkably temperature tolerant and achieves a TOF of ca. 10 000 h^−1^ and TON of ca. 700 at 60 °C in the presence of the Lewis acid Sc(OTf)_3_, displaying equal stability, but lower activity than the unmodified iron pyridine‐NHC (pre‐)catalyst. In addition, a synthetic approach towards another ligand containing 2‐imidazoline units is described but formylation as well as hydrolysis hamper its successful synthesis.

## Graphical abstract

An open‐chain iron pyridine‐NHC framework is expanded utilizing a benzimidazole moiety. The new iron(II) NHC complex is characterized and employed in olefin epoxidation. It is remarkably temperature tolerant and achieves a TOF of ca. 10 000 h^−1^ and TON of ca. 700 at 60 °C with Sc(OTf)_3_ as additive.

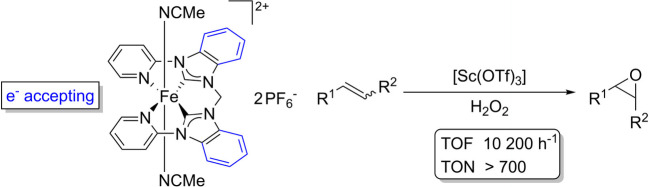

## 1 Introduction

**Figure 1:**
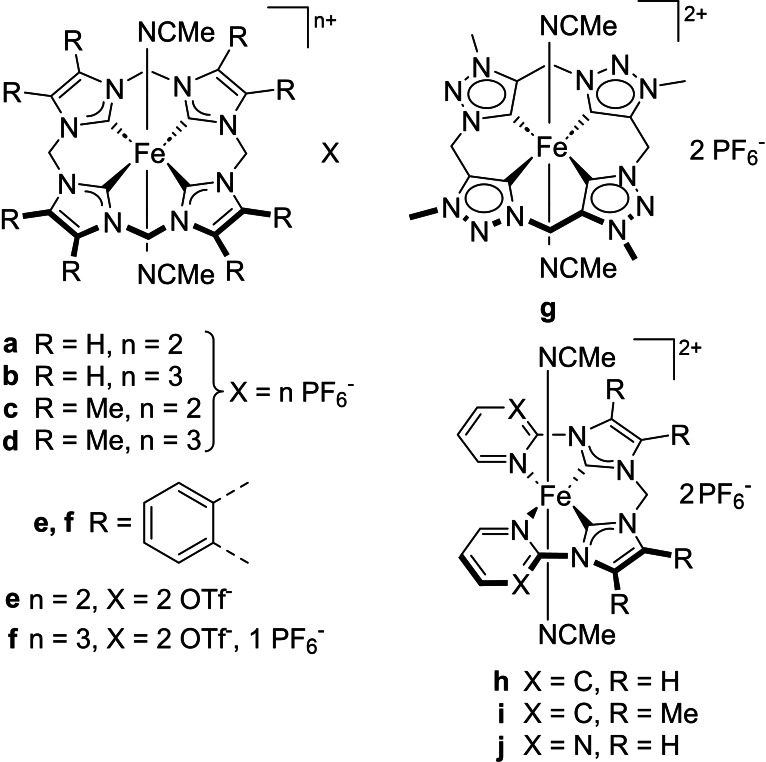
Iron NHC (pre‐)catalysts.

Epoxidation of olefins is an important process to produce commodity chemicals, fine chemicals, pharmaceuticals as well as building blocks for synthetic organic chemistry.[^1^, ^2^, ^3^, ^4^] The majority of homogeneous epoxidation catalysts are transition metal complexes based on, for example, Re, Ru, W, polyoxometalates, Pt or Mo.^[2]^ In a bio‐inspired approach, iron‐containing enzymes like cytochrome P450 have been used as models for a number of Fe epoxidation catalysts.^[5]^ Originally to study the chemistry of these metalloproteins, the ability of the enzymes to oxidize challenging substrates at mild conditions with high activity and selectivity, has made the artificial iron complexes interesting candidates for catalytic application.[^6^, ^7^] Similar to the models in nature, first generations of biomimetic Fe catalysts had *N*‐donor ligands such as porphyrins or non‐heme ligands.[^6^, ^8^, ^9^, ^10^, ^11^, ^12^, ^13^, ^14^] While the use of iron makes their application financially attractive, their activity given in turnover frequency (TOF, up to 25 200 h^−1^)^[15]^ lacks somewhat behind other organometallic benchmark catalysts containing Re (up to ca. 40 000 h^−1^)^[16]^ and Mo (up to ca. 50 000 h^−1^).^[17]^ In this context, iron *N*‐heterocyclic carbene (NHC) complexes have shown to be on par or better to *N*‐ligated iron complexes, especially in terms of activity.[^16^, ^18^, ^19^, ^20^, ^21^] The two most studied catalytic systems possess a cyclic iron tetracarbene and open‐chain iron pyridine‐NHC framework (Figure 1).[^22^, ^23^, ^24^, ^25^] The iron tetracarbene complex **a**/**b** is the current benchmark system for homogeneous olefin epoxidation with a TOF >400 000 h^‐1^ and turnover number (TON) of around 1 200 at room temperature in combination with the Lewis acid Sc(OTf)_3_ as additive.^[19]^ The open‐chain iron NHC complex **h** is still on par with the most active *N*‐ligated iron catalyst with a TOF of 24 500 h^−1^ and a TON of >700, also in the presence of Sc(OTf)_3_ at room temperature.^[23]^ While the stability (in TON) of both systems is also better compared to *N*‐ligated iron catalysts, where most TONs are below 100,[^12^, ^15^, ^23^, ^26^, ^27^] it still has to be improved for potential industrial applications. By suppression of a degradation pathway with Lewis acids like Sc(OTf)_3_, the formation of a diiron(III)‐*μ*‐oxo complex, the TON could already be more than doubled from ca. 500 to ca. 1 200 for **a** and similarly for **h**, **i** and **j**.[^18^, ^19^, ^23^, ^28^, ^29^] As another strategic option, the modification of the axial ligands has been chosen, as it appeared to be the synthetically simplest approach to modify the system.^[22]^ Interestingly, it was possible in this way to increase the stability of **h** in C−H hydroxylation by up to 34 %.^[30]^ However, at least one of the labile axial ligands apparently needs to leave the molecule to enable catalysis by creating a free coordination site.[^5^, ^31^, ^32^] Accordingly, an exchange of both axial ligands to stronger coordinating ones has a negative effect on the catalytic performance, at least in the case of the well examined olefin epoxidation.[^33^, ^34^, ^35^]

A more electron rich iron center is assumed to facilitate the proposed rate determining step in epoxidation, the formation of an electrophilic iron(IV)‐oxo or iron(V)‐oxo intermediate.[^22^, ^23^, ^36^, ^37^] On the other hand, a more Lewis acidic iron center might increase the reactivity of the electrophilic active species. Thus, equatorial ligand modifications toward a higher (**c**/**d**, **g**, **i**) and lower (**e**/**f**, **j**) electron density at the iron center were executed. However, so far, the changes did neither lead to a higher stability nor to higher activity. Both catalytic systems (**a**/**b** and **h**) remain most active and stable when unmodified; a more electron rich iron center (**c**/**d**, **g**, **i**) leads to a lower activity and decreased stability while a less electron donating ligand (**e**/**f**, **j**) results in an even lower activity and additionally to a slightly lower stability, compared to the unmodified complex.[^20^, ^23^, ^37^]

**Figure 2:**
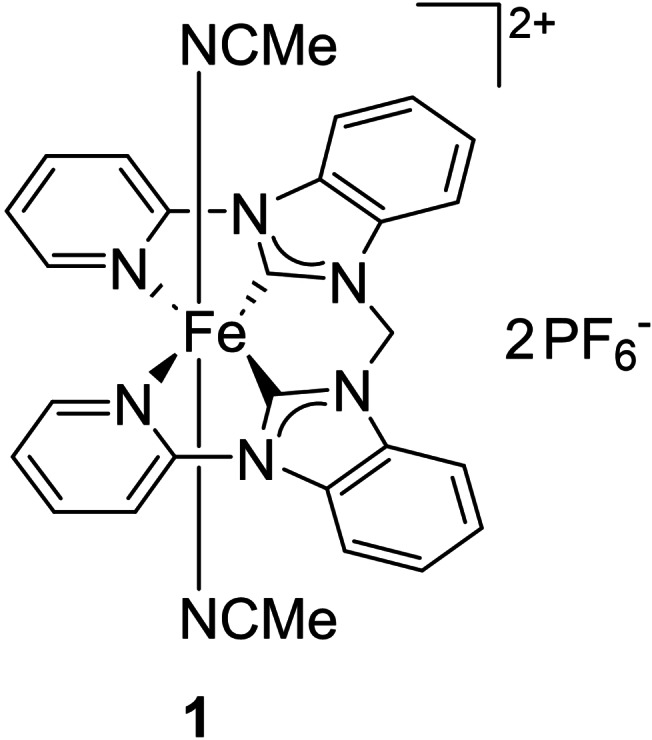
Novel iron(II) NHC complex.

In this work, the open‐chain iron pyridine‐NHC framework is expanded with a benzimidazole moiety (**1**, Figure 2) and examined in order to deepen the understanding of the impact of electronic variations on iron NHC epoxidation catalysts, especially regarding stability. This modification is analog to **e**/**f**, allowing to investigate whether it has a similar effect on the open‐chain catalytic system. Furthermore, a synthetic approach toward a ligand precursor containing 2‐imidazoline units is described. The previous ligand modifications all had a measurable influence on the catalytic performance. Changing the NHC backbone from unsaturated to saturated might be a desirable, sufficiently small change with a gentle electron donation toward the iron center. As there is an increasing interest in the application of machine learning models in chemistry, the publication of training data is needed, especially of otherwise neglected unsuccessful reactions.^[38]^ The successful synthesis and in‐depth characterization of the novel iron(II) NHC complex **1** is reported. **1** is employed as (pre‐)catalyst in the epoxidation of *cis*‐cyclooctene as model substrate. Additionally, more challenging olefin substrates and the effect of Lewis acid Sc(OTf)_3_ is investigated. The results are discussed within the framework of the current research state on iron NHC catalyzed olefin epoxidations. The catalytic activity of **1** is assumed to be lower compared to the other complexes. A slower ongoing reaction and degradation would possibly allow the investigation of the epoxidation mechanism of iron NHCs by e. g. trapping intermediates or real‐time monitoring at low temperatures. Elucidating the mechanism would also help to find suitable strategies to improve stability.

## 2 Results and Discussion

### 2.1 Synthesis and Characterization of the Iron(II) Complex 1

The synthesis of a ligand precursor, analogous to **h**, but containing a saturated backbone, *i. e*. 2‐imidazoline moieties instead of imidazole, was pursued parallel to the synthesis of iron(II) NHC complex **1**. However, formylation as well as hydrolysis posed potential problems during the synthesis. The synthetic approaches are described in the SI.

**Scheme 1:**
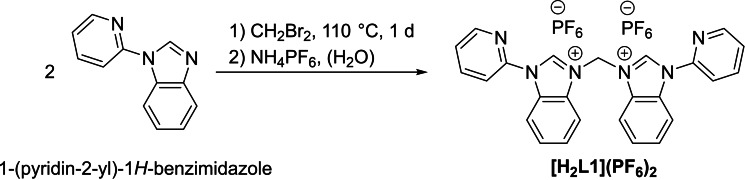
Synthesis of ligand precursor **[H_2_L1](PF_6_)_2_**.

The ligand precursor of iron(II) complex **1** is obtained by a two‐step synthesis (Scheme 1). First, 1‐(pyridin‐2‐yl)‐1*H*‐benzimidazole is reacted with excess CH_2_Br_2_ to give the bromide salt **[H_2_L1](Br)_2_**. Then, an anion exchange with NH_4_PF_6_ is performed to increase the solubility of the compound in organic solvents as well as additional purification step to yield **[H_2_L1](PF_6_)_2_**.

**Scheme 2:**
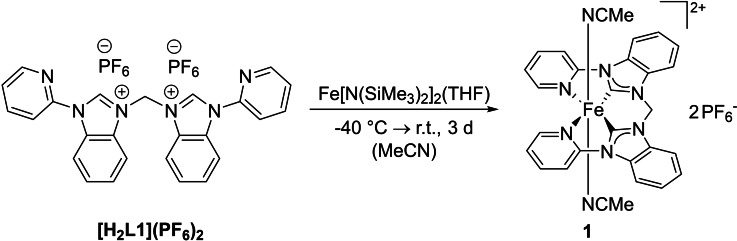
Synthesis of iron(II) complex **1**.

The synthesis of iron(II) NHC complex **1** proved to be rather challenging. An established effective way to synthesize Fe NHC complexes is the use of Fe[N(SiMe_3_)_2_]_2_ as both internal base and iron precursor and has been used for the similar complexes **h**, **i** and **j**.[^22^, ^23^] However, in this case, the conversion remains very low, leaving a large amount of unreacted ligand precursor and an inorganic impurity (see SI). The iron complex and the ligand precursor are difficult to separate, most likely due to very similar polarity, with the iron complex being slightly more polar. While at this point **1** could be isolated analytically pure by precipitation with a specific ratio of Et_2_O to MeCN, the yield was very low (3 %, method A), and the purification method was poorly reproducible. A more complete reaction would simplify the purification. Thus, efforts were made to increase the conversion of the ligand precursor. Experiments with iron precursor (FeBr_2_(THF)_2_, Fe(OAc)_2_) in combination of a (stronger) external base (NaH, KN(SiMe_3_)_2_, *n*‐BuLi, LiN^*i*^Pr_2_) were conducted (see SI), even though the proton at position 2 of the benzimidazole units should be more acidic than for the imidazole ligand precursors of e. g. **h** due to the conjugative effect of the benzene ring.^[39]^ In addition, the transmetalation route was applied with Ag_2_O and FeBr_2_(THF)_2_.^[40]^ However, these approaches were not successful. Finally, the best method to obtain **1** (8 % yield, method B) proved to be the reaction of **[H_2_L1](PF_6_)_2_** with Fe[N(SiMe_3_)_2_]_2_ (Scheme 2) and removal of unreacted ligand precursor afterwards by multiple washing steps with a mixture of MeCN/Et_2_O with increasing polarity (see SI). Then, the inorganic impurity derived from unreacted iron precursor can be removed by filtration over neutral Al_2_O_3_ (see SI).

The successful synthesis of **1** is evidenced in ^1^H NMR by the absence of the acidic benzimidazole protons in position 2 and the appearance of the carbene carbon signal in ^13^C NMR at 230.31 ppm. The ^13^C_NHC_ signal of **1** is significantly downfield shifted compared to the other NCCN‐ligated complexes (**h**: 216.15, **i**: 215.49, **j**: 216.24 ppm)[^23^, ^41^] and the effect is also observed in the iron tetracarbene **e**/**f**.^[37]^ This shift can be explained with the lower electron density at the carbene carbon atom compared to the other complexes **h**, **i**, **j** and the followed deshielding noticeable in NMR. The anellation of the benzene ring to the backbone of the NHC results in a transfer of the π‐electron density from the carbene carbon into the larger conjugated π system.[^42^, ^43^] Other factors may also contribute.^[22]^ The chemical composition of **1** is further shown by ESI‐MS and elemental analysis. Similar to **h**, **i** and **j**, **1** is stable as solid under ambient conditions for at least one month and in MeCN solution (HPLC‐grade, not dried or degassed) for at least one day.^[23]^

In cyclic voltammetry, the reversible Fe^2+^/Fe^3+^ transition of **1** is located at *E*_1/2_=0.625 V (▵E=91 mV, V *vs*. Fc/Fc^+^, see SI) and is the most positive in the series of the NCCN complexes (**h**: 0.423 V, **i**: 0.337 V, **j**: 0.559 V).[^23^, ^41^, ^44^, ^45^, ^46^] The strong downfield shift of the ^13^C_NHC_ signal and this positive redox potential reflect the weaker σ electron donation of the benzimidazolylidene ligand to the iron center, supported by Tolman electronic parameters,[^43^, ^47^] and implies a lower electron density at the iron atom.^[22]^

The UV/Vis spectrum of **1** is similar to the other NCCN‐ligated complexes.^[23]^ It has four local absorption maxima (227 nm, 279 nm, see SI) and the two at 327 nm and 393 nm are assigned to charge‐transfer bands.^[45]^

**Figure 3:**
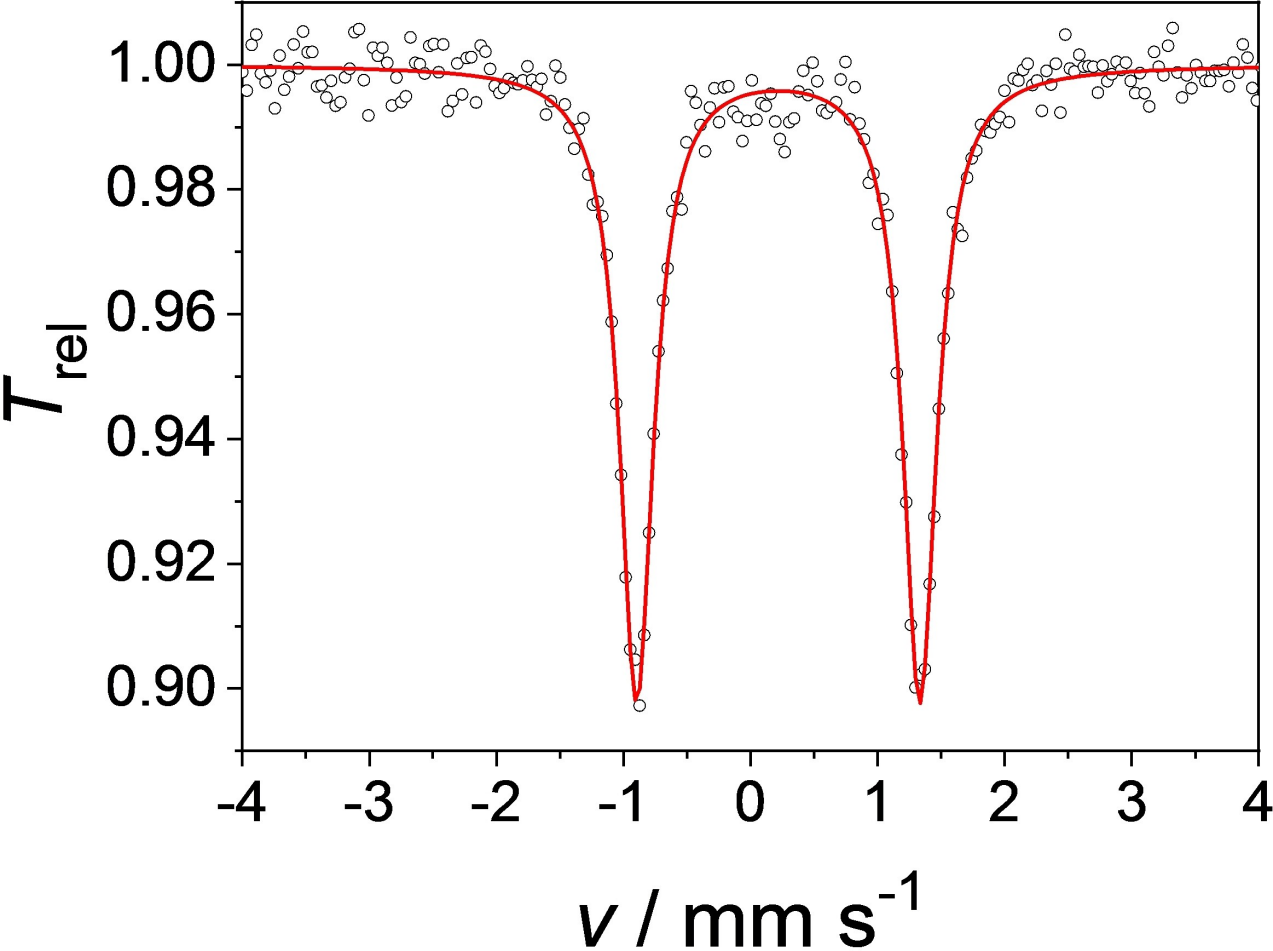
Zero‐field ^57^Fe Mössbauer spectrum of solid **1** at 80 K. The red line represents a simulation with *δ*=0.22 mm s^−1^ and ▵*E*_Q_=2.23 mm s^−1^.

A solid sample of **1** was characterized by ^57^Fe Mössbauer spectroscopy to gain more insights on the electronic properties of the complex (Figure 3). As indicated in ^1^H NMR, **1** is a diamagnetic iron(II) complex with an isomer shift of *δ*=0.22 mm s^−1^ in the range of other octahedral iron(II) low‐spin species.[^22^, ^23^, ^48^] The isomer shift is close to the other three Fe NCCN complexes (▵*δ* up to 0.04 mm s^−1^)^[23]^ as they all exhibit similar Fe‐ligand bond lengths (see below) and no significant influence of the benzimidazolylidene ligand is noticeable. For example, a shorter bond length can increase the electron density at the iron atom through compression of the s‐orbitals, resulting, among other factors, in a more negative isomer shift.^[48]^ The quadrupole splitting of **1** is relatively high for an iron(II) low‐spin complex with ▵*E*_Q_=2.23 mm s^−1^, but still in the typical range for NHC‐ligated iron(II) low‐spin complexes.[^22^, ^23^] This is due to the NHC ligand's strong σ‐donation in the equatorial plane deforming the charge distribution around the iron nucleus. The quadrupole splitting of the NCCN‐ligated complexes could be ranked in the order of increasing ligand electron donor strength, **j**<**h**<**i**, resulting in a higher amount of deformation of the electric field.^[23]^ However, **1** has a quadrupole splitting of ▵*E*_Q_=2.23 mm s^−1^, effectively the same as **i** (▵*E*_Q_=2.22 mm s^−1^), which is counterintuitive at first glance, since **1** exhibits the weakest electron donation from the NHC ligand. The high quadrupole splitting can probably be attributed to the stronger π‐accepting properties of the benzimidazolylidene ligand.^[43]^

**Figure 4:**
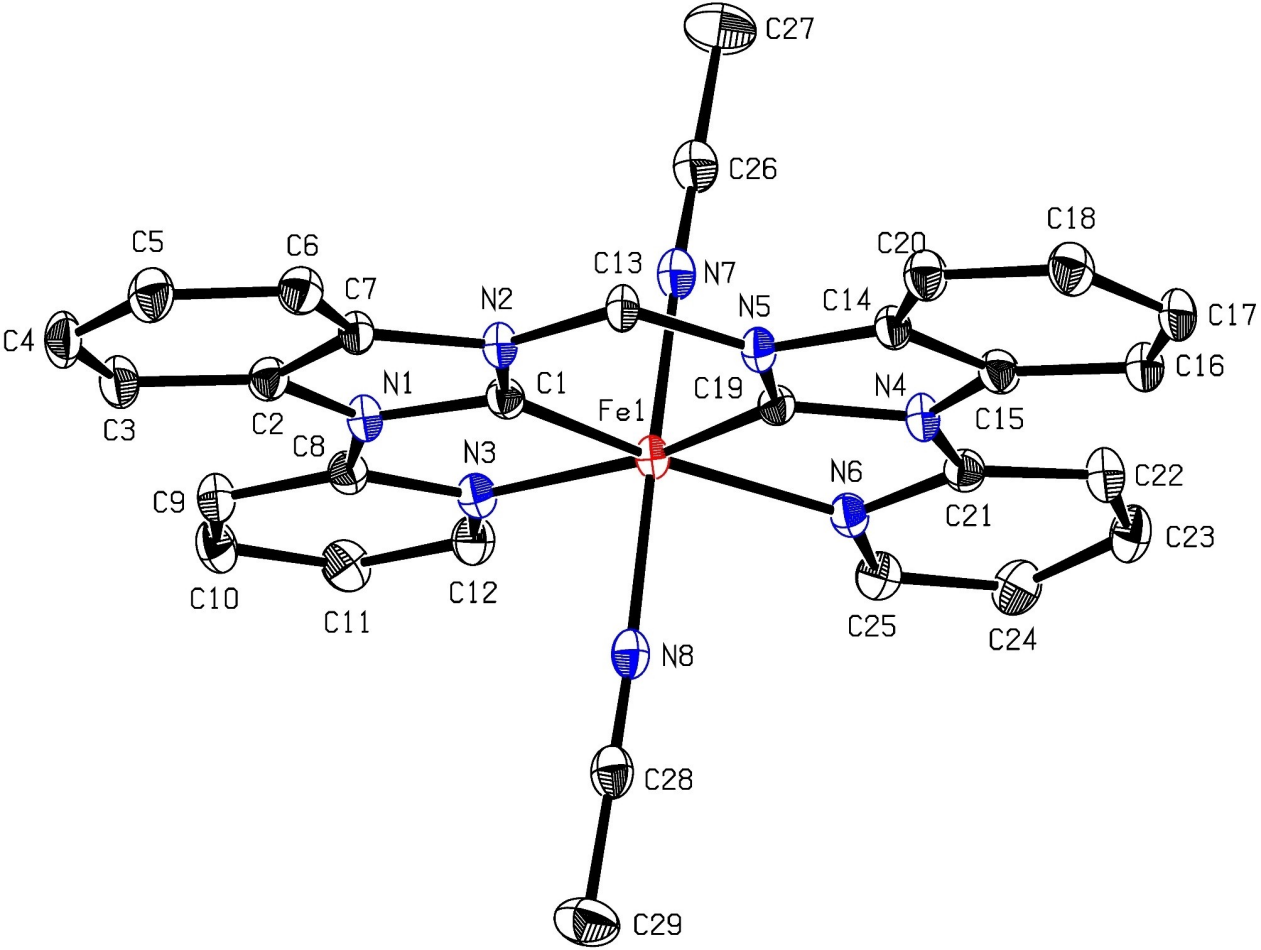
ORTEP‐style representation of **1**. Hydrogen atoms and hexafluorophosphate anions are omitted for clarity. Thermal ellipsoids are shown at a 50 % probability level. Selected bond lengths (Å) and angles (°): C1−Fe1 1.8314(19); N3−Fe1 2.0721(16); C19−Fe1 1.8293(19); N6−Fe1 2.0852(16); N7−Fe1 1.9136(18); N8−Fe1 1.9253(18); N7−Fe1−N8 171.34(7); C1−Fe1−N3 79.90(7); C1−Fe1−N6 166.66(8); C1−Fe1−N7 95.12(8); C1−Fe1−N8 91.09(8).

Single crystals suitable for X‐ray diffraction were obtained by slow vapor diffusion of Et_2_O into a solution of **1** in MeCN (see SI). The molecular structure of **1** is displayed in Figure 4 and it is similar to the other complexes **h**, **i**, **j**.^[23]^ The NHC ligand is coordinating in equatorial fashion and the labile MeCN ligands are located at the axial coordination sites of the iron center. The axis intersecting the axial ligands and the iron atom is slightly bent (171.34°) like in **h**, **i**, **j**, while it is usually closer to 180° in iron tetracarbenes.^[22]^ The other parameters are in the typical range of open‐chain iron complexes.^[23]^ The buried volume of **1** is 86 %V_Bur_ (see SI) and is therefore the same size as **h**, **i**, **j**, ensuring a good comparability in catalysis due to equal steric properties of the iron(II) (pre‐)catalysts.

### 2.2 Catalytic Olefin Epoxidation Reactions

The influence of the backbone modification on the catalytic performance of complex **1** in the epoxidation of olefins is investigated in the following. For a good comparability of the results with **h**, **i** and **j**, identical conditions are applied if not stated otherwise. Hence, standard conditions are defined as 20 °C, applying H_2_O_2_ (1.50 eq.) as oxidant, MeCN as solvent, *cis*‐cyclooctene (1.00 eq.) as model substrate and **1** as (pre‐)catalyst (0.02 eq.).^[23]^ Excess hydrogen peroxide was used as it has been found to be best option for iron NHC epoxidation catalysis and is atom‐efficient as well as environmentally friendly.[^18^, ^23^, ^49^, ^50^, ^51^] Quantification of the formed epoxide was done with GC‐FID and ^1^H NMR spectroscopy.

**Figure 5:**
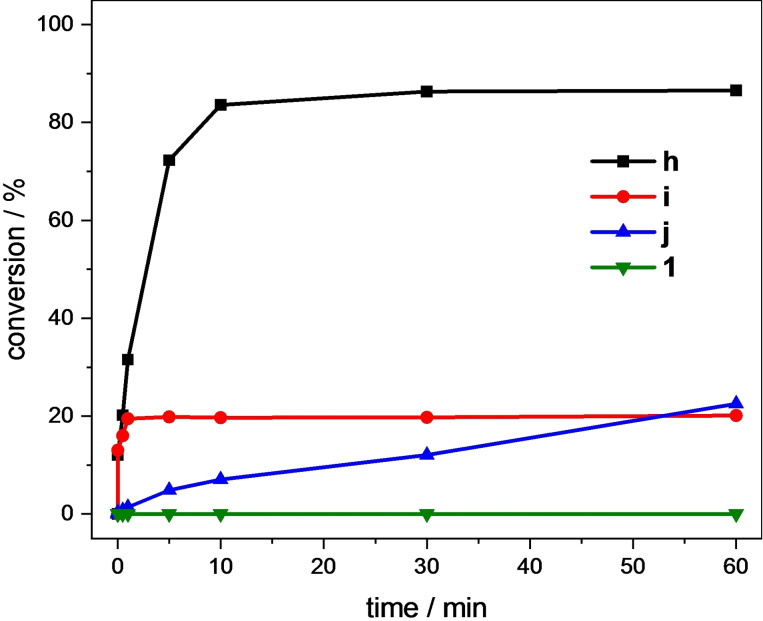
Time‐dependent epoxidation of *cis*‐cyclooctene (67.3 μmol/mL, 1.00 eq.) in MeCN using **h**, **i**, **j** ^[23]^ or **1** (1.35 μmol/mL, 0.02 eq.) as catalyst and H_2_O_2_ (50 % aq., 101 μmol/mL, 1.50 eq.) as oxidizing agent at 20 °C. Conversions are determined by GC‐FID.

A first experiment was conducted under standard conditions. With the weaker electron donating NHC ligand and the highest redox potential, **1** was expected to show the lowest activity out of **h**, **i** and **j**. However, over the course of 60 min, no conversion was detected at all (Figure 5). A behavior like **j** was anticipated, of course with slightly lower conversion. The performance of **1** is likely a result of a low activity but can also imply a fast degradation within the first seconds. For **h**, de‐coordination of the NCCN ligand and subsequent reaction to a C−C coupled biimidazole species was found as one degradation pathway under oxidative conditions.[^52^, ^53^]

**Figure 6:**
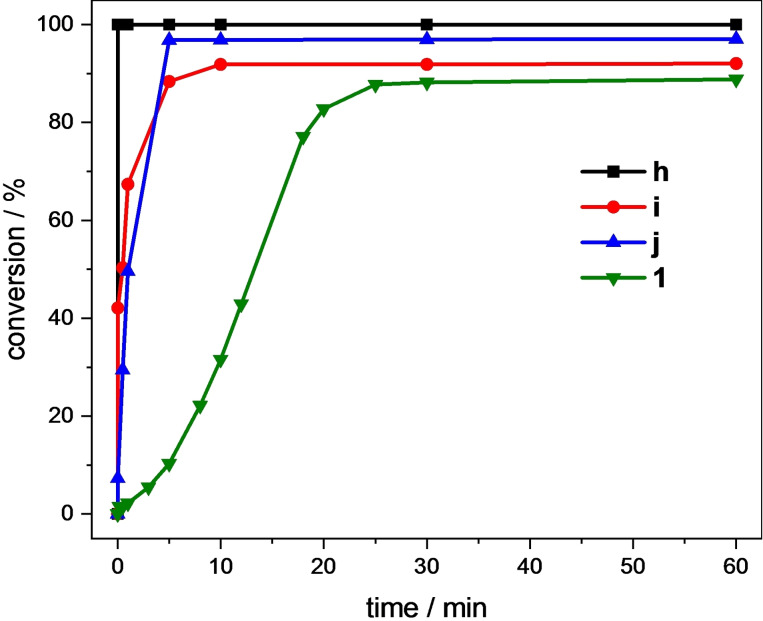
Time‐dependent epoxidation of *cis*‐cyclooctene (67.3 μmol/mL, 1.00 eq.) in MeCN using **h**, **i**, **j** ^[23]^ or **1** (1.35 μmol/mL, 0.02 eq.) as catalyst, Sc(OTf)_3_ (6.73 μmol/mL, 0.10 eq.) and H_2_O_2_ (50 % aq., 101 μmol/mL, 1.50 eq.) as oxidizing agent at 20 °C. Conversions are determined by GC‐FID.

The addition of Lewis acids to the reaction has shown to significantly increase the activity and stability of the iron NHC catalyst.[^19^, ^22^, ^33^] One reason for this is the reactivation of a potential dead‐end species by the Lewis acid, an diiron(III)‐*μ*‐oxo complex formed during catalysis of **a**/**b**.[^19^, ^28^, ^29^] Furthermore, the Lewis acid is facilitating the initial oxidation of Fe^II^ to Fe^III^ as well as OH⋅/^−^ cleavage of a proposed Fe^III^−OOH intermediate transferring the rate determining step to the olefin oxidation.[^19^, ^22^, ^23^, ^54^] Hence, the same experiment was repeated with Sc(OTf)_3_ as additive, which has proven to be the most effective Lewis acid for **a**/**b**.^[19]^ In its presence **1** reaches a conversion of 89 % (Figure 6), which is close to the other complexes (**h**: 100 %; **i**: 92 %; **j**: 97 %)^[23]^ and indicative for a deactivation pathway which now appears to be suppressed. At these conditions, the selectivity of **1** (99 %) is on par with the other NCCN complexes and **a**/**b**, being among the most selective iron NHC epoxidation catalysts.[^18^, ^19^, ^23^] Noticeable is the induction period of **1**, which can be attributed to preoxidation from Fe^II^ to Fe^III^, aided by Sc(OTf)_3_ this time, and is also observed for iron(II) tetracarbenes, e. g. **a** or **e**.[^18^, ^37^] This reinforces the proposed mechanism, which continues with the iron(III) complex to form a Fe^III^−OOH intermediate, followed by the presumably rate‐determining step, the homolytic or heterolytic cleavage to generate iron(IV) or iron(V)‐oxo species, respectively. Finally, the epoxide is obtained after electrophilic attack of the oxo species on the alkene.^[22]^ As **1** takes more than 30 min to reach its final conversion, it has the lowest TOF (300 h^−1^, Table 1 entry 2) of the four NCCN complexes.^[23]^ However, the TON is on the same level (**1**: 44; **h**: 50; **i**: 46; **j**: 49).^[23]^

**Table 1:** Epoxidation of *cis*‐cyclooctene by **1** at different catalyst concentrations, temperature, and with or without additive.

entry	catalyst	T [°C]	loading [mol %]	additive	X [%]^[a]^ (60 min)	S [%] (60 min)	TOF [h^−1^]^[b]^	TON (60 min)	
1	**1**	20	2	–	0	0	0	0	
2	**1**	20	2	Sc(OTf)_3_	89	99	300	44	
3	**1**	40	2	–	11	>99	–	5	^[c]^
4	**1**	40	2	Sc(OTf)_3_	90	91	1 000	45	
5	**1**	60	2	Sc(OTf)_3_	87	80	5 200	43	
6	**1**	60	0.5	Sc(OTf)_3_	82	91	8 500	164	^[d]^
7	**1**	60	0.1	Sc(OTf)_3_	71	92	10 200	706	^[e]^

Reaction conditions: *cis*‐cyclooctene (67.3 μmol/mL, 1.00 eq.) in MeCN, Fe catalyst, if stated Sc(OTf)_3_ (6.73 μmol/mL, 0.10 eq.), and H_2_O_2_ (50 % aq., 101 μmol/mL, 1.50 eq.). Selectivity is related to the epoxide. [a] Conversions are determined by GC‐FID. [b] TOFs are determined at the highest slope of X. [c] X, S and TON determined after 300 min. [d] X, S and TON determined after 10 min. [e] X, S and TON determined after 5 min. T=temperature. X=conversion. S=selectivity.

**Figure 7:**
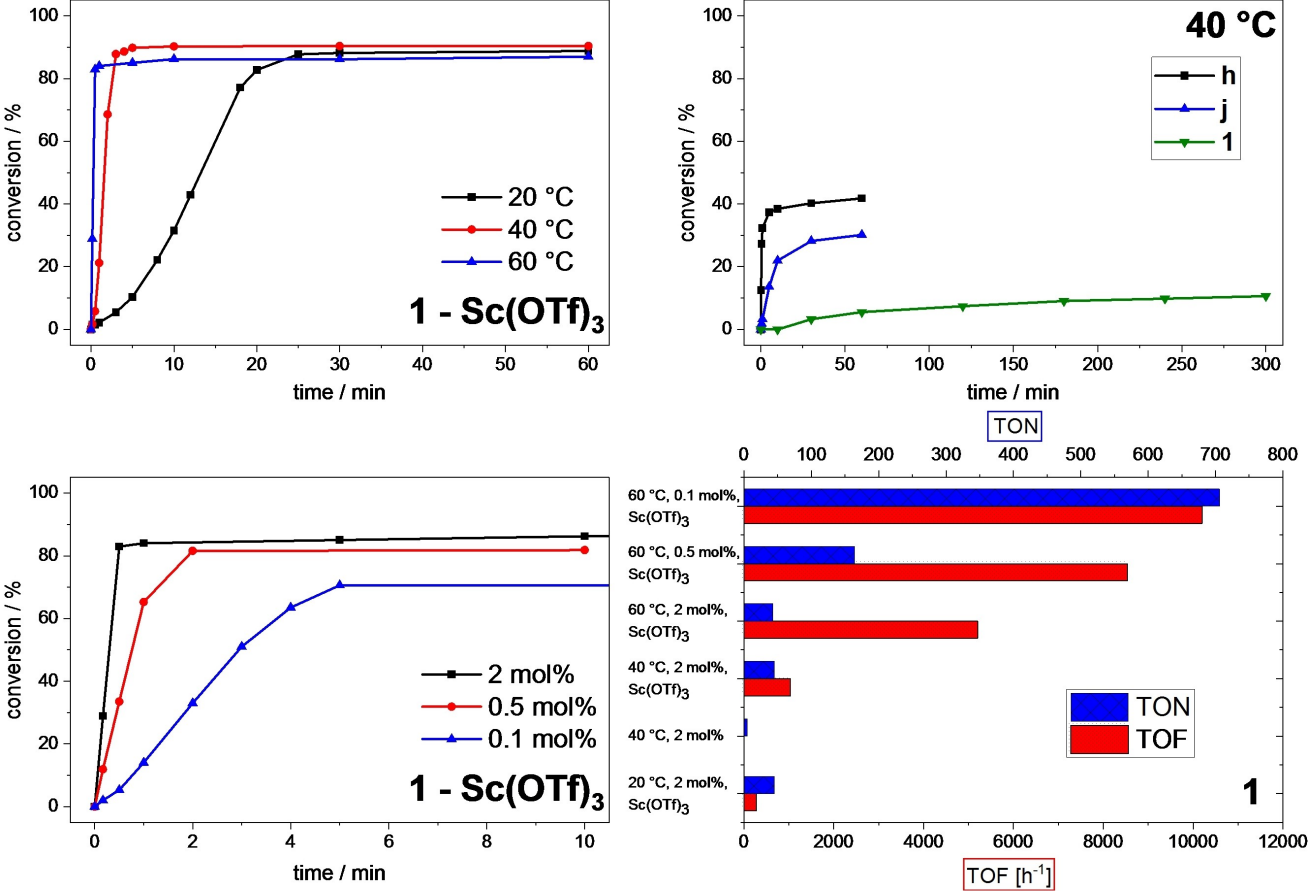
Time‐dependent epoxidation of *cis*‐cyclooctene (67.3 μmol/mL, 1.00 eq.) in MeCN using Fe catalyst and H_2_O_2_ (50 % aq., 101 μmol/mL, 1.50 eq.) as oxidizing agent. Conversions are determined by GC‐FID. Top left: **1** (1.35 μmol/mL, 0.02 eq.) as catalyst and Sc(OTf)_3_ (6.73 μmol/mL, 0.10 eq.) at 20, 40 and 60 °C. Top right: **h**, **j** ^[23]^ or **1** (1.35 μmol/mL, 0.02 eq.) as catalyst at 40 °C. Bottom left: **1** (0.02 eq., 0.005 eq. or 0.001 eq.) as catalyst and Sc(OTf)_3_ (6.73 μmol/mL, 0.10 eq.) at 60 °C. Bottom right: overview of TON and TOF of **1** at different conditions.

Both **j** and **e**/**f** are remarkably temperature tolerant and achieve a higher activity at higher temperatures.[^23^, ^37^] Therefore, **1** was screened at different temperatures at otherwise standard conditions (Figure 7). Indeed, in the presence of Sc(OTf)_3_ at 40 °C, a final conversion of 90 % is already reached after 5 min corresponding to a TOF of over 1 000 h^−1^ (entry 4). At 60 °C, most of the conversion is reached even faster after 30 s (83 %, Figure 7), equal to a TOF of 5 200 h^−1^ (entry 5). The TON effectively does not change at higher temperatures (40 °C: 45; 60 °C: 43), contrary to **j** and **e**/**f** showing declining TONs but similarly increasing activity like **1**. However, the selectivity is lower in these experiments (40 °C: 91 %; 60 °C: 80 %). Striking though is that the selectivity is >99 % in the beginning of both runs and gradually decreases over time, while the amount of formed cyclooctane‐1,2‐diol is increasing (see SI). This strongly suggests catalytic ring‐opening of the epoxide by the Lewis acid Sc(OTf)_3_ as described in the literature.[^55^, ^56^, ^57^, ^58^, ^59^] Another evidence is the high selectivity (>99 %, entry 3) achieved with **1** at 40 °C without Sc(OTf)_3_ after even 300 min. This experiment also demonstrates the long lifetime of **1** under oxidative conditions, although only a conversion of 11 % is reached in the end (**h**: 42 %; **j**: 30 %, Figure 7).

At lower catalyst concentrations at 60 °C an induction period can be observed again despite the use of Sc(OTf)_3_ (Figure 7). At these seemingly optimal conditions, the highest TOF with 10 200 h^−1^ and TON with 706 are found for **1** (entry 7). Regarding the activity, this places **1** second behind **h**, with the order **h** (24 500 h^−1^)>**1** (10 200 h^−1^)>**i** (7 600 h^−1^)>**j** (1 300 h^−1^), while having the most positive redox potential and weakest NHC ligand.^[23]^ However, at identical conditions, for example 20 °C with Sc(OTf)_3_, **1** possesses as expected the lowest activity (**h**: 18 000 h^−1^>**i**: 7 600 h^−1^>**j**: 1 300 h^−1^>**1**: 300 h^−1^).^[23]^ The stability of **1** in TON is effectively as high as **h**, resulting in the order **h** (711)≈**1** (706)>**j** (49)>**i** (46).^[23]^ While the TOF of **1** is lower than the most active *N*‐ligated Fe complex (25 200 h^−1^),^[15]^ its TON is like **h** on par with the highest obtained for a *N*‐donor Fe complex (715),^[27]^ which usually have TONs around 200 or below.[^12^, ^15^, ^23^, ^26^] The iron tetracarbene **a**/**b** still has the best reported catalytic performance (TOF>400 000 h^−1^; TON=1 200 at r. t.).^[19]^

A selection of other substrates was examined with **1** at standard conditions in the presence of Sc(OTf)_3_ (Table 2). They are important building blocks in industry^[23]^ and contain functional groups that allow the performance of **1** to be studied for more sophisticated alkenes. The reaction time is set to 30 min based on the achieved full conversion in the case of *cis*‐cyclooctene (Figure 6). Although the reaction time was only 5 min for **h**, **i**, **j**,^[23]^ this should allow to determine the actual performance of **1** for the different substrates while considering its lower activity. Thus, a good comparability amongst the NCCN complexes should be ensured, as the reaction time is standardized relative to the completeness of the reaction with *cis*‐cyclooctene.

**Table 2:** Epoxidation of various olefin substrates using **1** as catalyst with 30 min reaction time and Sc(OTf)_3_.

entry	substrate	X [%]	S [%]	
8	*cis*‐cyclooctene	88	>99	^[a]^
9	cyclohexene	29	4	
10	1‐hexene	22	43	
11	*cis*‐2‐octene	37	18	
12	*trans*‐2‐octene	54	56	
13	allyl alcohol	36	29	
14	allyl chloride	10	>99	
15	styrene	16	26	
16	chalcone	8	26	

Reaction conditions: substrate (67.3 μmol/mL, 1.00 eq.) in MeCN, **1** (1.35 μmol/mL, 0.02 eq.), Sc(OTf)_3_ (6.73 μmol/mL, 0.10 eq.) and H_2_O_2_ (50 % aq., 101 μmol/mL, 1.50 eq.), 20 °C, 30 min. Selectivity is related to the epoxide. Conversions are determined by ^1^H‐NMR spectroscopy, applying benzene as external standard. [a] Conversion determined by GC‐FID. X=conversion. S=selectivity.

Overall, **1** exhibits a moderate performance when compared to the other NCCN complexes. While the conversion of cyclohexene is approximately as high as for **i** and **j**,^[23]^ the selectivity is surprisingly low for **1** (4 %, entry 8). The conversion of 1‐hexene appears to be challenging for **1** reaching only 22 % with a selectivity of 43 % (entry 10). Together with **i** and **j**, **1** favors *trans*‐2‐octene (30 % yield) over the *cis* isomer (7 % yield), contrary to **h**, **b** and other iron epoxidation catalysts.[^18^, ^23^, ^60^] The backbone modifications indicate to have an opposing influence in this regard, possibly because of steric reasons. For all the three linear carbon chains, significant diol formation is measured (1‐hexene: 6 %; *trans*‐2‐octene: 16 %; *cis*‐2‐octene: 19 % yield). The conversion of allyl alcohol and allyl chloride is lower, but the selectivity is on par or better than for the NCCN complexes. No epoxide formation could be observed with **h**, **i**, **j** for styrene,^[23]^ but despite severe aldehyde production (12 % yield, see SI), 4 % yield of product was achieved. In contrast, even though the conversion of chalcone was low for **i** and **j**,^[23]^ **1** only manages to consume 8 % (entry 16). To summarize, for the various substrates, the nucleophilicity of the alkene is decisive for its reactivity, underlining the electrophilic nature of the active species. This is clearly demonstrated for chalcone, for example: The −M effect of the carbonyl group reduces the nucleophilicity and thereby reactivity of the carbon‐carbon double bond, resulting in the observed low conversion.

## 3 Conclusions and Outlook

A synthetic approach towards a NCCN ligand containing 2‐imidazoline units is described. Formylation as well as hydrolysis pose potential problems. A novel iron(II) NHC complex **1** containing a NCCN ligand with benzimidazole moieties has been successfully synthesized by two different methods. **1** contains the most positive redox potential amongst the iron NCCN complexes described so far.^[23]^ In epoxidation of *cis*‐cyclooctene, this results in a noticeable induction phase – oxidation from Fe^II^ to Fe^III^ – even in the presence of Sc(OTf)_3_ at 20 °C. Higher temperatures and the Lewis acid Sc(OTf)_3_ are beneficial for the catalytic performance of **1** and the catalyst species is – compared to other iron NHC epoxidation catalysts[^22^, ^23^] – remarkably temperature tolerant (up to 60 °C). Regardless of its high redox potential induced by the weaker σ‐donation from the NHC ligand, **1** achieves the second highest TOF of 10 200 h^−1^ amongst the other iron NCCN complexes at 60 °C. Furthermore, a TON of 706 is determined, as high as observed for the unmodified iron NCCN complex **h**. In contrast to the benzimidazole modification in the iron tetracarbene system **e**/**f**, **1** can keep the stability of its unmodified derivative, **h**. When screening various other substrates, **1** also favors the more nucleophilic alkenes, similar to **h**, **i** and **j**. However, due to its low reactivity at 20 °C, **1** achieves only modest conversions. Based on its slow reaction, the suitability to use **1** for mechanistic studies is demonstrated, particularly low temperatures should ensure controlled conditions.

## Experimental

### General Procedures and Analytical Methods

The synthesis of complex **1** was performed in argon atmosphere using standard Schlenk and glovebox techniques as well as dry and degassed solvents. *N* ^1^‐(Pyridin‐2‐yl)ethane‐1,2‐diamine,^[61]^ 1‐(pyridin‐2‐yl)‐1*H*‐benzimidazole^[62]^ and Fe[N(SiMe_3_)_2_]_2_(THF)^[23]^ were synthesized according to literature procedures. The procedures for novel compounds obtained during the synthetic approaches to the saturated ligand precursor, containing 2‐imidazoline moieties instead of imidazole, (2‐imidazoline, 2‐((2‐aminoethyl)amino)pyridine 1‐oxide, 2‐(2‐imidazolin‐1‐yl)pyridine 1‐oxide) are stated in the SI. 1‐(Pyridin‐2‐yl)‐1*H*‐benzimidazole was purified by column chromatography on silica gel (~30 g silica per gram of crude product) using ethyl acetate as eluent (R_f_=0.29) prior to use. Solvents were purified, dried and degassed using standard methods^[63]^ or received from a solvent purification system by M. Braun. All other chemicals were obtained from commercial suppliers and were used without further purification. NMR spectra were recorded on a Bruker Avance Ultrashield AV400 (^1^H NMR, 400.13 MHz; ^13^C NMR, 100.53 MHz). The chemical shifts are given in *δ* values in ppm (parts per million) relative to TMS (tetramethylsilane) and are reported relative to the residual deuterated solvent signal.^[64]^ Elemental analyses (C/H/N/S) were obtained by the microanalytical laboratory at Technical University Munich. Electrospray ionization mass spectrometry (ESI‐MS) data were measured on a Thermo Fisher Ultimate 3000. Electrochemical measurements were carried out in a scintillation vial closed with a septum lid under argon atmosphere, equipped with a glassy carbon disc electrode (working electrode), a platinum wire electrode (counter electrode) and a silver wire (pseudo reference electrode) on a Metrohm Autolab PGSTAT302N potentiostat. Potentials are measured with a scan rate of 100 mV/s and are reported with reference to an internal standard of ferrocenium/ferrocene (Fc+/0; 0.8 mg). Tetrabutylammonium hexafluorophosphate (100 mM in MeCN) was used as electrolyte. The concentration of the iron complex was about 2 mM. The UV/Vis spectrum was recorded on an Agilent Cary 60 UV–Vis spectrophotometer with a concentration of 0.02 mM complex in acetonitrile. Solid material of **1** (30 to 40 mg) was studied using ^57^Fe Mössbauer spectroscopy at 80 K. The ^57^Fe Mössbauer spectrum was measured using a ^57^Co source in a Rh matrix using an alternating constant acceleration Wissel Mößbauer spectrometer equipped with a Janis closed‐cycle helium cryostat. Transmission data were collected, and isomer shifts are reported relative to iron metal at ambient temperature. Experimental data were simulated with *mf2.SL* software.^[65]^ Deposition Number 2326822 (for **1**) contain the supplementary crystallographic data for this paper. These data are provided free of charge by the joint Cambridge Crystallographic Data Centre and Fachinformationszentrum Karlsruhe Access Structures service.

### Catalytic Procedures

**Experimental remarks**. GC analysis was performed with an Agilent Technologies 7890B GC‐FID system with a 7693A Automatic Liquid Sampler for 150 samples with G4513A Autoinjector using a HP‐5 column (30 m×320 μm×0.25 μm). NMR spectra were recorded on a Bruker Avance Ultrashield AV400 (400 MHz) or AV500 (500 MHz) spectrometer at a temperature of 297 K. Chemical shifts (*δ*) are reported in ppm and referenced to the residual signal of the deuterated solvent.^[64]^

**Catalytic procedure**. All catalytic reactions were conducted in a cryostat (JulaboFP‐50). Acetonitrile (HPLC‐grade) as solvent was applied for all experiments, which are screened *via* GC (substrate: *cis*‐cyclooctene). The screening of other substrates (*cis*‐cyclohexene, 1‐hexene, allyl alcohol, allyl chloride, styrene, chalcone, *cis*‐2‐octene and *trans*‐2‐octene) was performed using ^1^H NMR spectroscopy and deuterated acetonitrile as solvent. The catalyst was added from a preformed stock solution in acetonitrile corresponding to the appropriate stoichiometry to a solution of the respective substrate (1.00 eq., 67.3 μmol/mL). Hydrogen peroxide (50 % aq., 1.50 eq., 101 μmol/mL) was used as oxidizing agent and, if required, Sc(OTf)_3_ as additive (0.10 eq., 8.41 μmol/mL). The reaction was started upon addition of the catalyst stock solution, by adding the catalyst solution all at once. The reaction was terminated by adding electrolytically precipitated activated MnO_2_ in order to decompose the excess of H_2_O_2_ in the reaction solution. After filtration over activated neutral alumina (separation of the catalyst), GC samples were prepared for each experiment and time point using 200 μL filtrate, diluted with 1300 μL MeCN, in which *p*‐xylene (0.9 μL/mL) is dissolved as an external standard. For the screening *via* ^1^H NMR spectroscopy, 500 μL filtrate was added to 1 μL benzene as external standard. Control experiments without catalyst were performed for all reactions and did not show catalytic activity. An additional blank experiment with a simple iron salt, iron(II) chloride, in the presence of H_2_O_2_ was conducted to highlight the importance of iron complexes associated with NHCs due to minimal product and unselective side‐product formation. Analogous, the additive Sc(OTf)_3_ itself shows minimal unselective catalytic activity.[^19^, ^37^, ^55^]

### Synthetic Procedures

**[H_2_L1](Br)_2_**. 1‐(Pyridin‐2‐yl)‐1*H*‐benzimidazole (266 mg, 1.36 mmol, 2.00 eq.) is dissolved in excess dibromomethane (10 mL, 143 mmol, 105 eq.) and heated to 110 °C for 1 d. The white suspension is cooled to r. t., then 10 mL Et_2_O is added. The precipitate is filtrated, washed with 5 mL MeCN and 10 mL Et_2_O to obtain [H_2_L1](Br)_2_ as white powder (249 mg, 441 μmol, 65 %). ^1^H NMR (400.13 MHz, DMSO‐*d_6_*): δ 11.13 (s, 2H, NC*H*N), 8.82 (dd, ^3^ *J*=5.2 Hz, ^4^ *J*=1.7 Hz, 2H, *H_ar_*), 8.61 (d, ^3^ *J*=8.3 Hz, 2H, *H_ar_*), 8.48 (d, ^3^ *J*=8.3 Hz, 2H, *H_ar_*), 8.35 (td, ^3^ *J*=7.8 Hz, ^4^ *J*=1.9 Hz, 2H, *H_ar_*), 8.12 (d, ^3^ *J*=8.3 Hz, 2H, *H_ar_*), 7.91 (td, ^3^ *J*=7.8 Hz, ^4^ *J*=1.1 Hz, 2H, *H_ar_*), 7.81 (m, 4H, *H_ar_*), 7.67 (s, 2H, C*H_2_*). MS‐ESI (m/z): [HL1]^+^ calcd., 403.17; found, 403.18 (6); [L1 – 1‐(pyridin‐2‐yl)‐1*H*‐benzimidazole+MeOH] calcd., 240.11; found, 240.07 (100); [1‐(pyridin‐2‐yl)‐1*H*‐benzimidazole+H^+^] calcd., 196.09; found, 196.12 (68).

**[H_2_L1](PF_6_)_2_**. [H_2_L1](Br)_2_ (196 mg, 347 μmol, 1.00 eq.) is dissolved in 10 mL H_2_O and slowly added to a vigorously stirred solution of NH_4_PF_6_ (410 mg, 2.52 mmol, 7.24 eq.) in 15 mL H_2_O. After stirring for 30 min, the white precipitate is filtered off and washed with H_2_O and Et_2_O to obtain [H_2_L1](PF_6_)_2_ as somewhat hygroscopic white powder (180 mg, 259 μmol, 75 %). The product is immediately transferred into a Schlenk flask, dried overnight at 70 °C at 10^−3^ mbar and stored under argon. ^1^H NMR (400.13 MHz, CD_3_CN): δ 9.91 (s, 2H, NC*H*N), 8.76 (dd, ^3^ *J*=5.0 Hz, ^4^ *J*=1.8 Hz, 2H, *H_ar_*), 8.36 (m, 2H, *H_ar_*), 8.22 (td, ^3^ *J*=7.9 Hz, ^4^ *J*=1.8 Hz, 4H, *H_ar_*), 7.88 (m, 6H, *H_ar_*), 7.73 (dd, ^3^ *J*=7.6 Hz, ^3^ *J*=4.9 Hz, 2H, *H_ar_*), 7.28 (s, 2H, C*H_2_*). ^13^C NMR (100.53 MHz, CD_3_CN): δ 151.16 (*C_ar_*), 147.41 (*C_ar_*), 142.90 (*C_ar_*), 141.80 (*C_ar_*), 132.08 (*C_ar_*), 131.26 (*C_ar_*), 130.03 (*C_ar_*), 129.96 (*C_ar_*), 127.31 (*C_ar_*), 118.88 (*C_ar_*), 117.22 (*C_ar_*), 114.54 (*C_ar_*), 57.26 (*C*H_2_). MS‐ESI (m/z): [L1+PF_6_^−^]^+^ calcd., 549.14; found, 548.65 (20); [HL1]^+^ calcd., 403.17; found, 403.16 (66); [1‐(pyridin‐2‐yl)‐1*H*‐benzimidazole+H^+^] calcd., 196.09; found, 196.03 (100). Anal. calcd. for C_25_H_20_F_12_N_6_P_2_: C 43.24; H 2.90; N 12.10. Found: C 43.26; H 2.94; N 12.06.

**[FeL1(MeCN)_2_](PF_6_)_2_ (1). Method A**. A −40 °C cold solution of Fe[N(SiMe_3_)_2_]_2_(THF) (108 mg, 241 μmol, 1.05 eq.) in 4 mL MeCN is added to a −40 °C cold solution of [H_2_L1](PF_6_)_2_ (159 mg, 229 μmol, 1.00 eq.) in 4 mL MeCN. The solution becomes red and is stirred at 95 °C for 2 d. The dark red solution is dried under vacuum. The black residue is dissolved in MeCN and dried again under vacuum to remove any remaining free amine HN(SiMe_3_)_2_. The crude is dissolved in 2 mL MeCN and 5.4 mL Et_2_O is added to precipitate a first fraction of a brown precipitate. The supernatant is filtered off and the resulting light brown to yellow solid is dried under vacuum, to obtain the analytically pure iron complex **1** (ca. 6 mg, 7 μmol, 3 %). At this point, tiny washing steps with MeCN can be performed (around 0.3 to 0.5 mL MeCN), if necessary.

**Method B**. A black −40 °C cold solution of Fe[N(SiMe_3_)_2_]_2_(THF) (2.91 g, 6.49 mmol, 1.11 eq.) in 10 mL MeCN is added to a yellow −40 °C cold solution of [H_2_L1](PF_6_)_2_ (4.08 g, 5.87 mmol, 1.00 eq.) in 15 mL MeCN. The solution becomes red at first but turns black upon complete addition and is stirred at r. t. for 3 d. A brown precipitate has formed at the bottom of the black solution. The following work‐up is performed under ambient atmosphere without the use of Schlenk technique. The black reaction mixture is dried *in vacuo* resulting in a black residue. A series of washing steps is performed to remove unreacted [H_2_L1](PF_6_)_2_ and other organic impurities from the residue: 12 mL MeCN (obtained from solvent purification system, *i. e*. somewhat dry) is added to the residue in order to completely suspend the solid without any remaining precipitate. 60 mL Et_2_O (neither degassed nor dried) is added to the black mixture and the dark yellow supernatant is decanted through a filter in order to catch any solid particles and is discarded. All black solids are combined to be suspended in MeCN again. This process is repeated further 9 times, *i. e*. in total 10 washing steps with the combination of 12 mL MeCN and 60 mL Et_2_O. Then, 3 steps with 12 mL MeCN and 55 mL Et_2_O are performed, followed by 3 steps with 12 mL MeCN and 50 mL Et_2_O. Finally, 9 steps with 14 mL MeCN and 40 mL Et_2_O are conducted. As the purification progresses, the mixture becomes lighter resulting in a light brown solid and yellow filtrate in the end. The light brown solid is dissolved in 100 mL MeCN. The resulting black solution is filtrated over a plug of Al_2_O_3_ (37 g Al_2_O_3_; pH=7; 7 cm height; column diameter 3 cm; dry Al_2_O_3_ and not wetted beforehand) to remove inorganic impurities. After filtration of the 100 mL solution, the plug of Al_2_O_3_ is not washed with additional MeCN as this can re‐dissolve the impurities. An intense orange solution is collected and the solvent is removed *in vacuo*. The resulting orange solid is re‐dissolved in 18 mL MeCN and 100 mL Et_2_O is added to form a yellow precipitate and orange supernatant. The mixture is cooled at −32 °C for 10 min. Afterwards, the suspension is filtrated and the yellow precipitate washed with Et_2_O. The precipitate is dried overnight at 60 °C at 10^−2^ mbar and stored under argon. [FeL1(MeCN)_2_](PF_6_)_2_ can be obtained as yellow solid in 8 % yield (406 mg, 489 μmol). Single crystals suitable for X‐ray diffraction were obtained by slow vapor diffusion of Et_2_O into a solution of [FeL1(MeCN)_2_](PF_6_)_2_ in MeCN after 1 to 2 weeks at r. t. under ambient atmosphere (see SI for details). ^1^H NMR (400.13 MHz, CD_3_CN): δ 9.69 (m, 2H, *H_ar_*), 8.50 (m, 4H, *H_ar_*), 8.33 (m, 2H, *H_ar_*), 8.03 (m, 2H, *H_ar_*), 7.86 (td, ^3^ *J*=5.6 Hz, ^4^ *J*=2.7 Hz, 2H, *H_ar_*), 7.69 (m, 4H, *H_ar_*), 7.45 (s, 2H, C*H_2_*), 1.96 (s, 6H, C*H_3_* CN). ^13^C NMR (100.53 MHz, CD_3_CN): δ 230.31 (*C_carbene_*), 155.95 (*C_ar_*), 153.47 (*C_ar_*), 142.75 (*C_ar_*), 138.05 (*C_ar_*), 133.95 (*C_ar_*), 125.83 (*C_ar_*), 125.61 (*C_ar_*), 124.07 (*C_ar_*), 114.37 (*C_ar_*), 112.72 (*C_ar_*), 110.80 (*C_ar_*), 62.60 (*C*H_2_). MS‐ESI (m/z): [FeL1+HCOO^−^+HCOONa]^+^ calcd., 571.08; found, 570.92 (19); [FeL1+HCOO^−^]^+^ calcd., 503.09; found, 502.87 (74); [FeL1(MeCN)_2_]^2+^ calcd., 270.07; found, 269.76 (85); [FeL1(MeCN)]^2+^ calcd., 249.56; found, 249.51 (45); [FeL1]^2+^ calcd., 229.04; found, 229.32 (100). Anal. calcd. for C_29_H_24_F_12_FeN_8_P_2_: C 41.95; H 2.91; N 13.50. Found: C 41.61; H 2.98; N 13.97.

## Conflict of Interests

There are no competing interests to declare.

4

## Supporting information

As a service to our authors and readers, this journal provides supporting information supplied by the authors. Such materials are peer reviewed and may be re‐organized for online delivery, but are not copy‐edited or typeset. Technical support issues arising from supporting information (other than missing files) should be addressed to the authors.

Supporting Information

## Data Availability

The data that support the findings of this study are available in the supplementary material of this article.
