# Supplementary material for: Impact of Ligand Design on an Iron NHC Epoxidation Catalyst
Source: ChemistryOpen. 2024 Sep 24;13(12):e202400071. doi: 10.1002/open.202400071 (PMC11625922; doi:10.1002/open.202400071)
Supplement: Supplementary file 1 — Supporting Information [file OPEN-13-e202400071-s001.pdf]

ChemistryOpen

Supporting Information

Impact of Ligand Design on an Iron NHC Epoxidation Catalyst

Tim P. Schlachta, Greta G. Zámbó, Michael J. Sauer, Isabelle Rüter, and Fritz E. Kühn*

Supporting Information (SI)

Impact of Ligand Design on an Iron NHC Epoxidation Catalyst

Tim P. Schlachta[1], Greta G. Zámbó[1], Michael J. Sauer[1], Isabelle Rüter[2], Fritz E. Kühn[1,*]

[1]Technical University of Munich, School of Natural Sciences, Department of Chemistry and Catalysis Research Center, Molecular Catalysis, Lichtenbergstraße 4, 85748 Garching, Germany. E-mail: fritz.kuehn@ch.tum.de; Phone (secretary's office): +49 (0)89 289 13477

[2]Institut für Anorganische Chemie, Georg-August-Universität Göttingen, Tammannstraße 4, 37077 Göttingen, Germany.

[*] Corresponding Author

Table of contents

1. NMR spectroscopy 2
2. ESI-MS spectra....................6
3. Crystallographic data 8
4. Cyclic voltammetry....................12
5. UV/Vis spectroscopy....................15
6. Buried volume and topographic steric map calculations....................16
7. Catalytic olefin epoxidation reactions of *cis*-cyclooctene....................19
8. ^{1}H NMR spectra of catalytic substrate screening....................22
9. Additional analytical data on the synthesis of iron(II) complex 1....................29
10. Synthetic attempts: saturated ligand precursor....................37
11. References SI 52

1. NMR spectroscopy

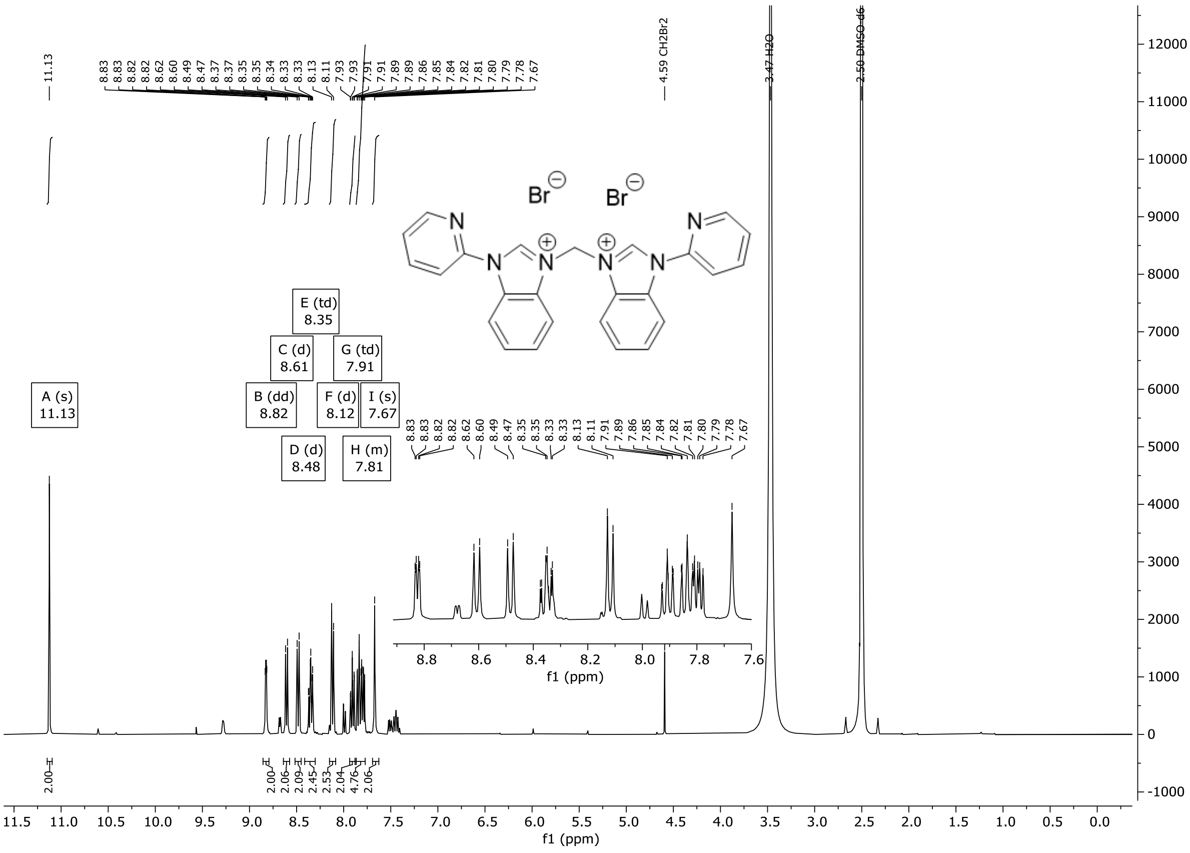

Figure 1 SI. ^{1}H NMR spectrum of $[H_2L1](Br)_2$ in DMSO-d_6.

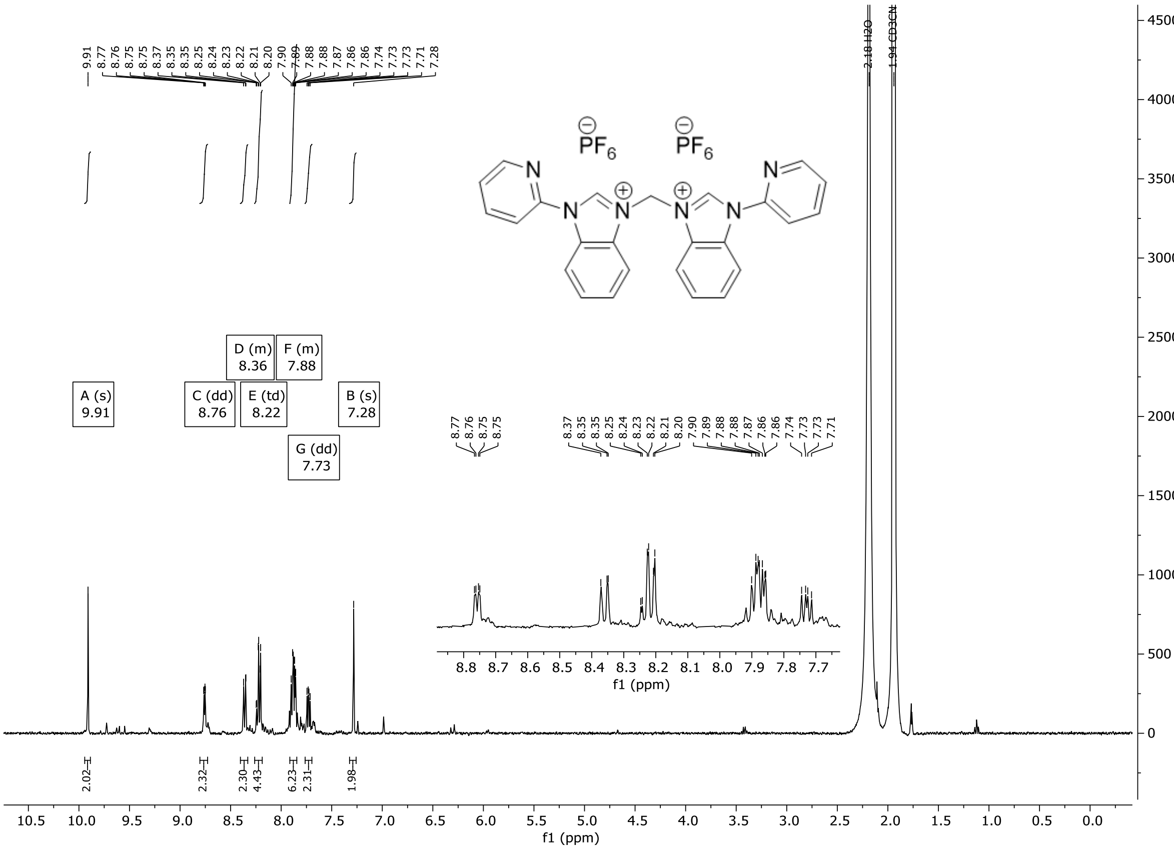

Figure 2 SI. ^{1}H NMR spectrum of $[H_2L1](PF_6)_2$ in CD_3CN.

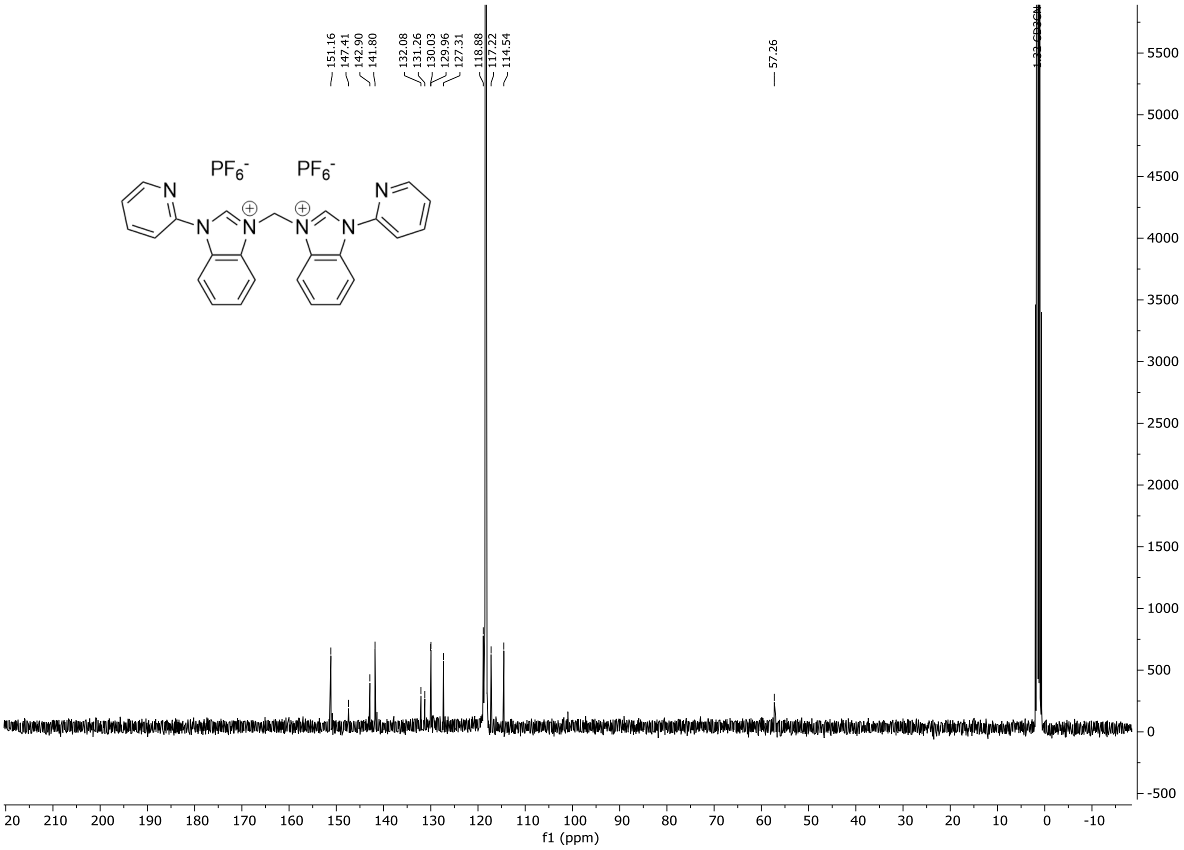

Figure 3 SI. ^{13}C NMR spectrum of $[H_2L1](PF_6)_2$ in CD_3CN.

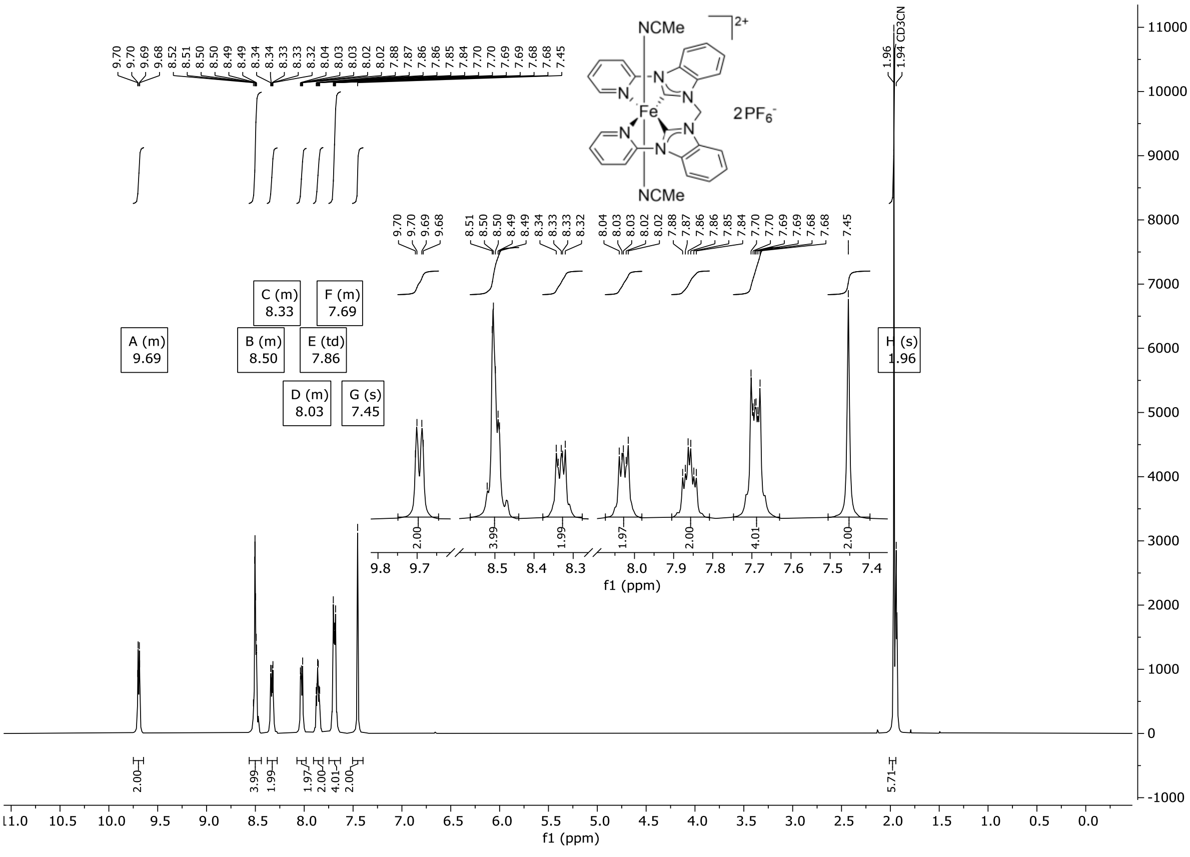

Figure 4 SI. ^{1}H NMR spectrum of $[FeL1(MeCN)_2](PF_6)_2$, **1**, in CD_3CN.

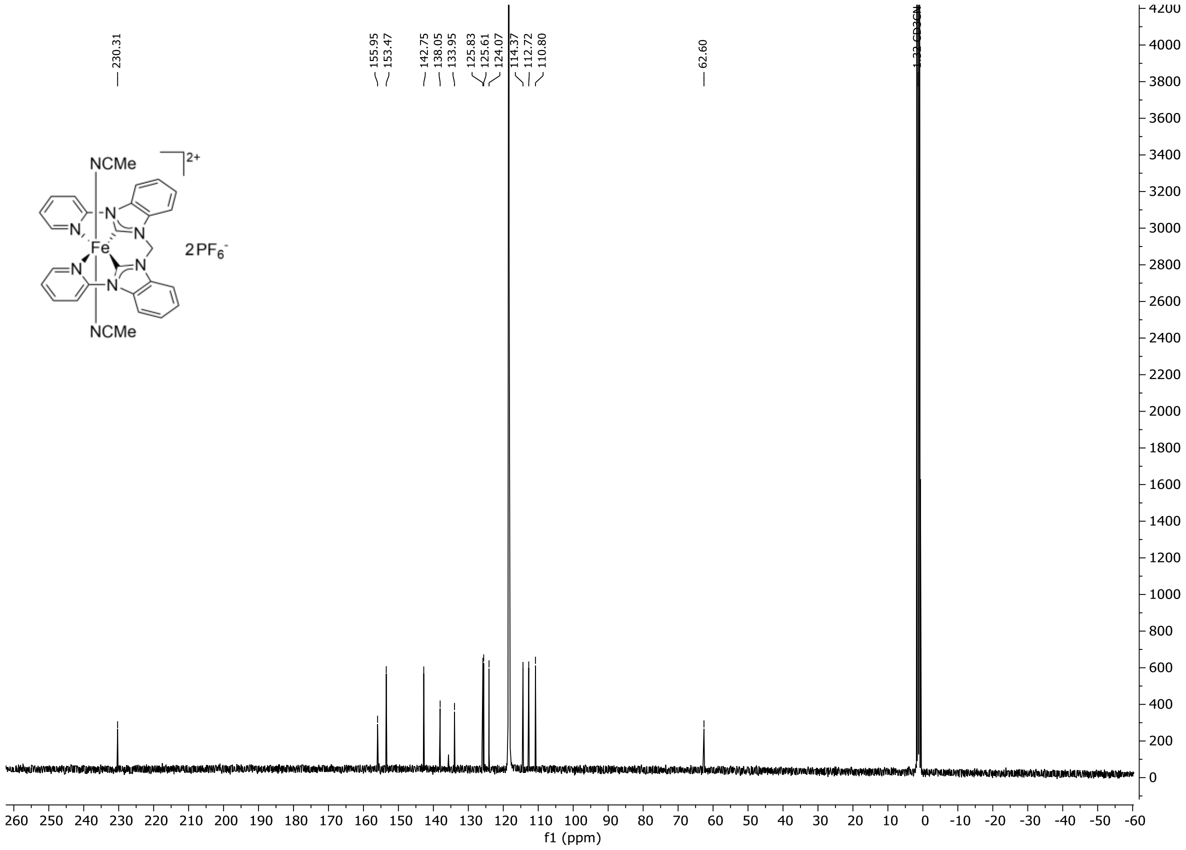

Figure 5 SI. ^{13}C NMR spectrum of $[FeL1(MeCN)_2](PF_6)_2$, **1**, in CD_3CN.

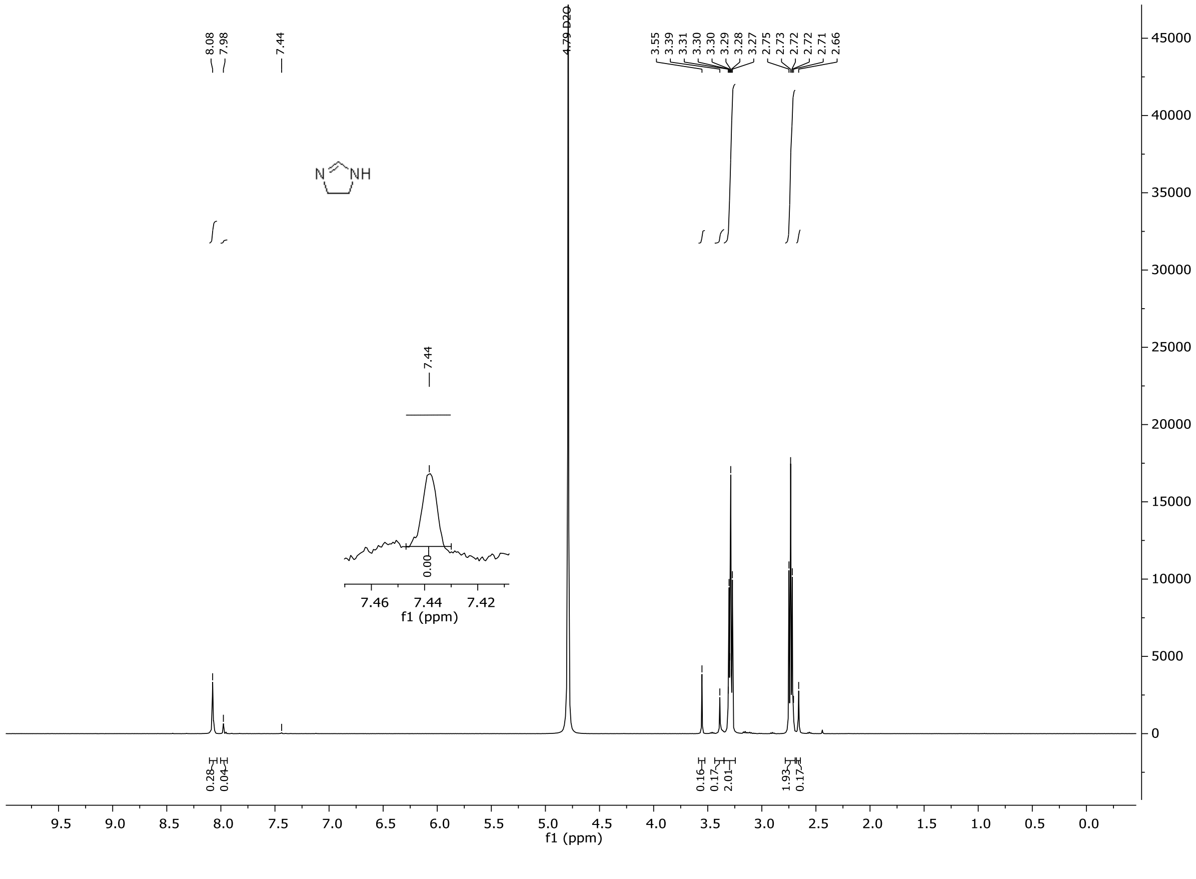

Figure 6 SI. ^{1}H NMR spectrum of 2-imidazoline in D_2O.[1]

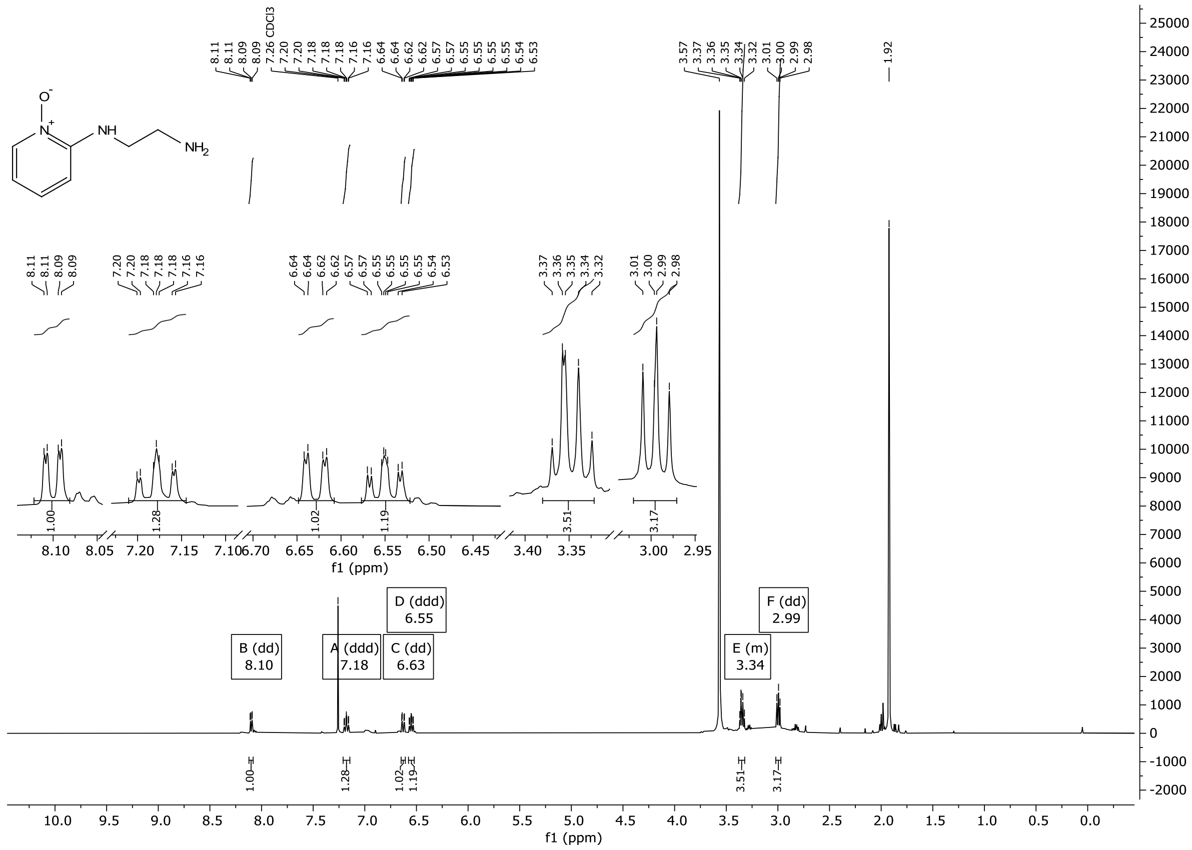

Figure 7 SI. ^{1}H NMR spectrum of 2-((2-aminoethyl)amino)pyridine 1-oxide in $CDCl_3$.

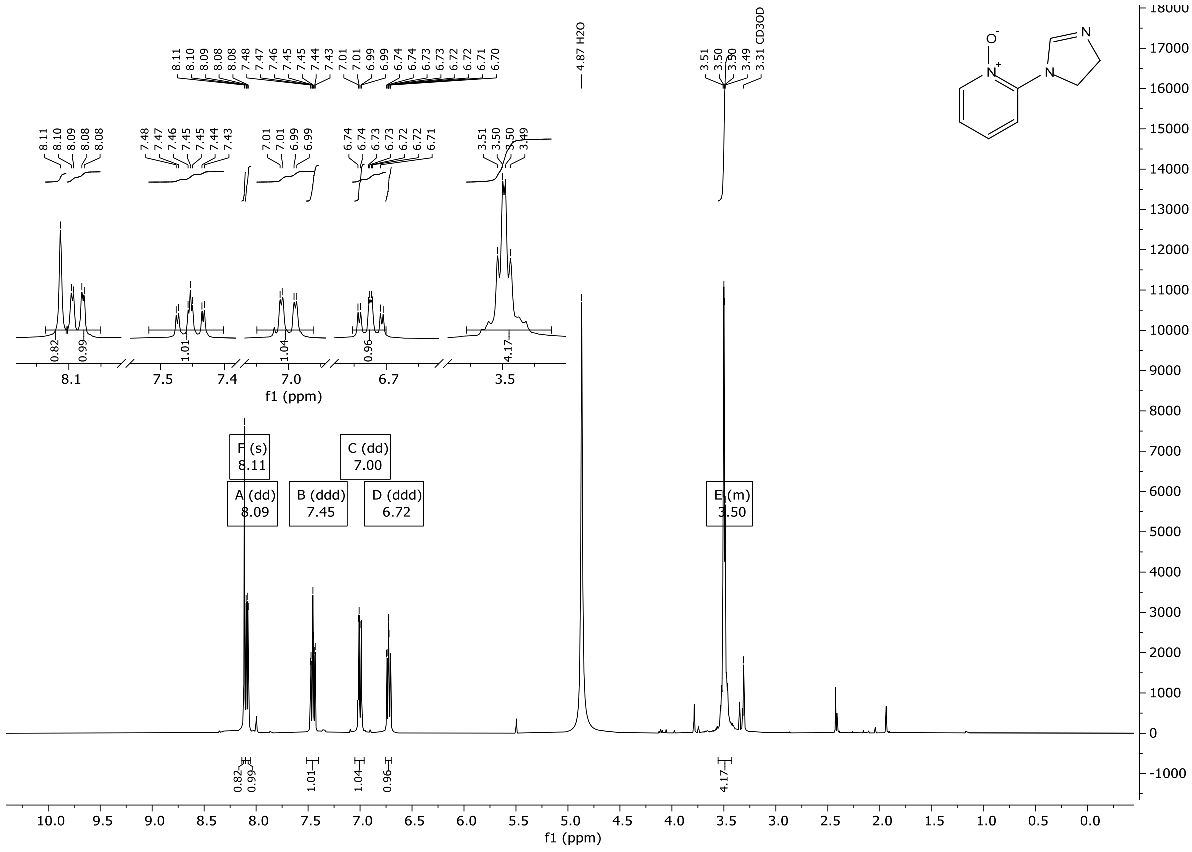

Figure 8 SI. ^{1}H NMR spectrum of 2-(2-imidazolin-1-yl)pyridine 1-oxide in CD_3OD.

2. ESI-MS spectra

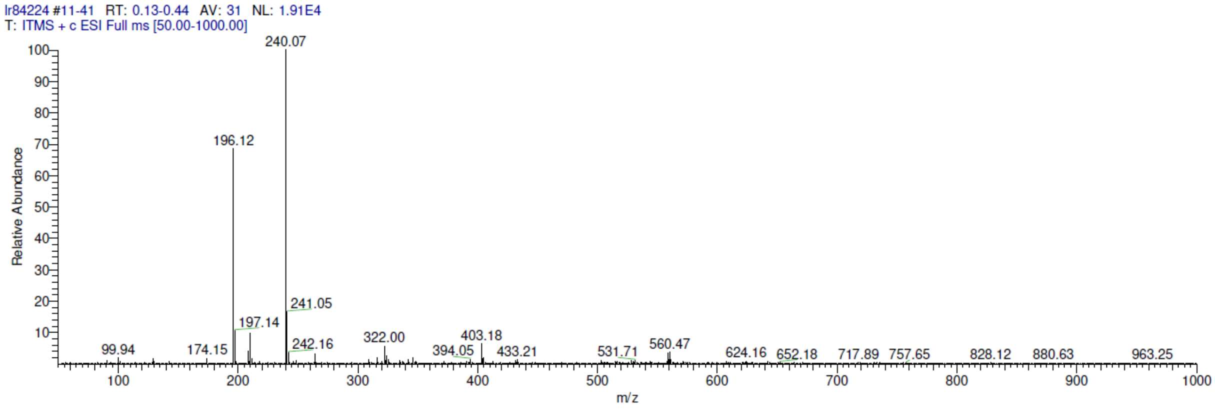

Figure 9 SI. ESI-MS spectrum of $[H_2L1](Br)_2$.

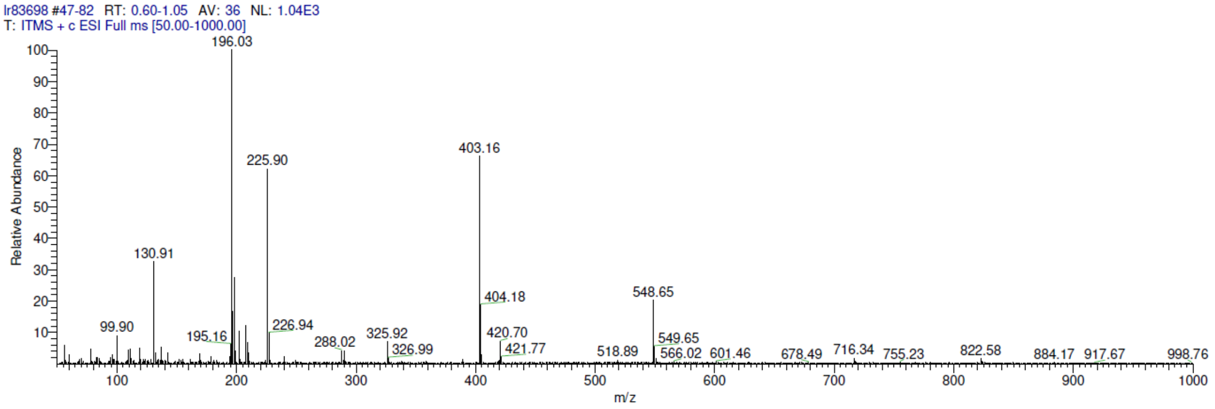

Figure 10 SI. ESI-MS spectrum of $[H_2L1](PF_6)_2$.

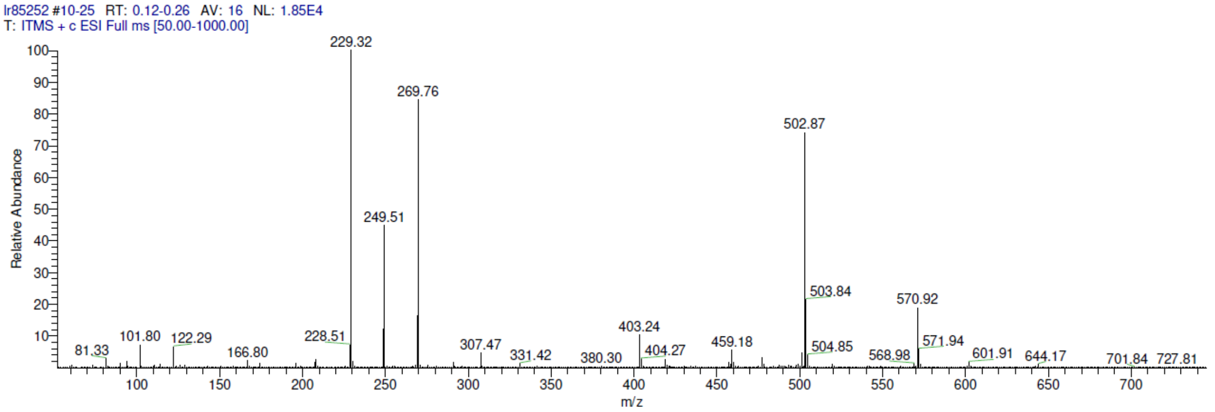

Figure 11 SI. ESI-MS spectrum of $[FeL1(MeCN)_2](PF_6)_2$, **1**.

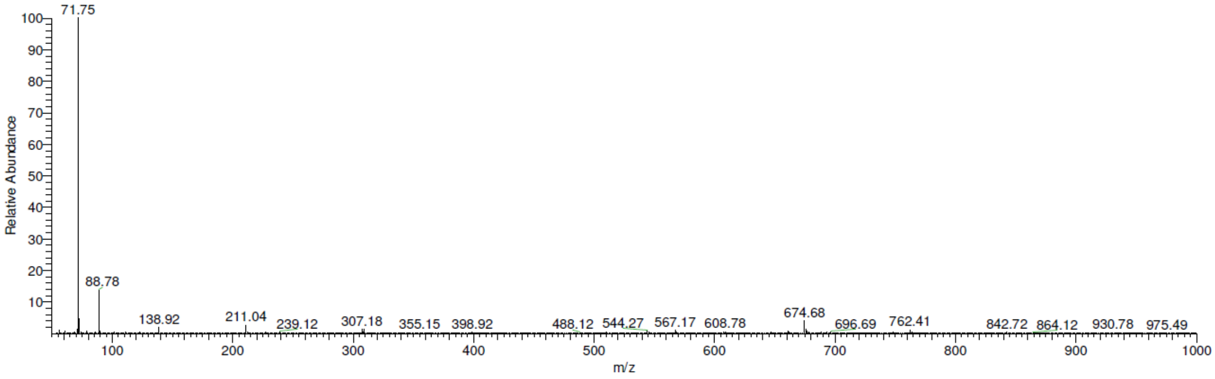

Figure 12 SI. ESI-MS spectrum of 2-imidazoline.[1]

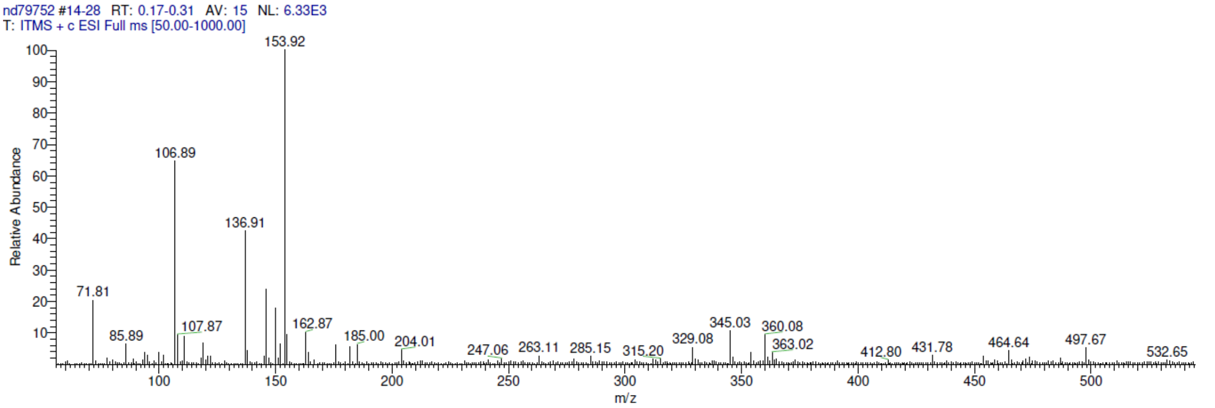

Figure 13 SI. ESI-MS spectrum of 2-((2-aminoethyl)amino)pyridine 1-oxide.

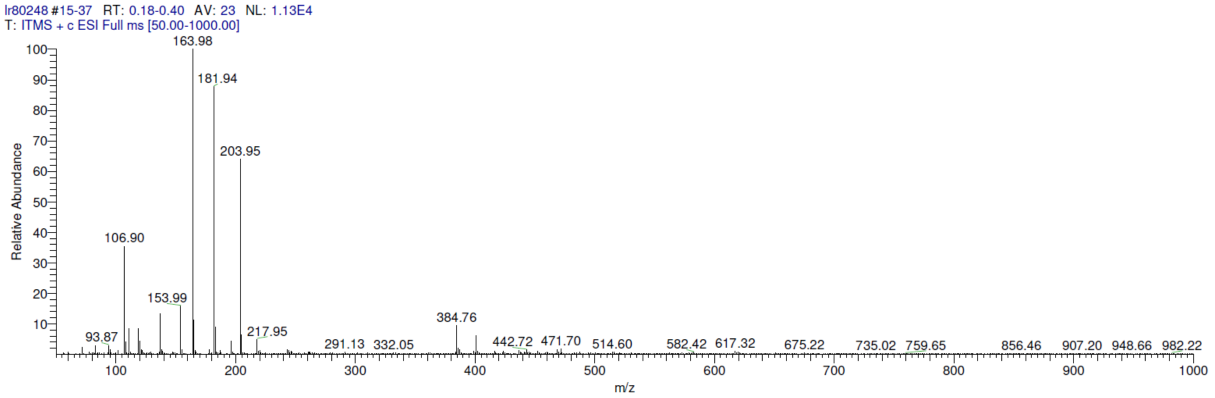

Figure 14 SI. ESI-MS spectrum of 2-(2-imidazolin-1-yl)pyridine 1-oxide.

3. Crystallographic data

X-ray crystallographic data was collected on a single crystal x-ray diffractometer with the following setup: A CMOS detector (Bruker APEX III, κ-CMOS), a TXS rotating anode and a Helios optic using the APEX4 software package.[2] The measurements used MoK$_{\alpha}$ radiation (λ = 0.71073 Å) and were performed on single crystals coated with perfluorinated ether. The crystals were fixed on top of a micromount sample holder and frozen under a stream of cold nitrogen at 100 K. Matrix scans were used to determine the initial lattice parameters. Reflections were corrected for Lorentz and polarization effects, scan speed, and background using SAINT.[3] Absorption corrections, including odd and even ordered spherical harmonics were performed using SADABS.[4] Space group assignments were based upon systematic absences, E statistics, and successful refinement of the structures. The structures were solved by direct methods (SHELXT) with the aid of successive difference Fourier maps, and were refined against all data using SHELXL-2015 in conjunction with SHELXLE.[5-7] Hydrogen atoms were calculated in ideal positions as follows: Methyl hydrogen atoms were refined as part of rigid rotating groups, with a C–H distance of 0.98 Å and $U_{iso}(H) = 1.5 \cdot U_{eq}(C)$. Other H atoms were placed in calculated positions and refined using a riding model, with methylene, aromatic, and other C–H distances of 0.99 Å, 0.95 Å, and 1.00 Å, respectively and $U_{iso}(H) = 1.2 \cdot U_{eq}(C)$. Non-hydrogen atoms were refined with anisotropic displacement parameters. Full-matrix least-squares refinements were carried out by minimizing $\Sigma w(F_o^2 – F_c^2)^2$ with the SHELXL weighting scheme.[7] Neutral atom scattering factors for all atoms and anomalous dispersion corrections for the non-hydrogen atoms were taken from *International Tables for Crystallography.*[8] The images of the crystal structures were generated with Platon.[9] CCDC 2326822 (complex **1**) contains the supplementary crystallographic data for this paper. This data can be obtained free of charge *via* www.ccdc.cam.ac.uk/data_request/cif, or by emailing data_request@ccdc.cam.ac.uk, or by contacting The Cambridge Crystallographic Data Centre, 12 Union Road, Cambridge CB2 1EZ, UK; fax: +44 1223 336033.

Crystallographic data of 1

Single crystals suitable for X-ray diffraction were obtained by slow vapor diffusion of Et_2O (neither degassed nor dried) into a solution of **1** in MeCN (neither degassed nor dried) after 2 weeks at r.t. under ambient atmosphere (see Figure 15 SI):

Three NMR tubes were placed into a Schlenk tube. Around 3 mg of **1** were dissolved in 0.2 mL MeCN (neither degassed nor dried) under ambient atmosphere. The solution was filtered through a syringe filter and distributed evenly among the three NMR tubes (around 0.06 mL each). The first two NMR tubes were diluted with a total of 0.2 mL MeCN (neither degassed nor dried and filtered through a syringe filter; around 0.07 mL MeCN added to the first and 0.13 mL MeCN added to the second NMR tube) to create three different concentrations of **1**. Et_2O (neither degassed nor dried) was added to the Schlenk tube, outside of the NMR tubes, up to a filling level of around 3 cm (~25 mL). The Schlenk tube was sealed under ambient atmosphere. Single crystals suitable for SC-XRD were obtained after 2 weeks at r.t.

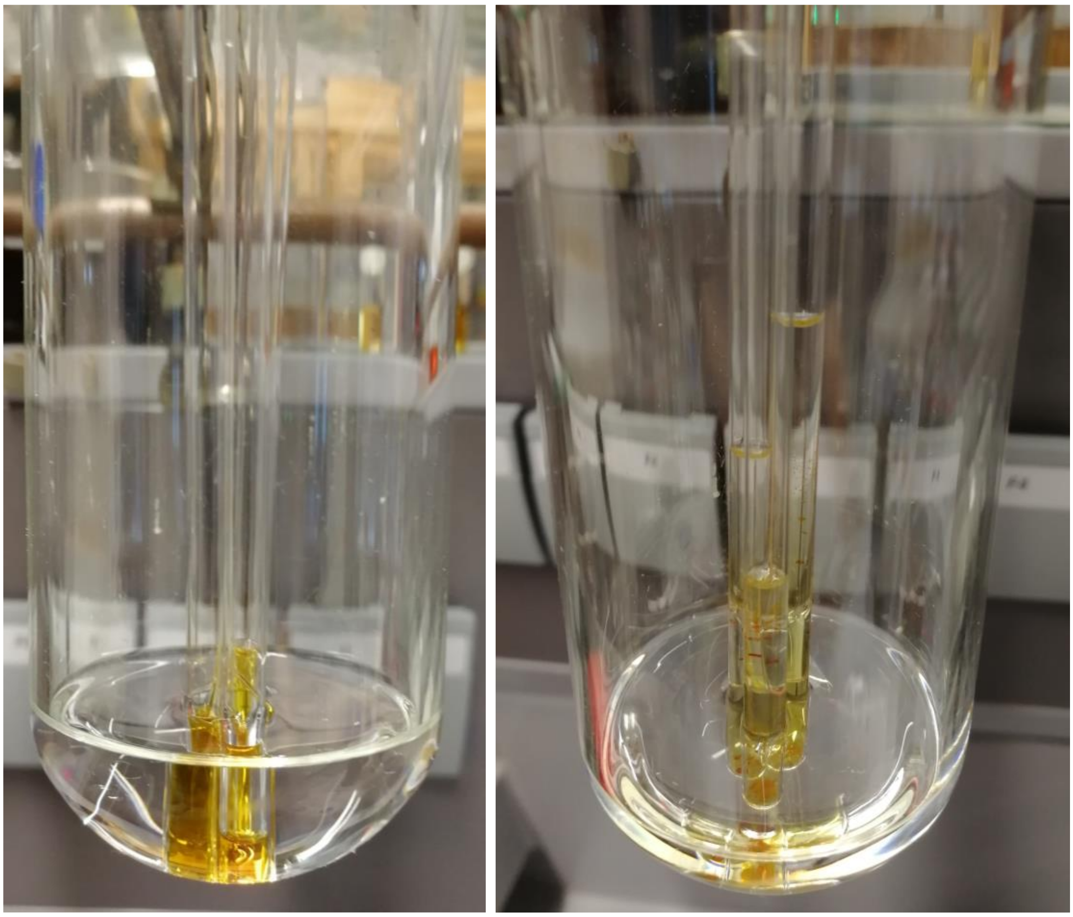

Figure 15 SI. Left: Crystallization setup of **1**. Right: First crystals are visible in the NMR tubes after 1 week. The filling level in the NMR tubes has increased due to the vapor diffusion of Et_2O into the NMR tubes over the course of 1 week.

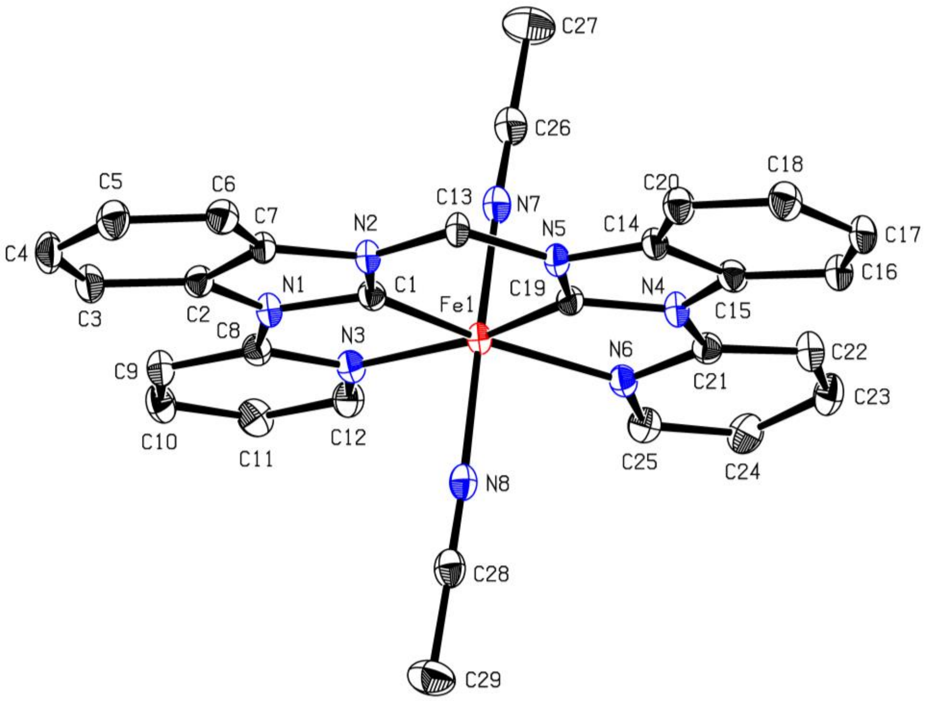

Figure 16 SI. ORTEP-style representation of **1**. Hydrogen atoms and hexafluorophosphate anions are omitted for clarity. Thermal ellipsoids are shown at a 50% probability level.

Selected bond lengths (Å):

C1-Fe1 1.8314(19); N3-Fe1 2.0721(16); C19-Fe1 1.8293(19); N6-Fe1 2.0852(16); N7-Fe1 1.9136(18); N8-Fe1 1.9253(18)

Selected bond angles (°):

N2-C1-Fe1 134.06(14); N1-C1-Fe1 119.17(14); N5-C19-Fe1 133.93(14); N4-C19-Fe1 119.63(14); C12-N3-Fe1 129.81(13); C8-N3-Fe1 113.03(13); C25-N6-Fe1 130.44(13); C21-N6-Fe1 112.64(13); C26-N7-Fe1 177.57(17); C28-N8-Fe1 175.27(17); C19-Fe1-C1 87.14(8); C19-Fe1-N7 91.24(8); C1-Fe1-N7 95.12(8); C19-Fe1-N8 95.10(8); C1-Fe1-N8 91.09(8); N7-Fe1-N8 171.34(7); C19-Fe1-N3 166.98(8); C1-Fe1-N3 79.90(7); N7-Fe1-N3 88.61(7); N8-Fe1-N3 86.53(7); C19-Fe1-N6 79.58(7); C1-Fe1-N6 166.66(8); N7-Fe1-N6 86.60(7); N8-Fe1-N6 88.74(7); N3-Fe1-N6 113.40(6)

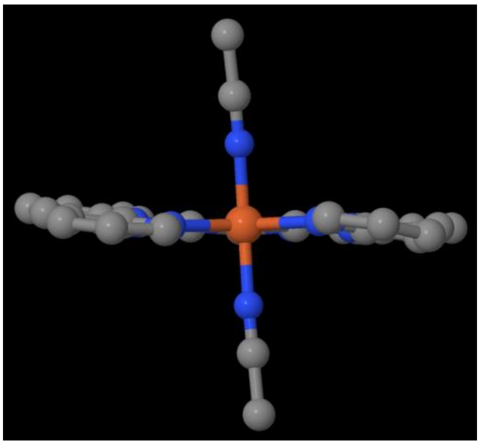

Figure 17 SI. Visualization of complex **1** (*Jmol*[10]). The planar equatorial NCCN ligand is visible.

4. Cyclic voltammetry

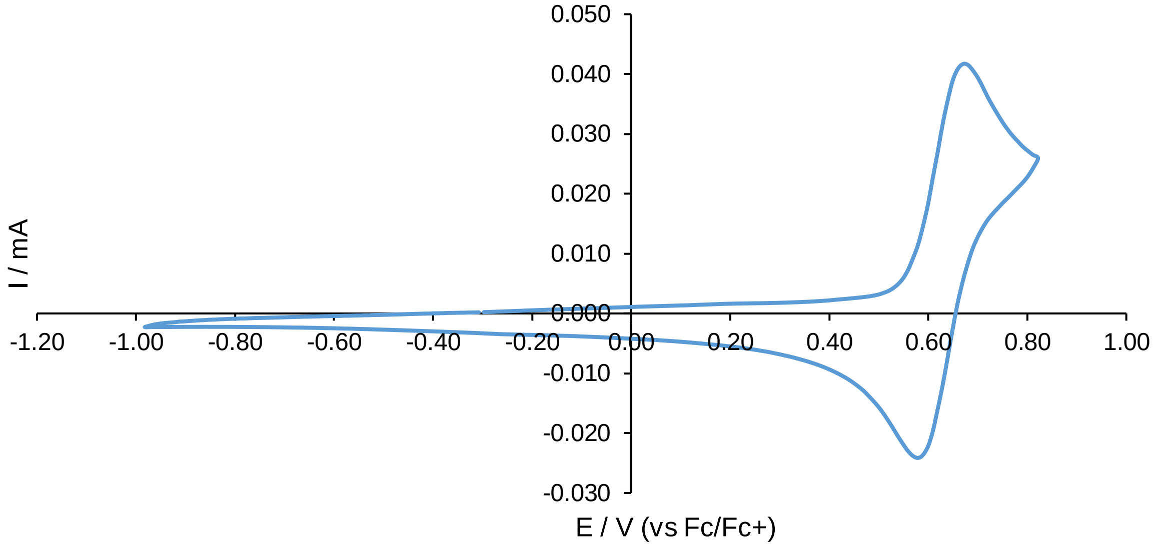

Figure 18 SI. Cyclic voltammetry of **1** in MeCN under argon atmosphere at 100 mV/s. Half-cell potential is determined to $E_{1/2}$ = 0.625 V (V *versus* Fc/Fc^{+}) and oxidation/reduction potentials are determined to E_{ox} = 0.670 V and E_{red} = 0.579 V. ΔE = 0.091 V.

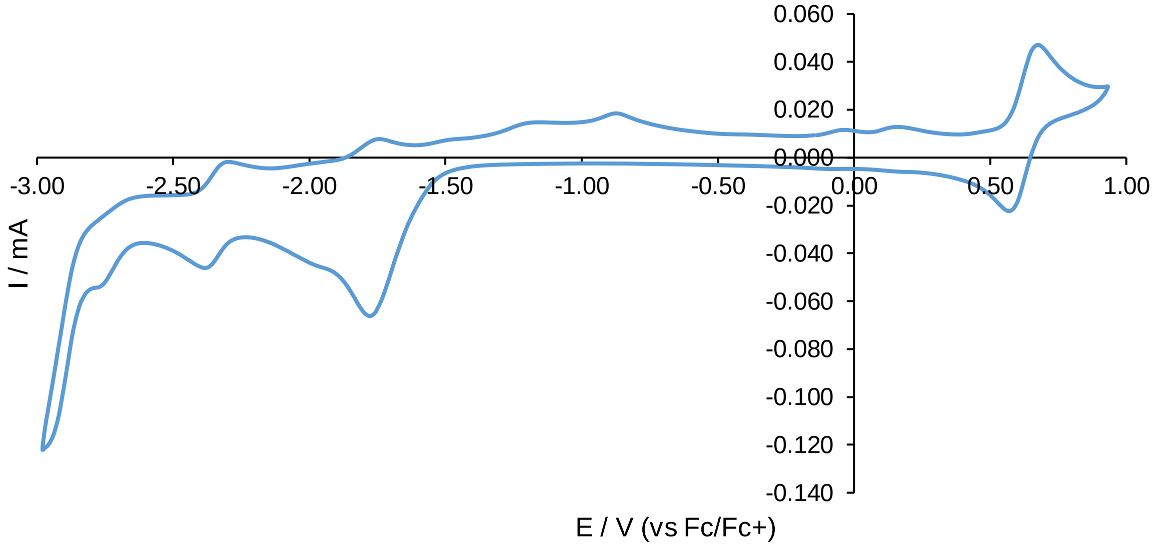

Figure 19 SI. Cyclic voltammetry of **1** in MeCN under argon atmosphere at 100 mV/s over a broad range of V.

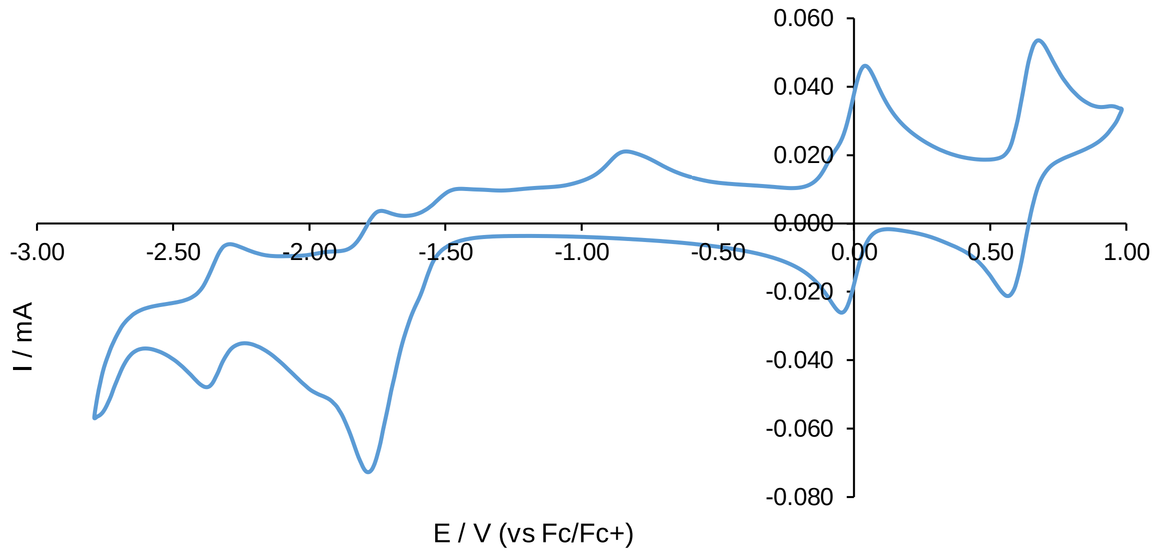

Figure 20 SI. Cyclic voltammetry of **1** in MeCN under argon atmosphere at 100 mV/s over a broad range of V. Half-cell potential of **1** is determined to $E_{1/2}$ = 0.625 V. Fc/Fc$^+$ is present at $E_{1/2}$ = 0.000 V.

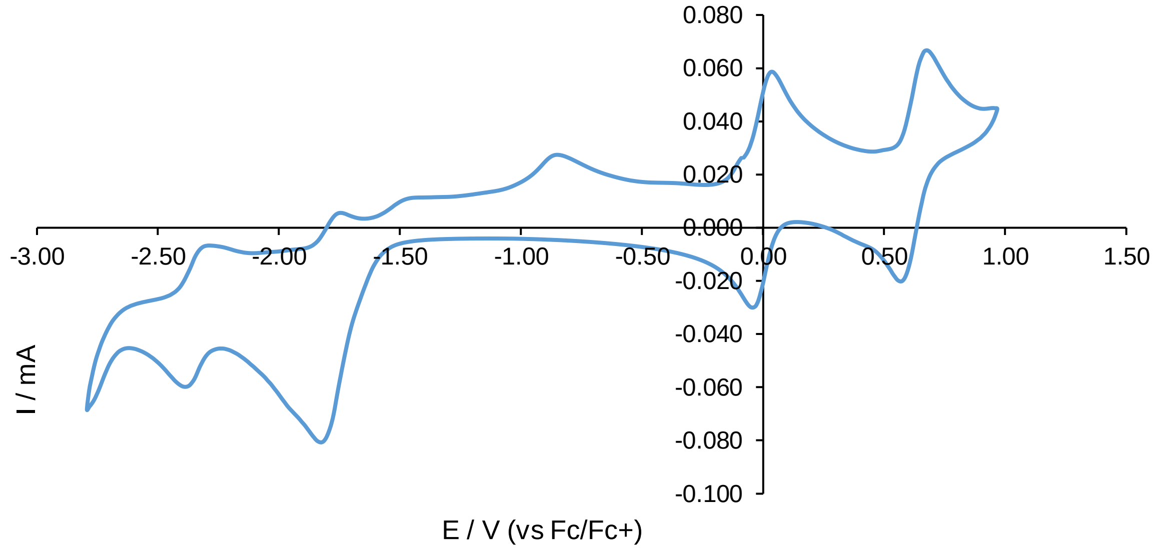

Figure 21 SI. Cyclic voltammetry of **1** in MeCN after bubbling N_2 for 5 min into the solution to exchange the atmosphere with nitrogen. 100 mV/s over a broad range of V. Fc/Fc$^+$ is present at $E_{1/2}$ = 0.000 V.

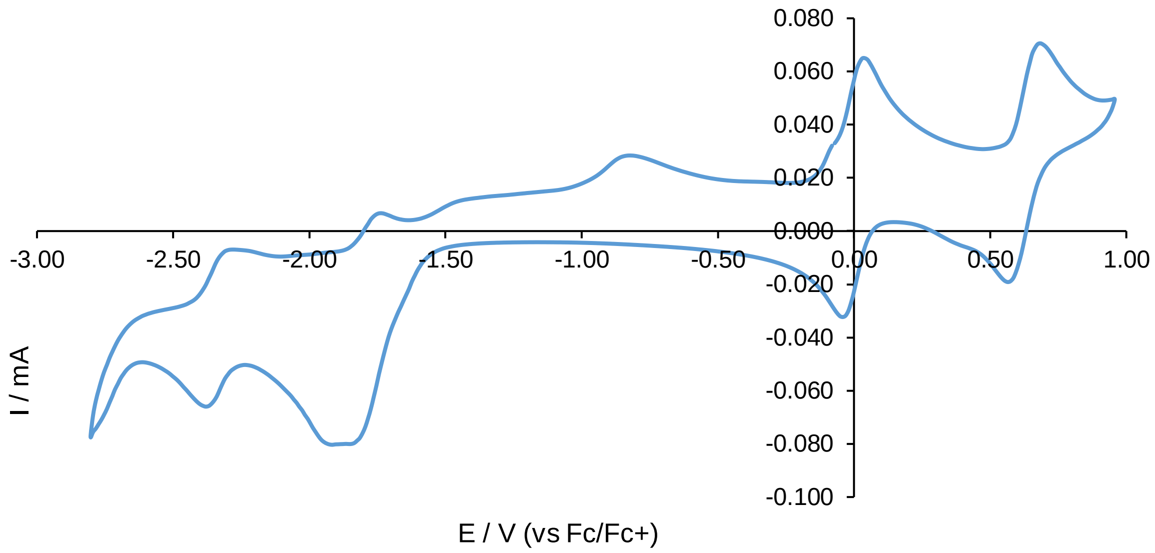

Figure 22 SI. Cyclic voltammetry of **1** in MeCN after bubbling N_2 for a second time for 5 min into the solution (measurement still under N_2). 100 mV/s over a broad range of V. Fc/Fc^+ is present at $E_{1/2}$ = 0.000 V.

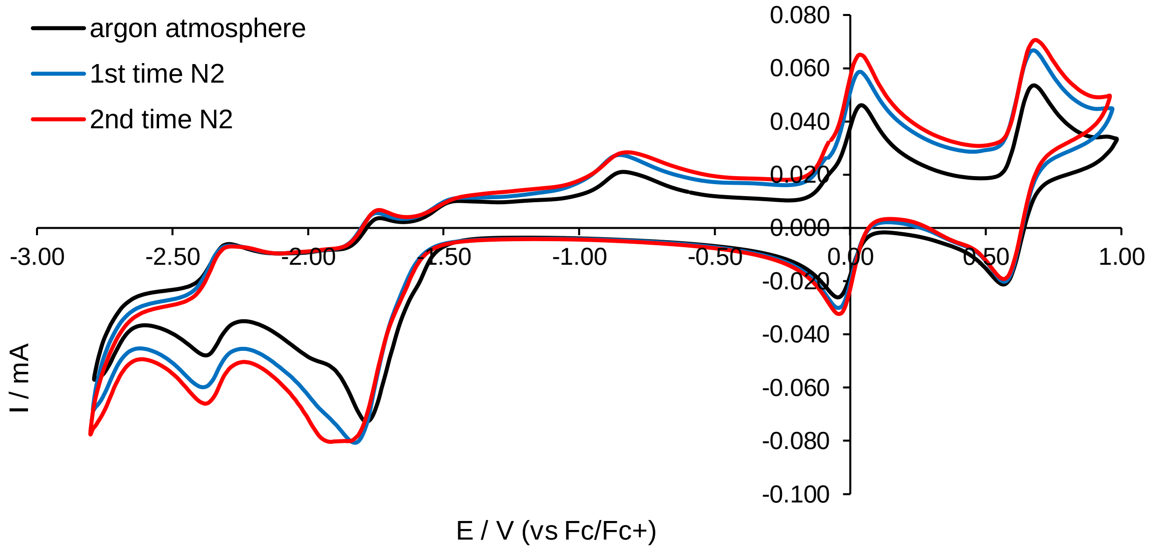

Figure 23 SI. Cyclic voltammetry of **1** in MeCN in argon atmosphere (black), after bubbling N_2 for 5 min into the solution to exchange the atmosphere with nitrogen (blue) and after a break of 15 min and bubbling N_2 for a second time for 5 min into the solution (total 20 min, measurement still under N_2, red). 100 mV/s over a broad range of V. Fc/Fc^+ is present at $E_{1/2}$ = 0.000 V. During bubbling, part of the solvent is evaporated, resulting in a higher concentration from time to time, which in turn is reflected in a higher current flow I in mA during the redox processes.

5. UV/Vis spectroscopy

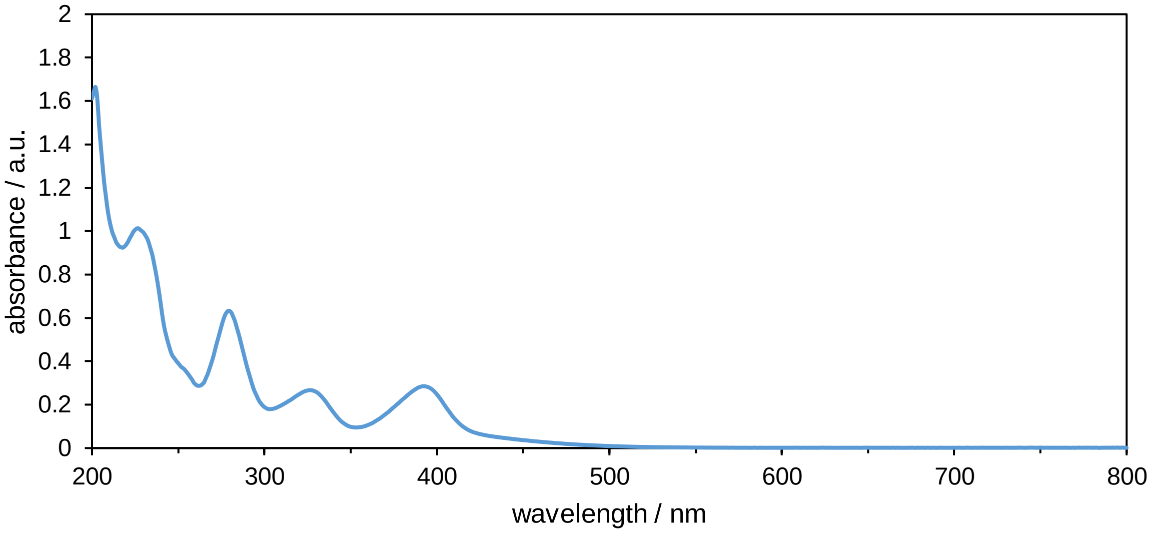

Figure 24 SI. UV/Vis spectrum of **1** (blue) in MeCN; *T* = 20 °C; c = 0.02 mM. Local absorption maxima at 227 nm, 279 nm, 327 nm and 393 nm.

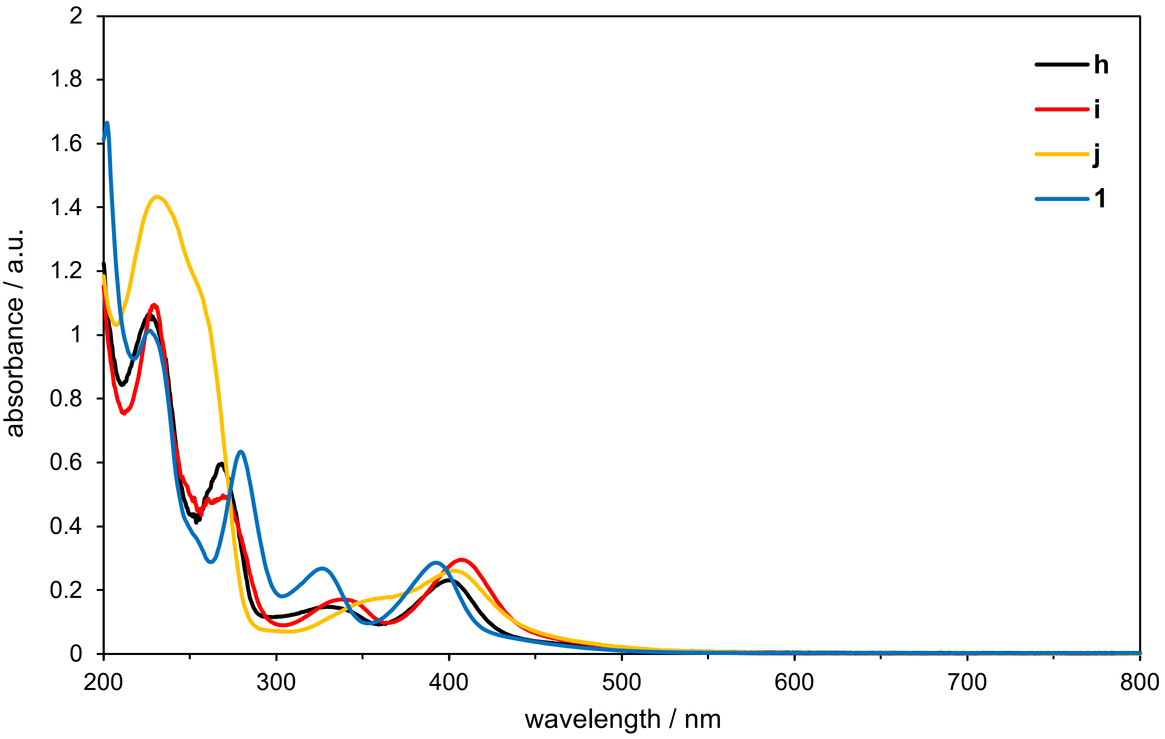

Figure 25 SI. UV/Vis spectrum of **1** (blue) in MeCN; *T* = 20 °C; c = 0.02 mM. Local absorption maxima at 227 nm, 279 nm, 327 nm and 393 nm. UV/Vis spectrum of **h** (black), **i** (red) and **j** (yellow) in MeCN; *T* = 20 °C; c = 0.2 mM.[11]

6. Buried volume and topographic steric map calculations

The determination of the buried volume[12] and the topographic steric maps[13] for complex **1** was performed using *SambVca 2.1*[14]. The structure of the complex was loaded using the respective .xyz files derived from the SC-XRD analysis. The iron atom was set as center of the sphere (distance 0.00 Å).

a) For calculations with viewing direction towards the opening of the NCCN ligand: The methylene bridge *C*-atom of the NCCN ligand was selected for the Z-negative z axis definition. One *N*-atom of the two axial MeCN ligands was selected for the xz-plane definition.
b) For calculations with viewing direction on the top of the complex, *i.e.* axial MeCN ligand vertically facing towards the viewer and the NCCN ligand underneath: One *N*-atom of the two axial MeCN ligands was selected for the Z-negative z axis definition. The methylene bridge *C*-atom of the NCCN ligand was selected for the xz-plane definition.

The iron atom was selected to be deleted. The atomic radii were left on the presetting (bond radii scaled by 1.17). The sphere radius was left on presetting (3.5 Å). Distance of the coordination point from the center of the sphere was set to 0.0 Å (presetting). Mesh spacing for numerical integration was set to 0.10 Å (presetting). H atoms were not included in the calculations (presetting).

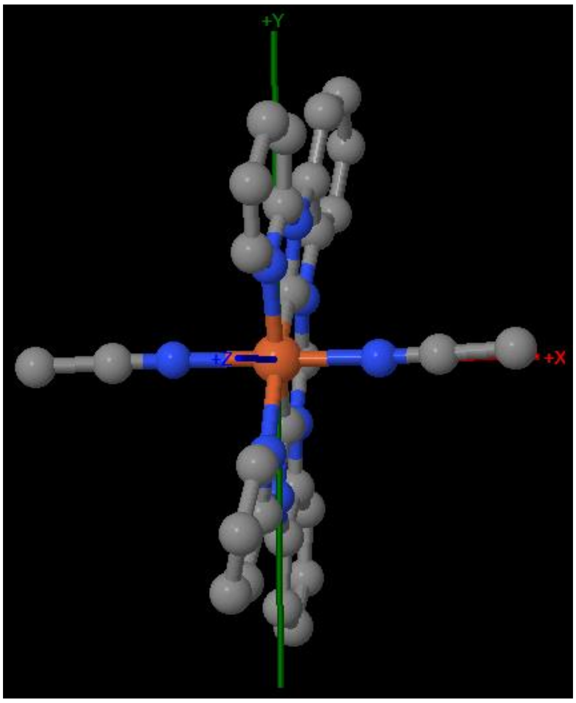

Figure 26 SI. Visualization of complex **1** (*JSmol* window) and assignment of the x, y, z axes.

Table 1 SI. Calculated buried volume for **1**.

**%V Free**	**%V Buried**	**% V Tot/V Ex**
13.6	**86.4**	99.9

**Quadrant**	**V f**	**V b**	**V t**	**%V f**	**%V b**
SW	5.2	39.7	44.9	**11.6**	88.4
NW	6.8	38.1	44.9	**15.1**	84.9
NE	5.2	39.6	44.9	**11.7**	88.3
SE	7.1	37.8	44.9	**15.8**	84.2

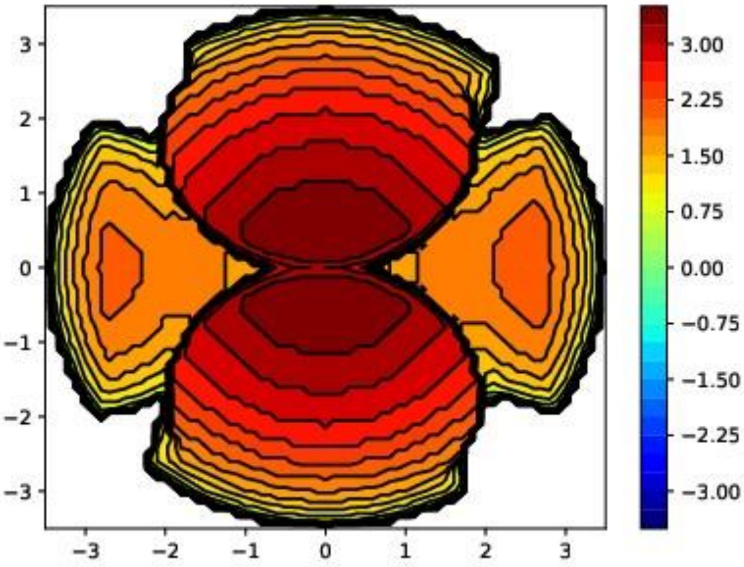

Figure 27 SI. Topographic steric map of the buried volume of **1**. The red and blue colors show the more- and less-hindered zones in the catalytic center, respectively.

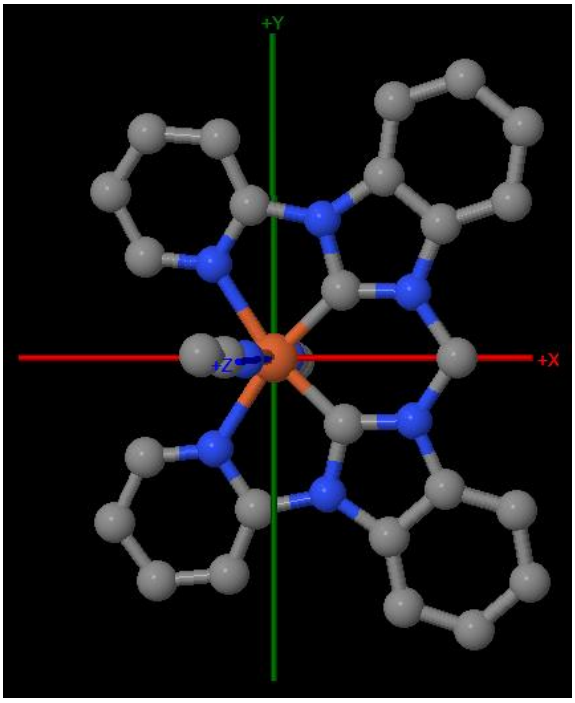

Figure 28 SI. Visualization of complex **1** (*JSmol* window) and assignment of the x, y, z axes.

Table 2 SI. Calculated buried volume for **1**.

**%V Free**	**%V Buried**	**% V Tot/V Ex**
13.6	**86.4**	99.9

**Quadrant**	**V f**	**V b**	**V t**	**%V f**	**%V b**
SW	5.7	39.2	44.9	**12.7**	87.3
NW	5.8	39.1	44.9	**12.9**	87.1
NE	6.5	38.3	44.9	**14.6**	85.4
SE	6.3	38.6	44.9	**14.1**	85.9

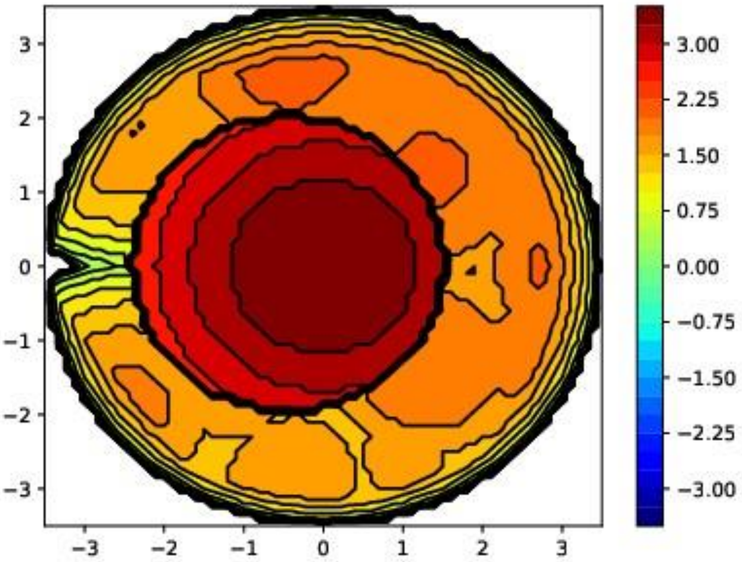

Figure 29 SI. Topographic steric map of the buried volume of **1**. The red and blue colors show the more- and less-hindered zones in the catalytic center, respectively.

7. Catalytic olefin epoxidation reactions of *cis*-cyclooctene

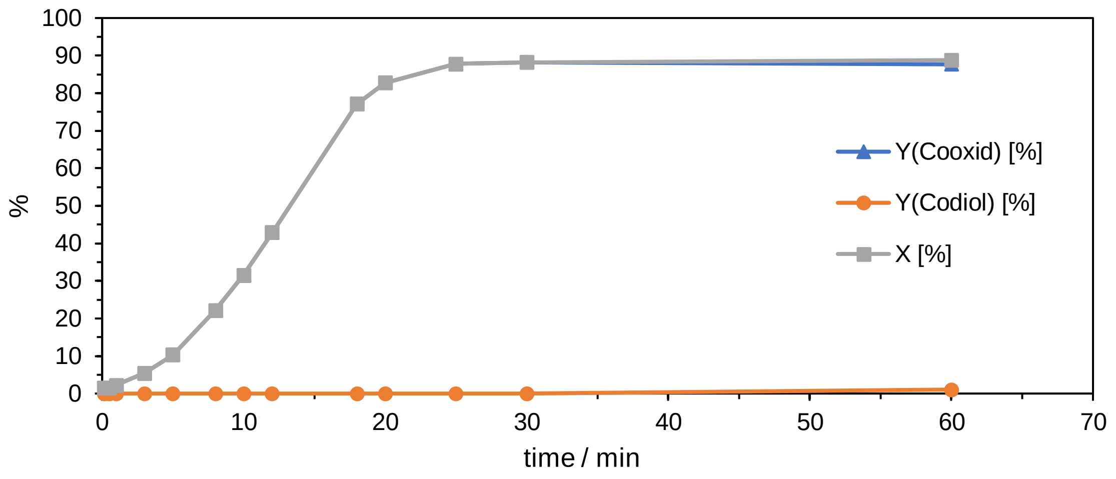

Figure 30 SI. Time-dependent epoxidation of *cis*-cyclooctene (67.3 µmol/mL, 1.00 eq.) in MeCN using **1** (1.35 µmol/mL, 0.02 eq.) as catalyst, $Sc(OTf)_3$ (6.73 µmol/mL, 0.10 eq.) and H_2O_2 (50% aq., 101 µmol/mL, 1.50 eq.) as oxidizing agent at 20 °C. Conversions are determined by GC-FID. X = conversion. Y(Cooxid) = Yield cyclooctene oxide. Y(Codiol) = Yield cyclooctane-1,2-diol.

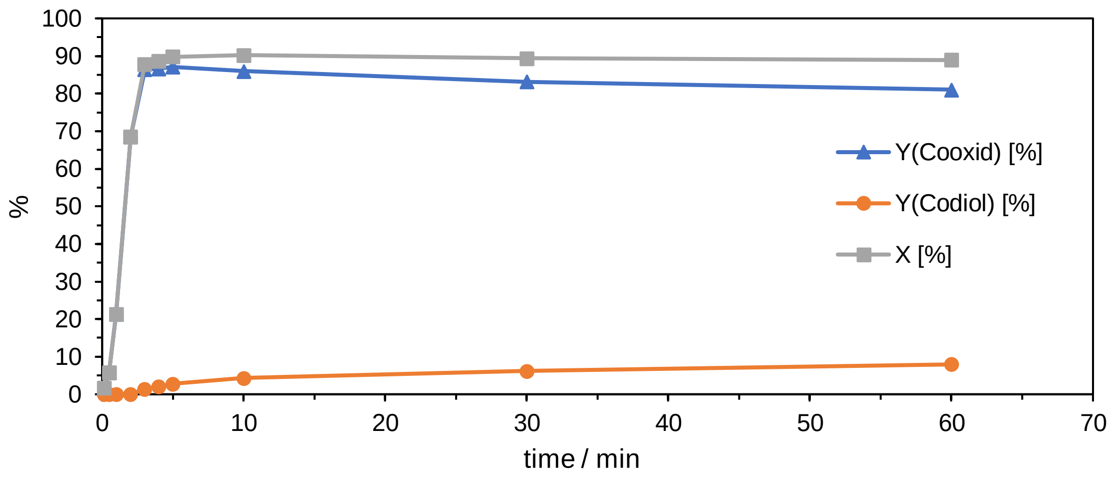

Figure 31 SI. Time-dependent epoxidation of *cis*-cyclooctene (67.3 µmol/mL, 1.00 eq.) in MeCN using **1** (1.35 µmol/mL, 0.02 eq.) as catalyst, $Sc(OTf)_3$ (6.73 µmol/mL, 0.10 eq.) and H_2O_2 (50% aq., 101 µmol/mL, 1.50 eq.) as oxidizing agent at 40 °C. Conversions are determined by GC-FID. X = conversion. Y(Cooxid) = Yield cyclooctene oxide. Y(Codiol) = Yield cyclooctane-1,2-diol.

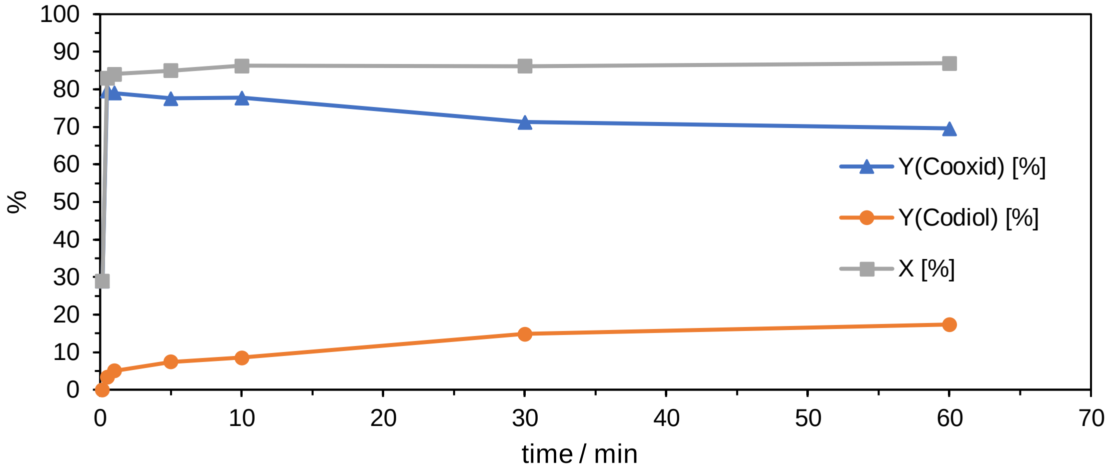

Figure 32 SI. Time-dependent epoxidation of *cis*-cyclooctene (67.3 µmol/mL, 1.00 eq.) in MeCN using **1** (1.35 µmol/mL, 0.02 eq.) as catalyst, $Sc(OTf)_3$ (6.73 µmol/mL, 0.10 eq.) and H_2O_2 (50% aq., 101 µmol/mL, 1.50 eq.) as oxidizing agent at 60 °C. Conversions are determined by GC-FID. X = conversion. Y(Cooxid) = Yield cyclooctene oxide. Y(Codiol) = Yield cyclooctane-1,2-diol.

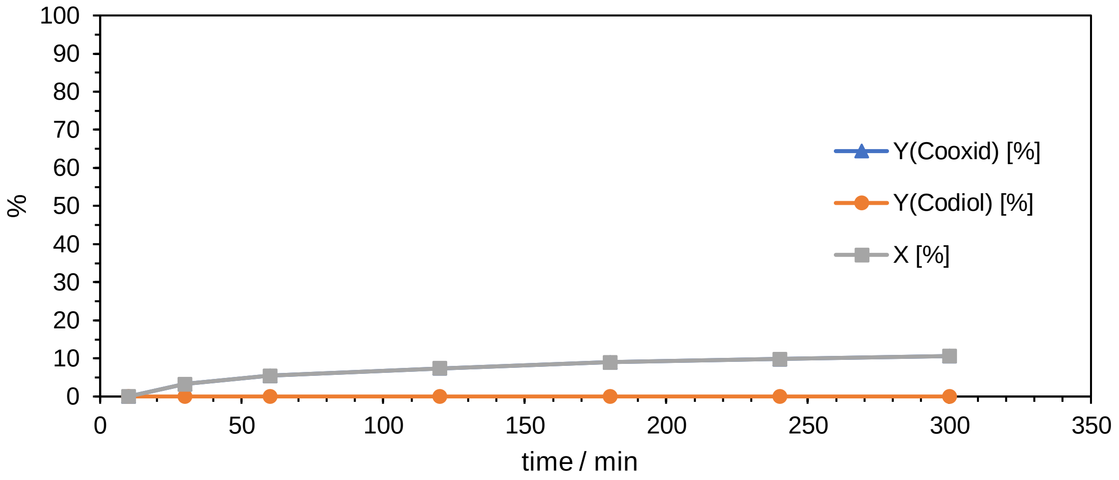

Figure 33 SI. Time-dependent epoxidation of *cis*-cyclooctene (67.3 µmol/mL, 1.00 eq.) in MeCN using **1** (1.35 µmol/mL, 0.02 eq.) as catalyst and H_2O_2 (50% aq., 101 µmol/mL, 1.50 eq.) as oxidizing agent at 40 °C. Conversions are determined by GC-FID. X = conversion. Y(Cooxid) = Yield cyclooctene oxide. Y(Codiol) = Yield cyclooctane-1,2-diol.

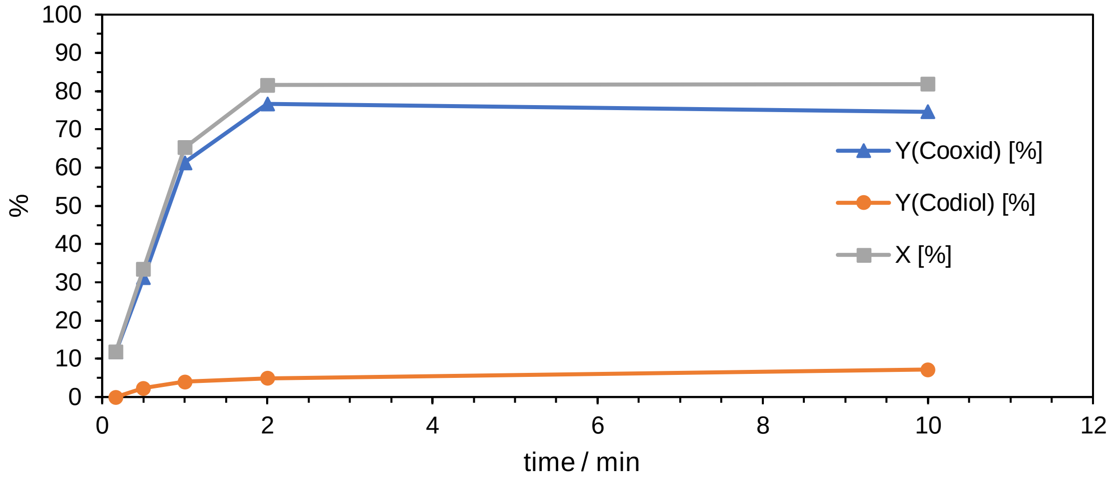

Figure 34 SI. Time-dependent epoxidation of *cis*-cyclooctene (67.3 µmol/mL, 1.00 eq.) in MeCN using **1** (0.005 eq.) as catalyst, $Sc(OTf)_3$ (6.73 µmol/mL, 0.10 eq.) and H_2O_2 (50% aq., 101 µmol/mL, 1.50 eq.) as oxidizing agent at 60 °C. Conversions are determined by GC-FID. X = conversion. Y(Cooxid) = Yield cyclooctene oxide. Y(Codiol) = Yield cyclooctane-1,2-diol.

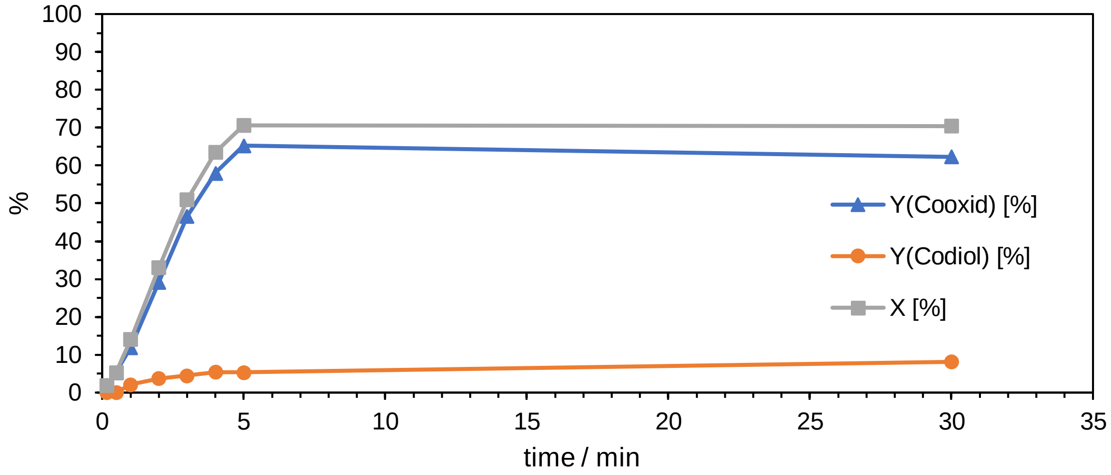

Figure 35 SI. Time-dependent epoxidation of *cis*-cyclooctene (67.3 µmol/mL, 1.00 eq.) in MeCN using **1** (0.001 eq.) as catalyst, $Sc(OTf)_3$ (6.73 µmol/mL, 0.10 eq.) and H_2O_2 (50% aq., 101 µmol/mL, 1.50 eq.) as oxidizing agent at 60 °C. Conversions are determined by GC-FID. X = conversion. Y(Cooxid) = Yield cyclooctene oxide. Y(Codiol) = Yield cyclooctane-1,2-diol.

8. ^{1}H NMR spectra of catalytic substrate screening

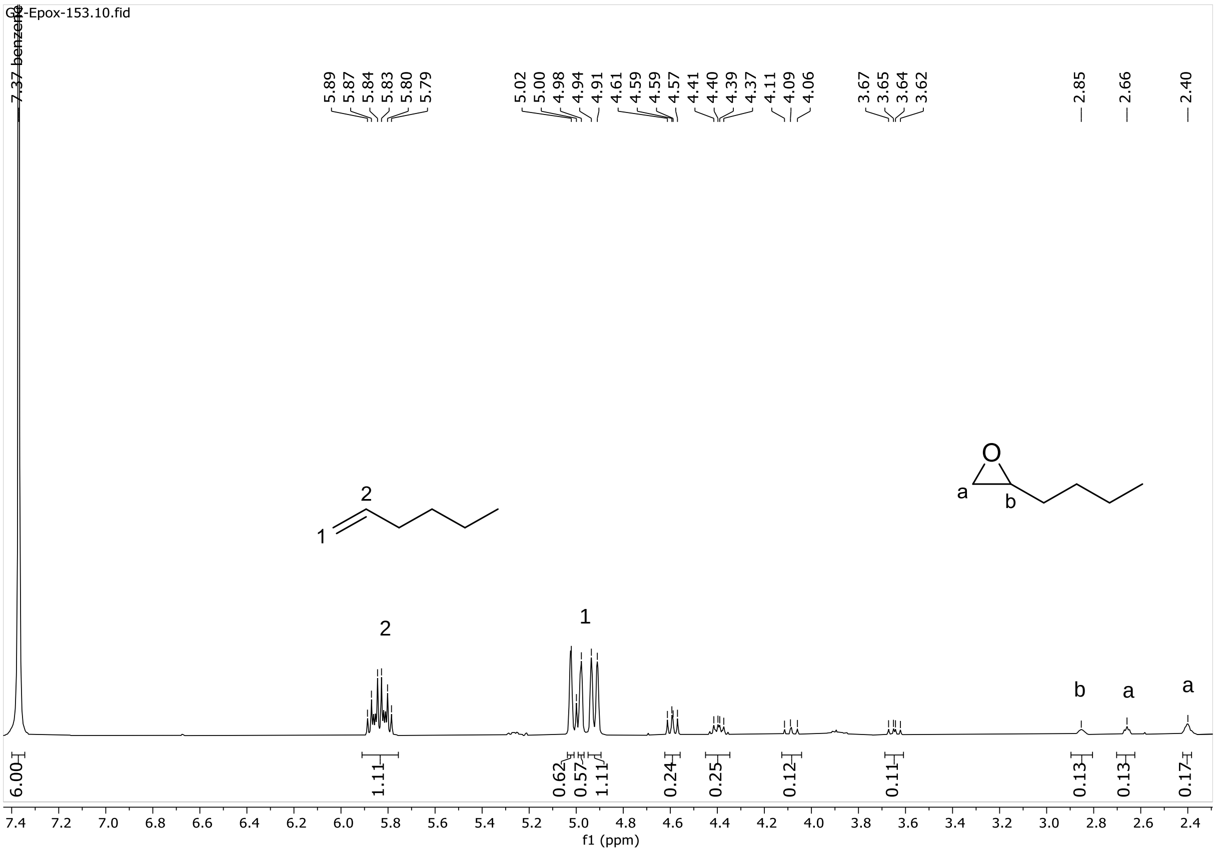

Figure 36 SI. Section of the ^{1}H NMR spectrum of the epoxidation of 1-hexene (1.00 eq., 67.3 µmol/mL) with H_2O_2 (50% aq., 1.50 eq., 101 µmol/mL), catalyst **1** (1.35 µmol/mL, 0.02 eq.) and $Sc(OTf)_3$ as additive (0.10 eq., 8.41 µmol/mL) at 20 °C in CD_3CN with a reaction time of 30 min. Estimation regarding the formation of the side products: diol (4.30 – 4.60 ppm) and aldehyde (3.60 – 4.30, 9.69 ppm).

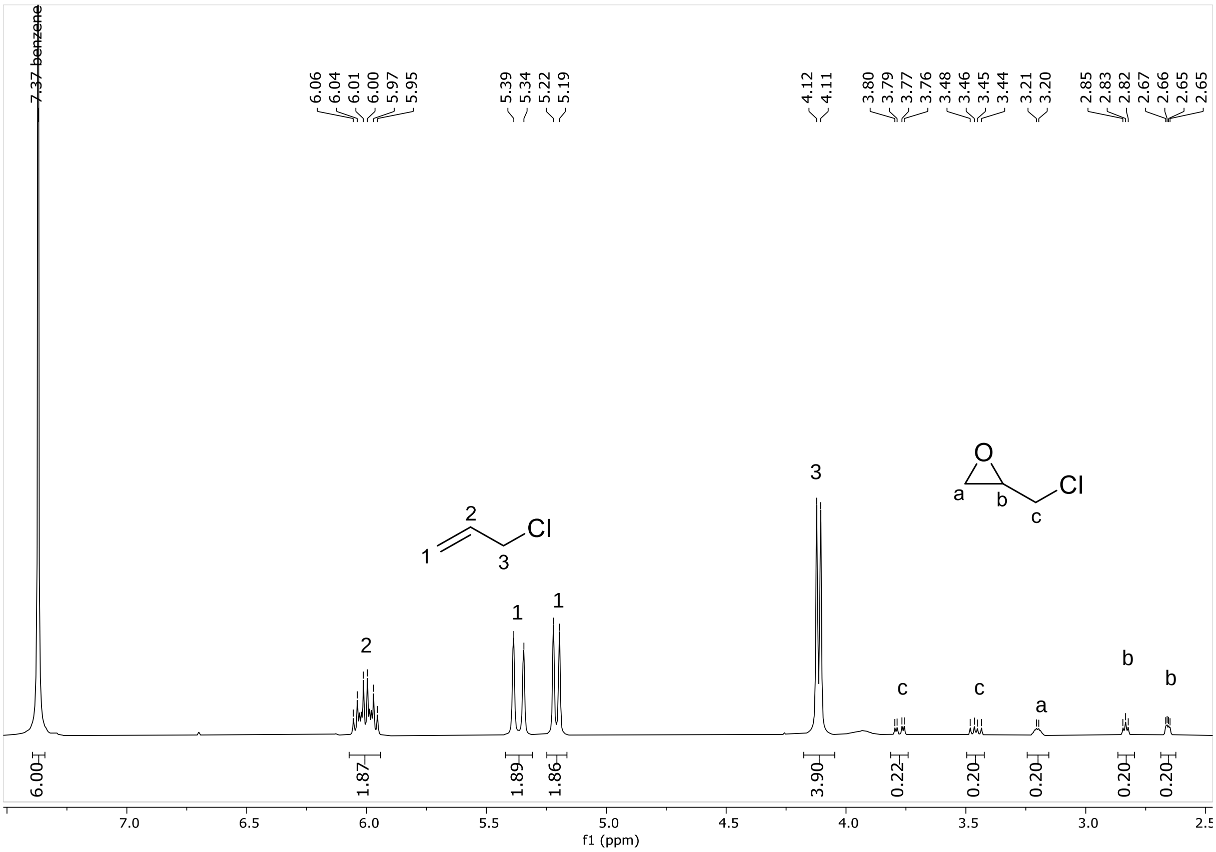

Figure 37 SI. Section of the ^{1}H NMR spectrum of the epoxidation of allyl chloride (1.00 eq., 67.3 µmol/mL) with H_2O_2 (50% aq., 1.50 eq., 101 µmol/mL), catalyst **1** (1.35 µmol/mL, 0.02 eq.) and $Sc(OTf)_3$ as additive (0.10 eq., 8.41 µmol/mL) at 20 °C in CD_3CN with a reaction time of 30 min.

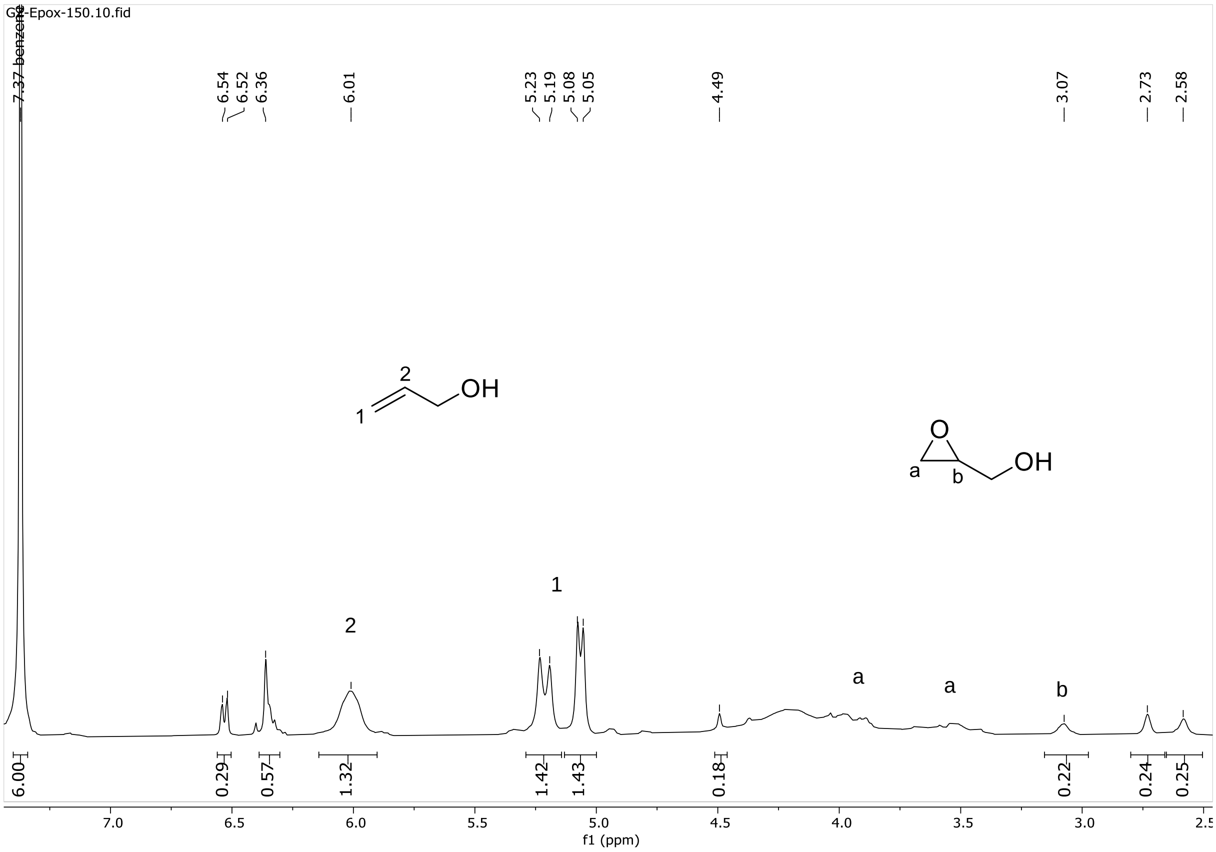

Figure 38 SI. Section of the ^1H NMR spectrum of the epoxidation of allyl alcohol (1.00 eq., 67.3 µmol/mL) with H_2O_2 (50% aq., 1.50 eq., 101 µmol/mL), catalyst **1** (1.35 µmol/mL, 0.02 eq.) and $Sc(OTf)_3$ as additive (0.10 eq., 8.41 µmol/mL) at 20 °C in CD_3CN with a reaction time of 30 min. Estimation regarding the formation of diverse side products: diol (2.50 – 2.80 ppm), acrolein (6.25 – 6.58, 9.55 ppm).

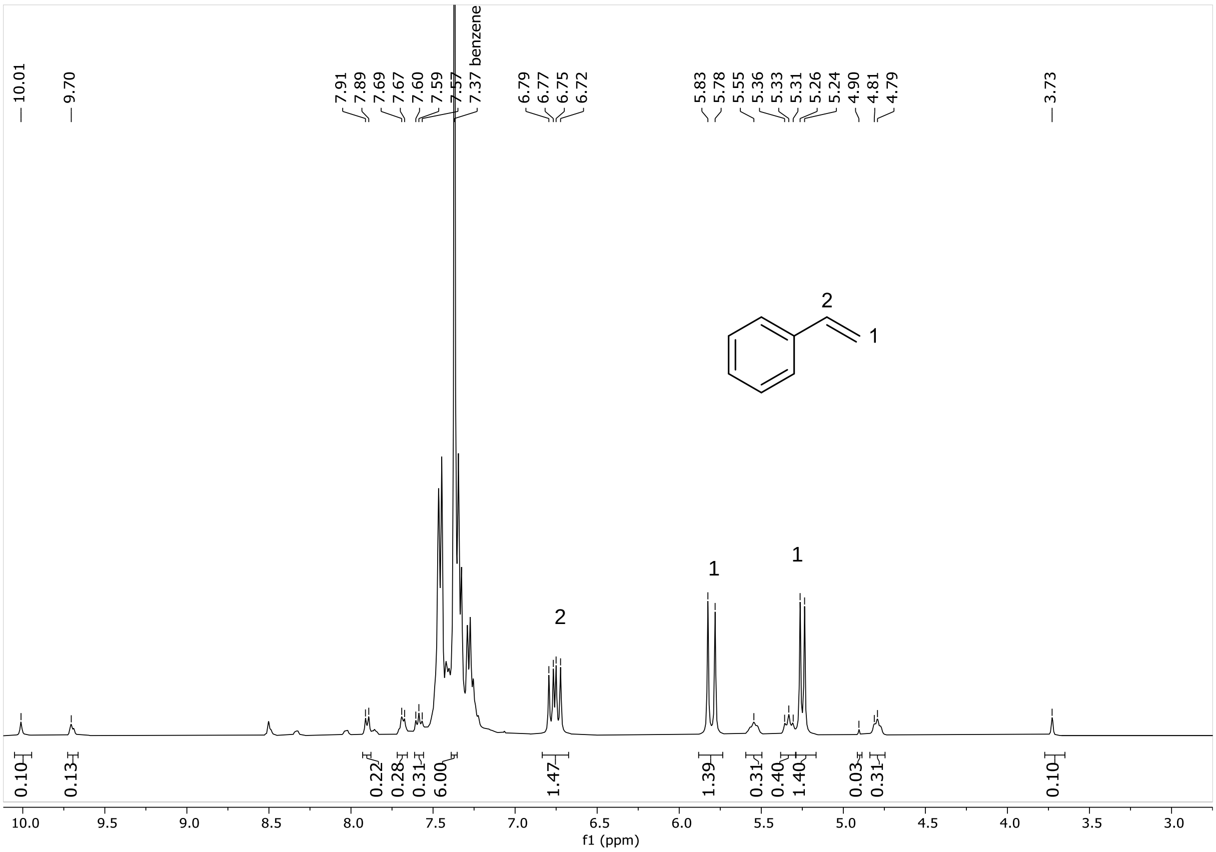

Figure 39 SI. Section of the ^{1}H NMR spectrum of the epoxidation of styrene (1.00 eq., 67.3 µmol/mL) with H_2O_2 (50% aq., 1.50 eq., 101 µmol/mL), catalyst **1** (1.35 µmol/mL, 0.02 eq.) and $Sc(OTf)_3$ as additive (0.10 eq., 8.41 µmol/mL) at 20 °C in CD_3CN with a reaction time of 30 min. No epoxide formation, estimation regarding the formation of diverse side products: phenethyl alcohol (3.73, 4.50 – 5.55 ppm), phenylacetaldehyde (9.70 ppm), benzaldehyde (10.01 ppm).

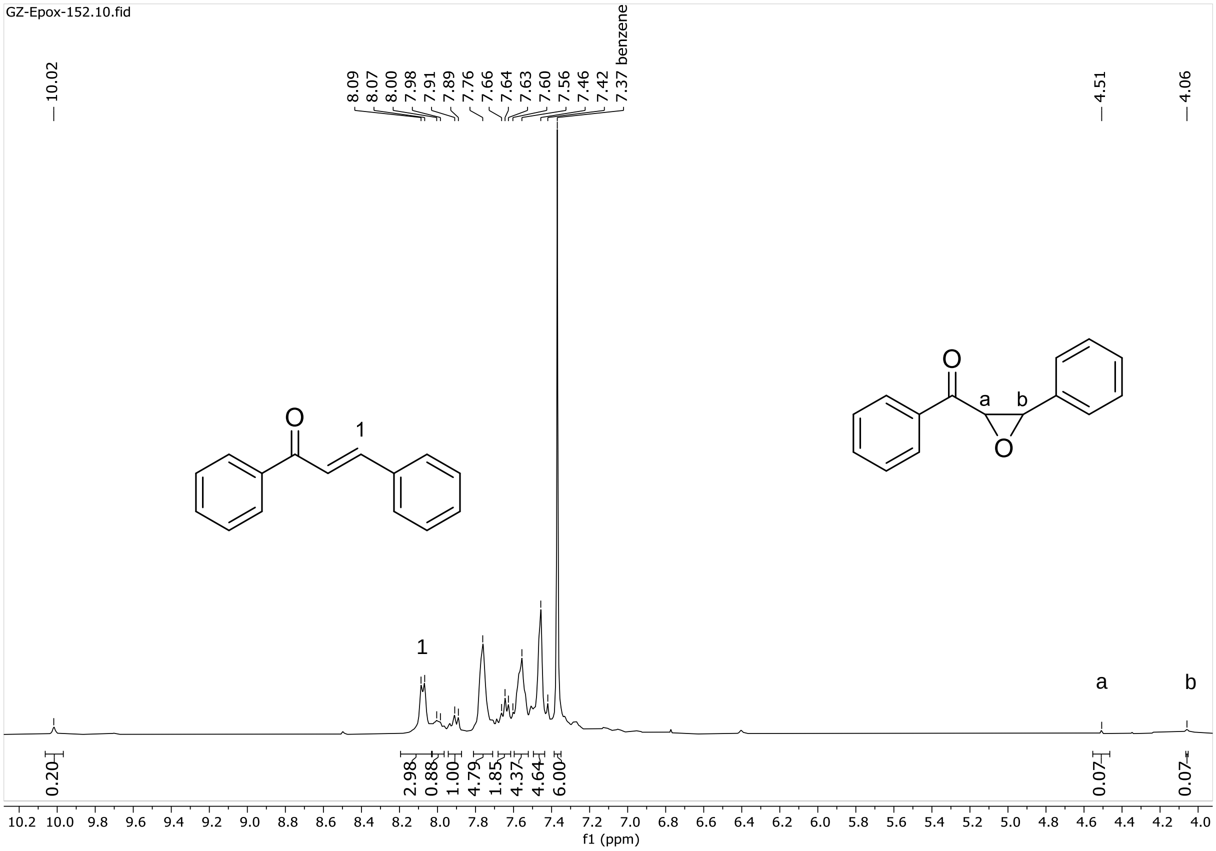

Figure 40 SI. Section of the ^{1}H NMR spectrum of the epoxidation of chalcone (1.00 eq., 67.3 µmol/mL) with H_2O_2 (50% aq., 1.50 eq., 101 µmol/mL), catalyst **1** (1.35 µmol/mL, 0.02 eq.) and $Sc(OTf)_3$ as additive (0.10 eq., 8.41 µmol/mL) at 20 °C in CD_3CN with a reaction time of 30 min. Estimation regarding the formation of the side product: benzaldehyde (10.02 ppm).

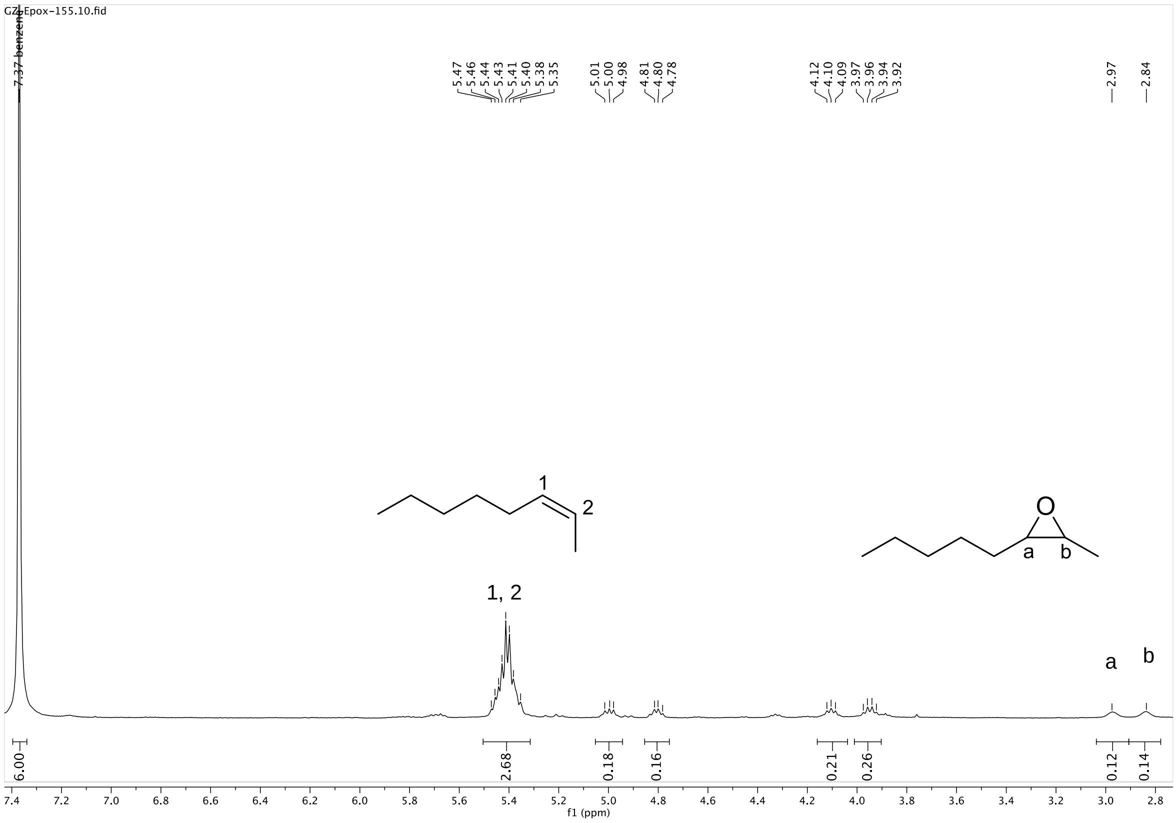

Figure 41 SI. Section of the ^{1}H NMR spectrum of the epoxidation of *cis*-2-octene (1.00 eq., 67.3 µmol/mL) with H_2O_2 (50% aq., 1.50 eq., 101 µmol/mL), catalyst **1** (1.35 µmol/mL, 0.02 eq.) and $Sc(OTf)_3$ as additive (0.10 eq., 8.41 µmol/mL) at 20 °C in CD_3CN with a reaction time of 5 min. Estimation regarding the formation of diverse side products: diols (3.40 – 4.20, 4.70 – 5.10 ppm) and aldehyde (9.69 ppm).

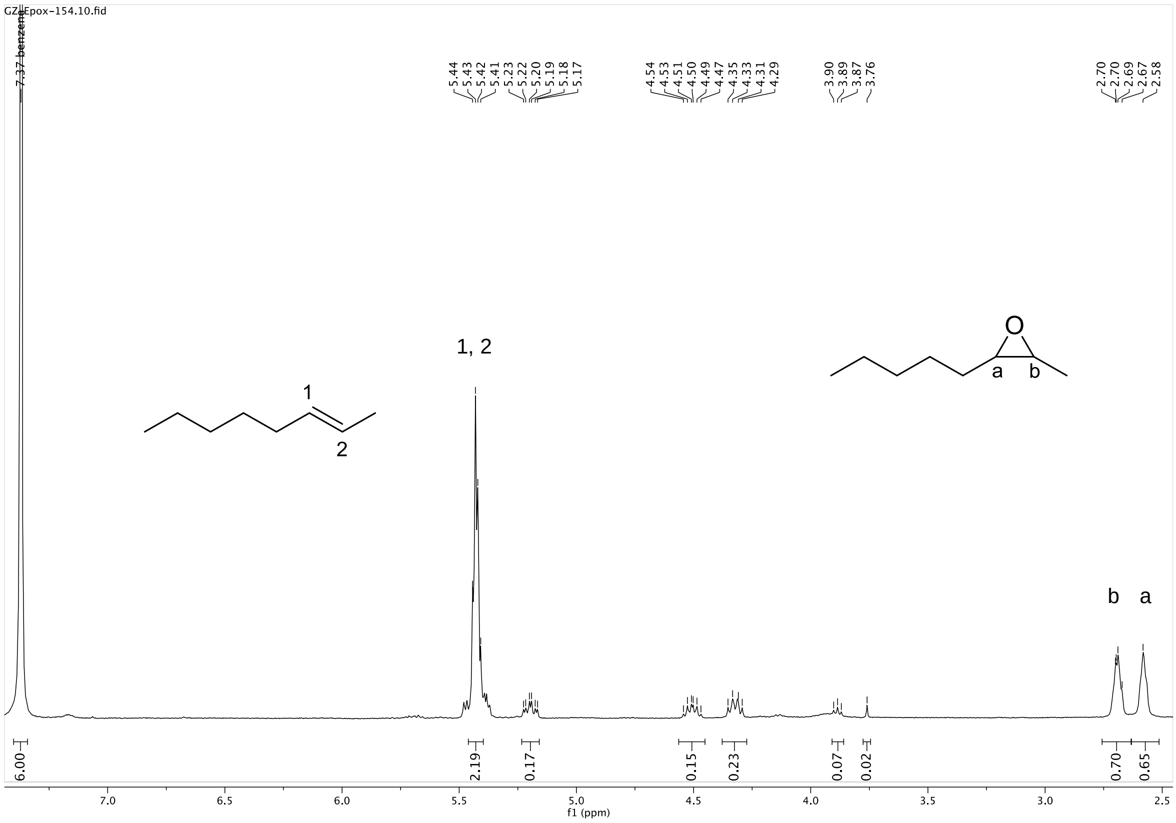

Figure 42 SI. Section of the ^{1}H NMR spectrum of the epoxidation of *trans*-2-octene (1.00 eq., 67.3 µmol/mL) with H_2O_2 (50% aq., 1.50 eq., 101 µmol/mL), catalyst **1** (1.35 µmol/mL, 0.02 eq.) and $Sc(OTf)_3$ as additive (0.10 eq., 8.41 µmol/mL) at 20 °C in CD_3CN with a reaction time of 5 min. Estimation regarding the formation of diverse side products: diols (3.80 – 4.70 ppm) and aldehyde (9.69 ppm).

9. Additional analytical data on the synthesis of iron(II) complex 1

Synthetic approaches, if not stated otherwise without analytical data indicating iron complex formation:

- $FeBr_2(THF)_2$ / NaH / r.t. (MeCN): mainly unreacted ligand precursor, small signal at m/z 269.58 potentially related to $[FeL1(MeCN)_2]^{2+}$ calcd., 270.07

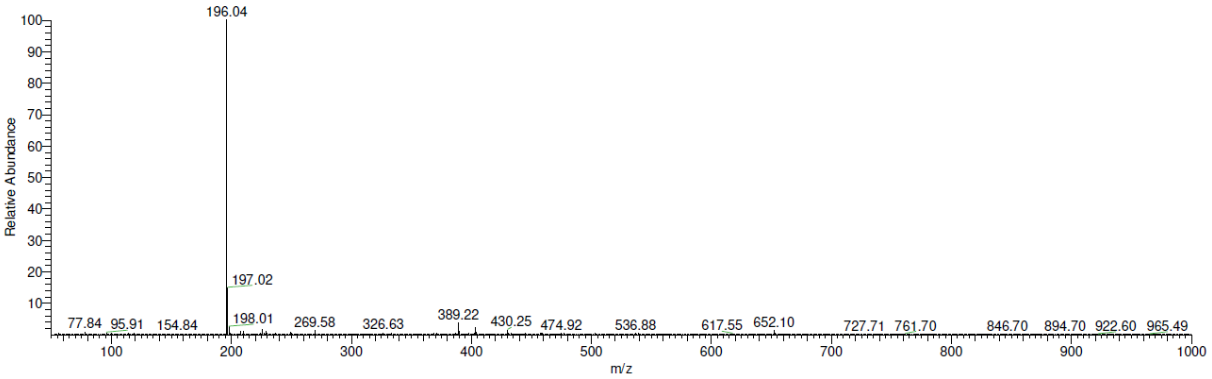

- $Fe[N(SiMe_3)_2]_2$ / $KN(SiMe_3)_2$ / -40 to 95 °C (MeCN)
- $Fe(OAc)_2$ / *n*-BuLi / -40 °C to r.t. (THF)

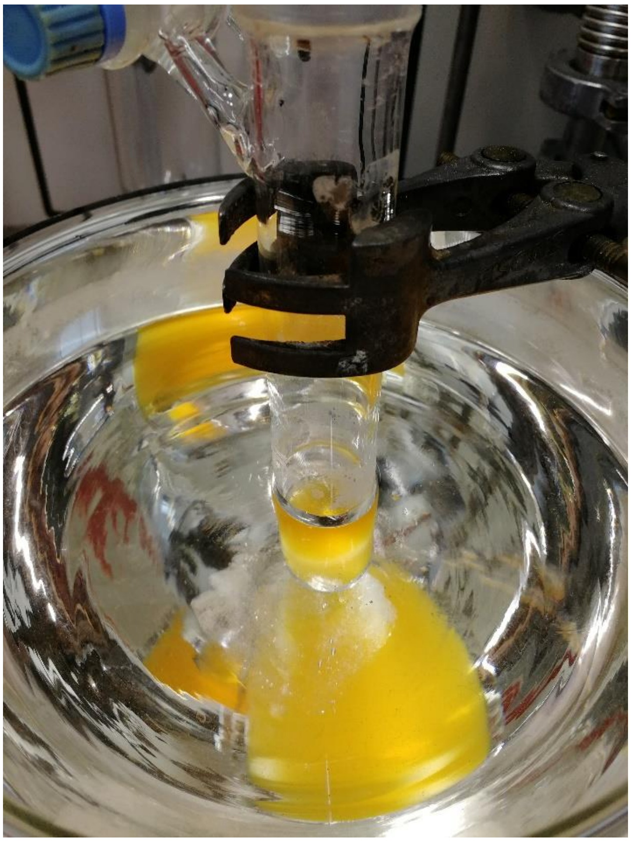

After addition of *n*-BuLi to the ligand precursor: presumably formation of the free carbene due to the intensive yellow color.

- $FeBr_2(THF)_2$ / *n*-BuLi / -78 °C to r.t. (THF)
- $FeBr_2(THF)_2$ / LiN^iPr_2 / -41 °C to r.t. (THF): mainly unreacted ligand precursor, small signals at m/z 228.86 and 269.58 potentially related to $[FeL1]^{2+}$ calcd., 229.04 and $[FeL1(MeCN)_2]^{2+}$ calcd., 270.07

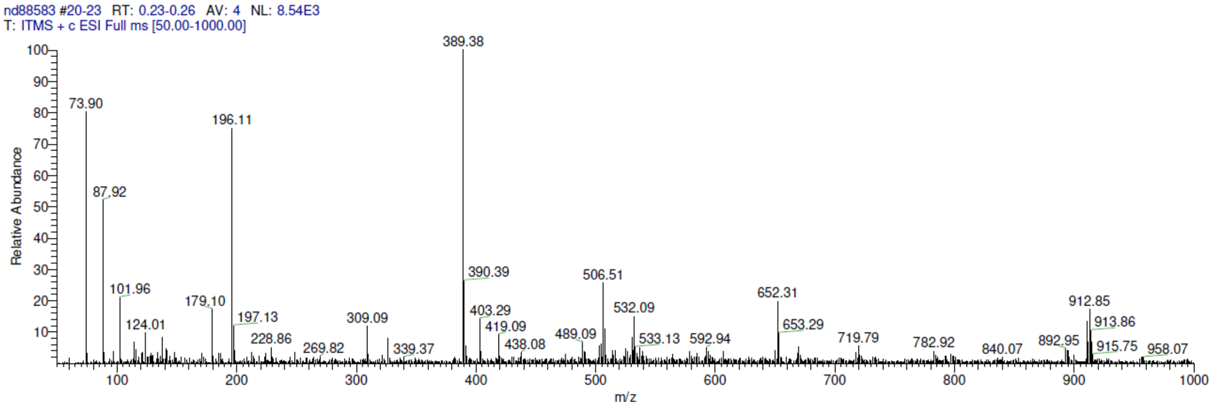

- $[H_2L1](Br)_2$ / $Fe[N(SiMe_3)_2]_2$ / -40 °C to r.t. (MeCN): m/z pattern of **1** visible ($[FeL1(MeCN)_2]^{2+}$; $[FeL1(MeCN)]^{2+}$; $[FeL1]^{2+}$) but also other impurities

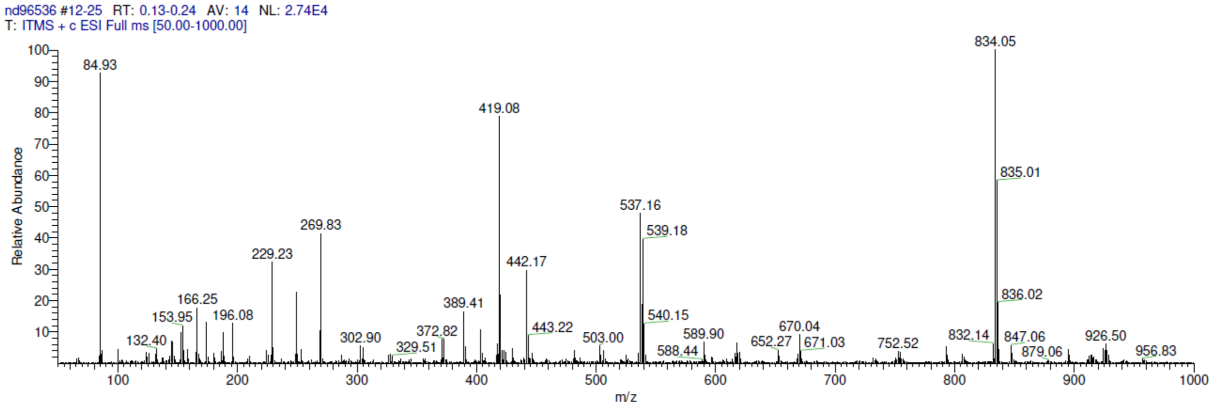

- Ag_2O / $FeBr_2(THF)_2$ / r.t., dark (MeCN)

Method B: Removal of unreacted ligand (m/z calcd. 196.09) (not as many fractions as described in the Experimental as it is from a different batch)

ESI-MS of crude product

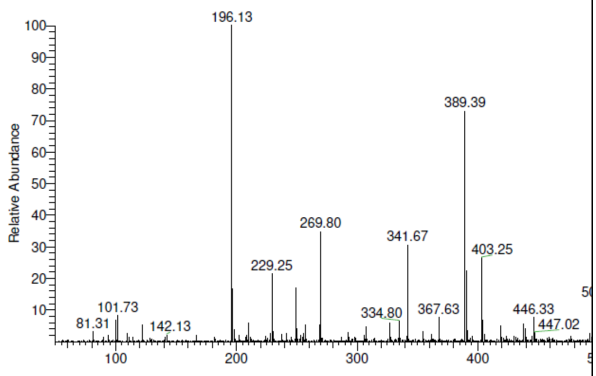

ESI-MS of supernatant 1

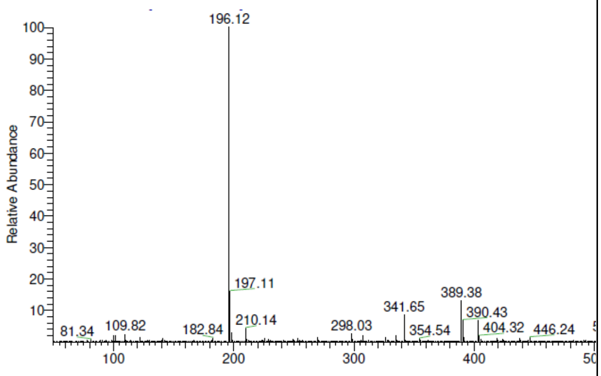

ESI-MS of supernatant 2

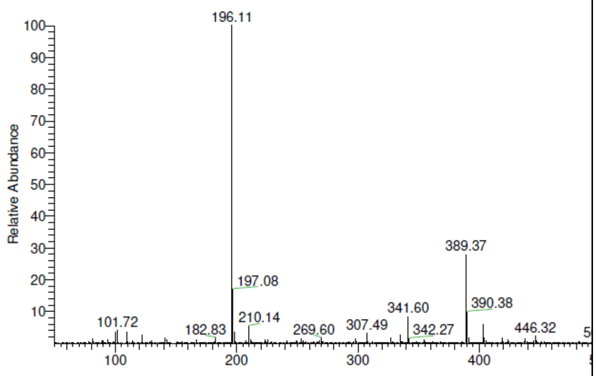

ESI-MS of supernatant 3

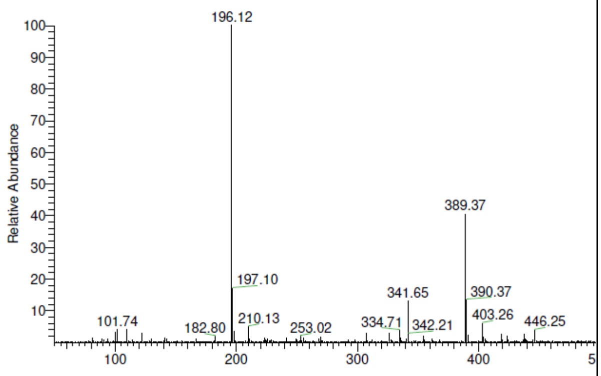

ESI-MS of supernatant 4

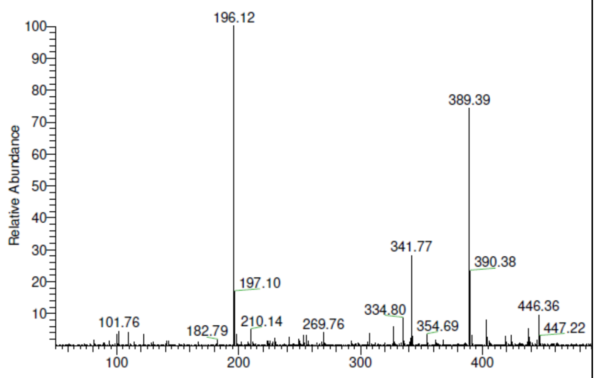

ESI-MS of supernatant 5

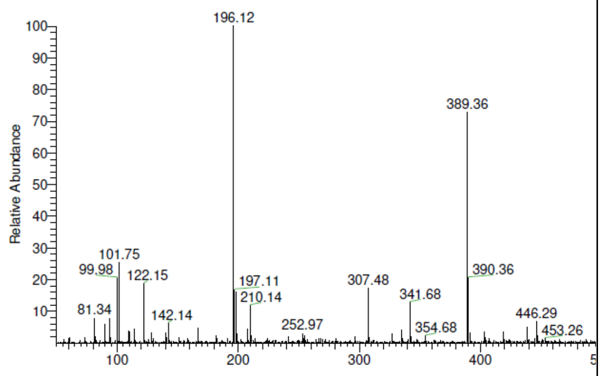

ESI-MS of supernatant 6

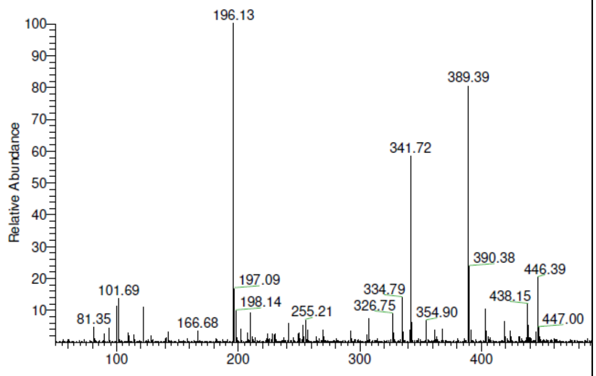

ESI-MS of supernatant 7

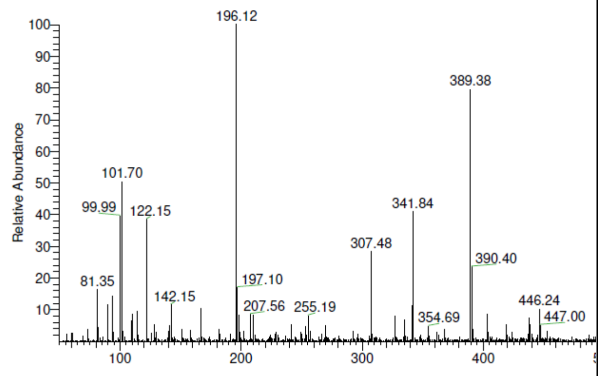

ESI-MS of supernatant 8

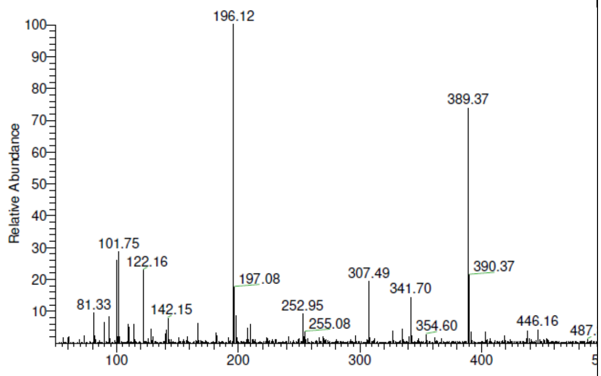

ESI-MS of supernatant 9

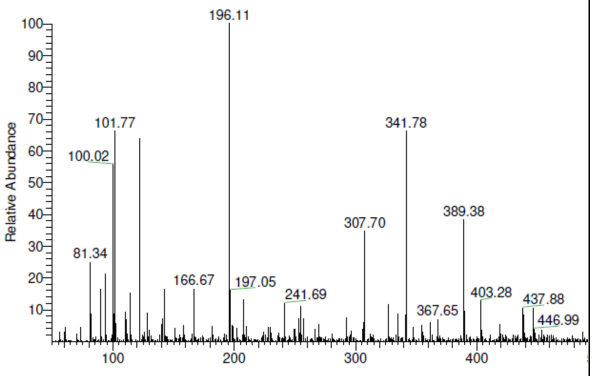

ESI-MS of supernatant 10

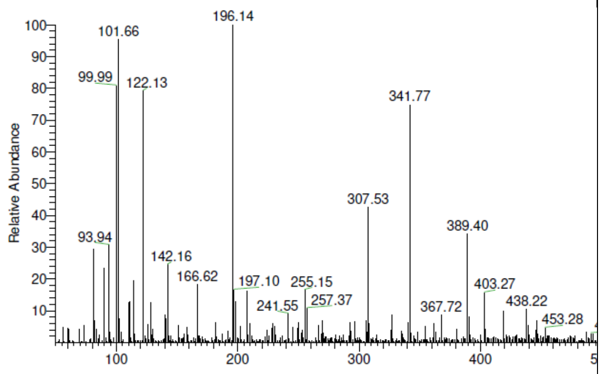

ESI-MS of supernatant 11

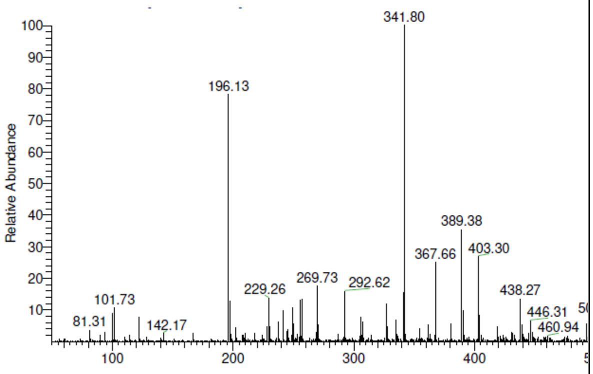

ESI-MS of supernatant 12

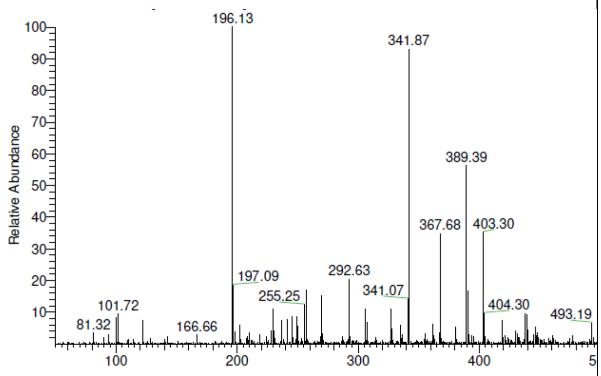

ESI-MS of supernatant 13

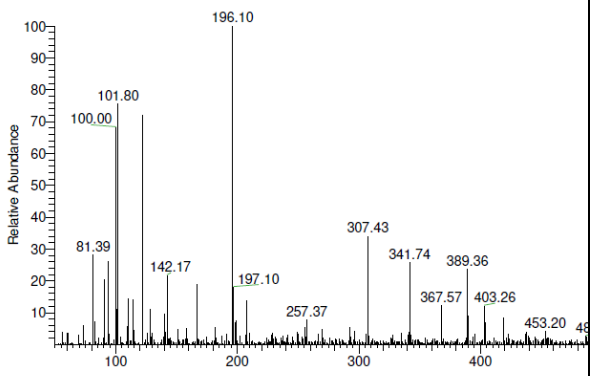

ESI-MS of supernatant 14

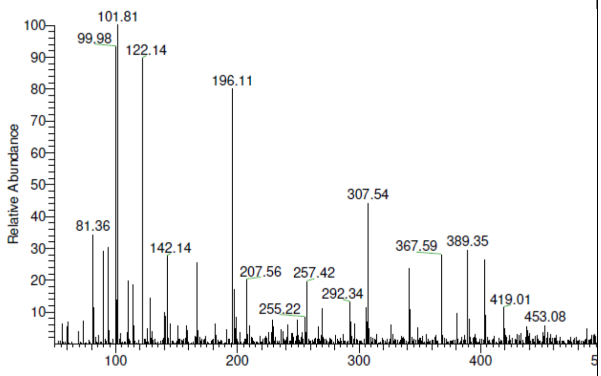

ESI-MS of supernatant 15

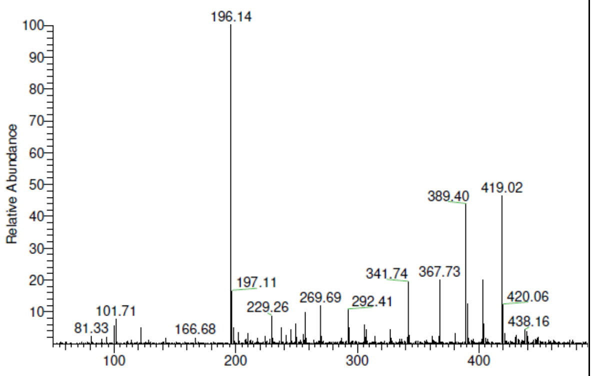

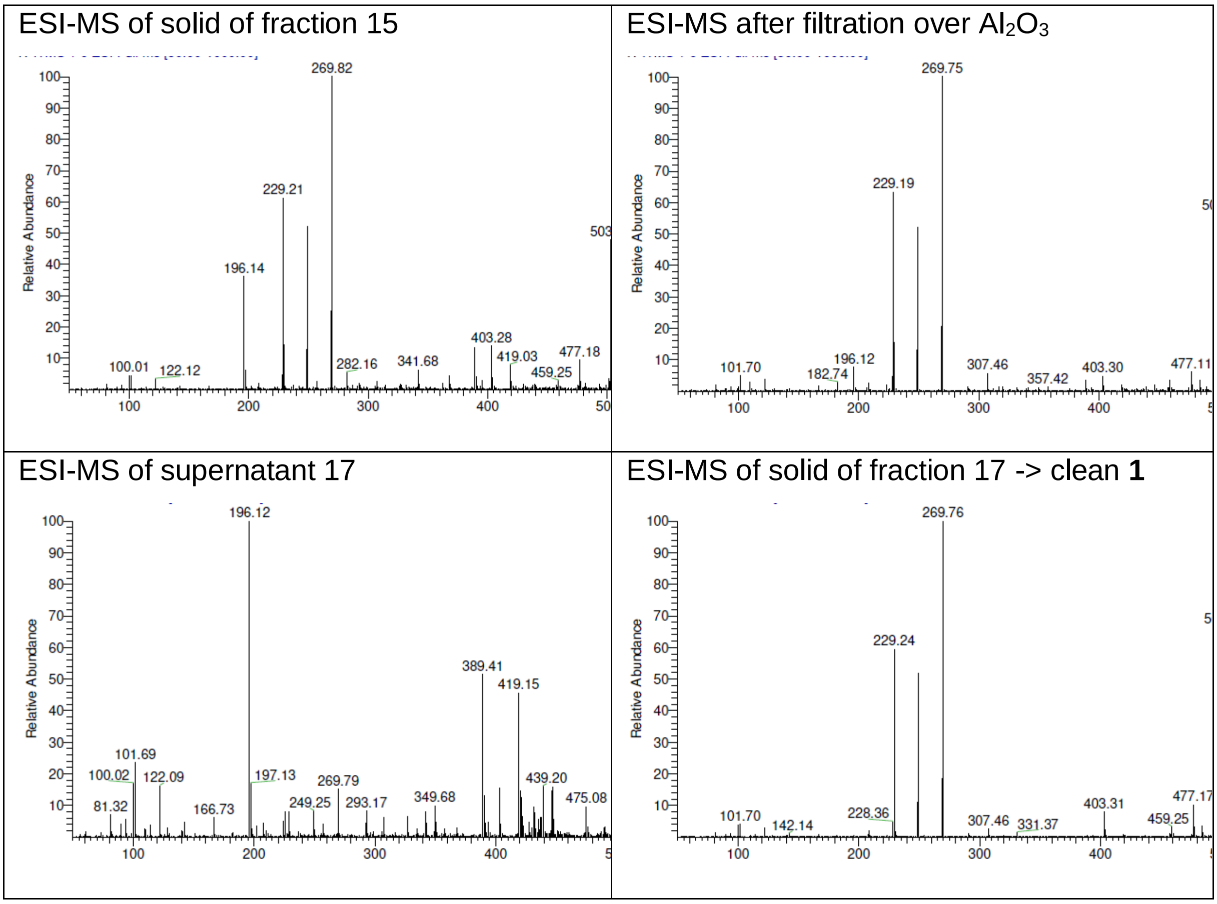

Color gradient of the different supernatants/ washing fractions (left: first fraction to right: last fraction):

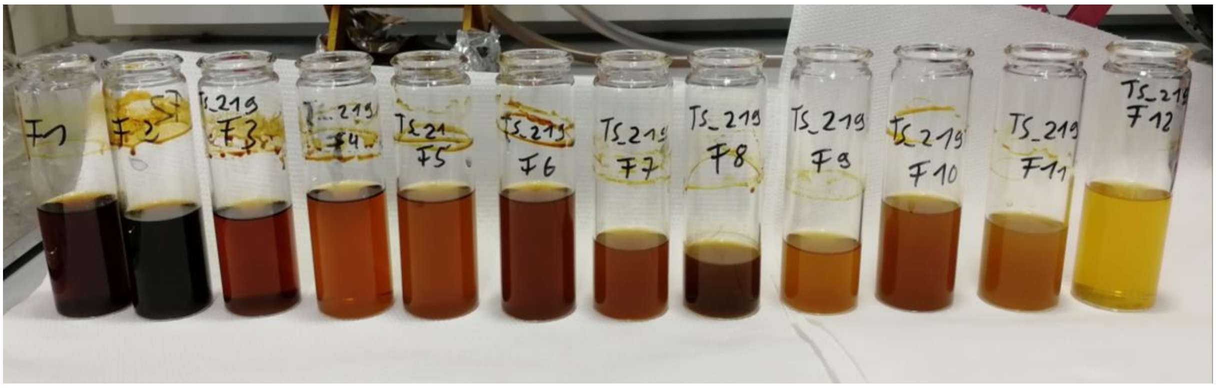

Method B: Before removal of inorganic impurity by filtration over neutral Al_2O_3

^1H NMR in CD_3CN:

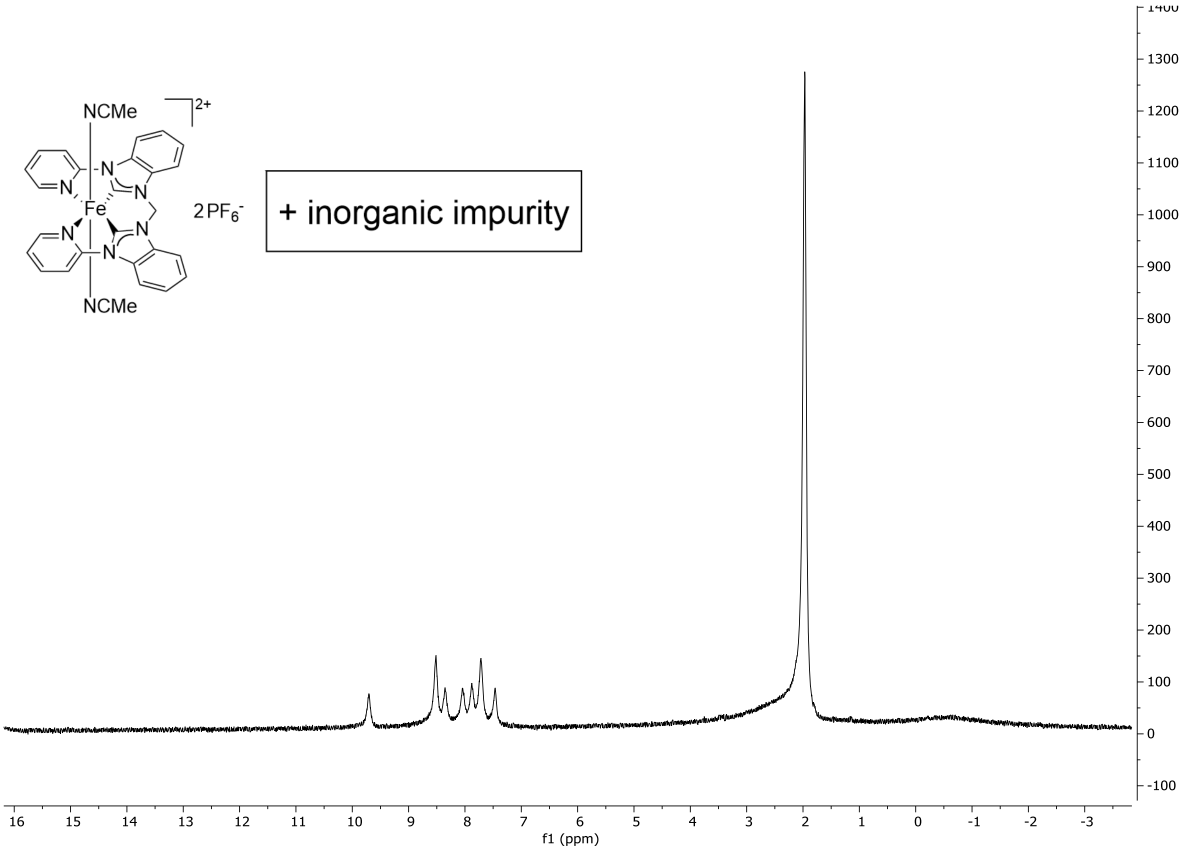

After removal see Figure 4 SI.

A + B: Filtration over neutral Al_2O_3

C: Finished filtration. Inorganic impurity is remaining on the top of the column.

D: Inorganic impurity collected as brown solid.

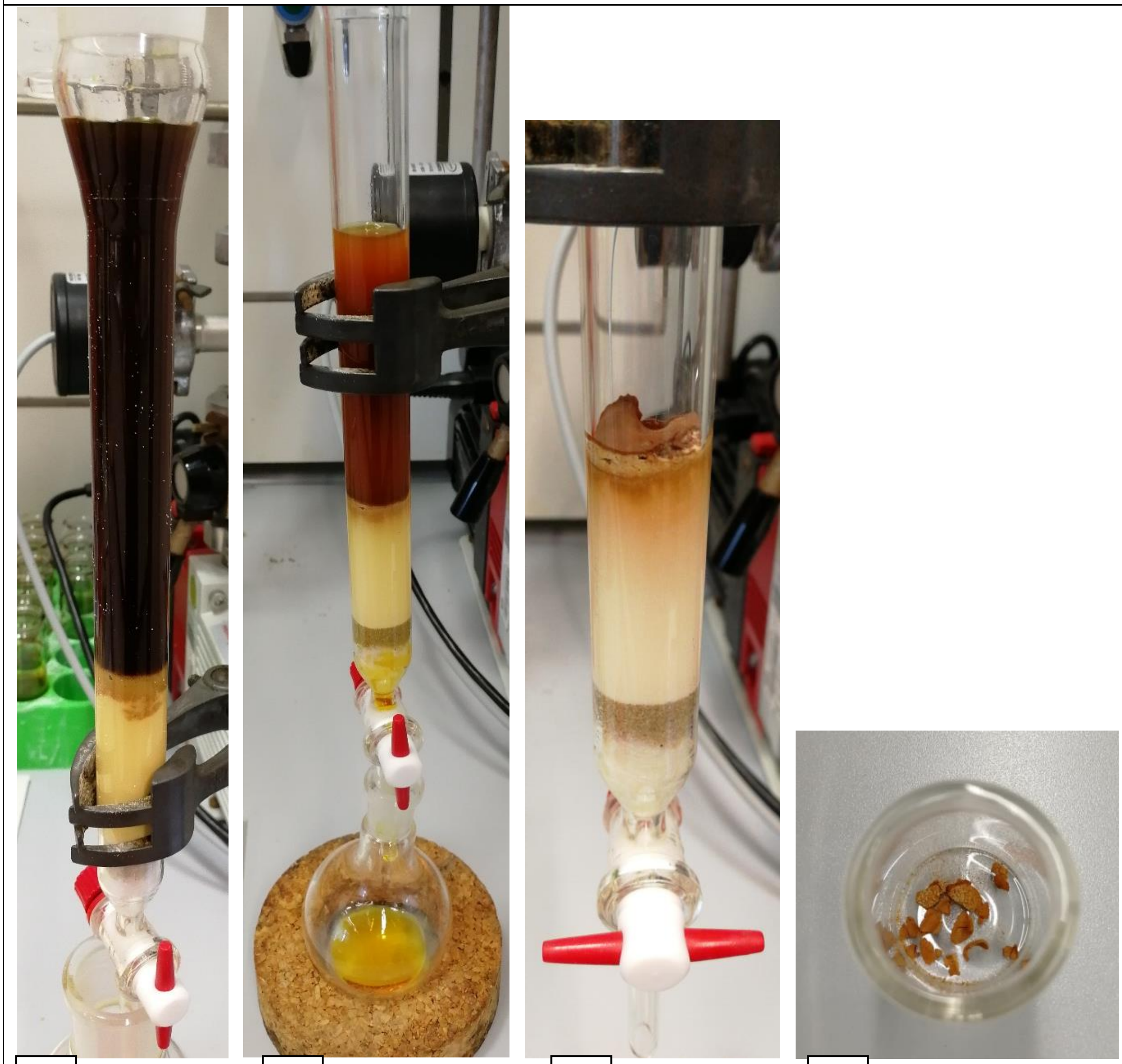

A B C D

Analytically pure iron(II) complex **1**:

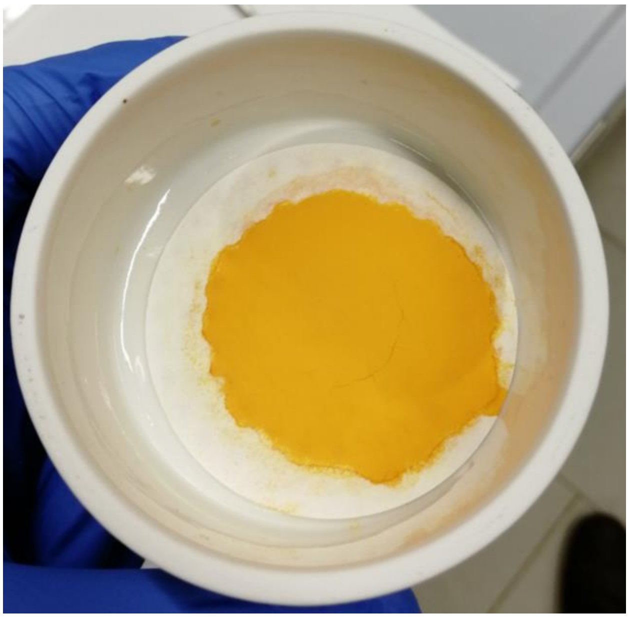

A brown precipitate has formed at the bottom of the black solution after reaction of Fe[N(SiMe$_3$)$_2$]$_2$(THF) with **[H$_2$L1](PF$_6$)$_2$** for 3 d at r.t. in MeCN, before purification as described in the Experimental of the main text.

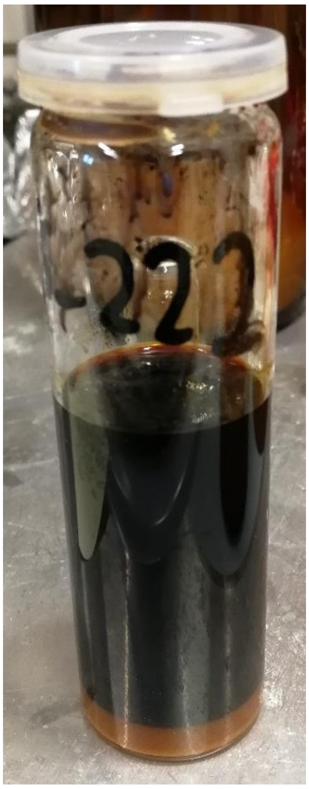
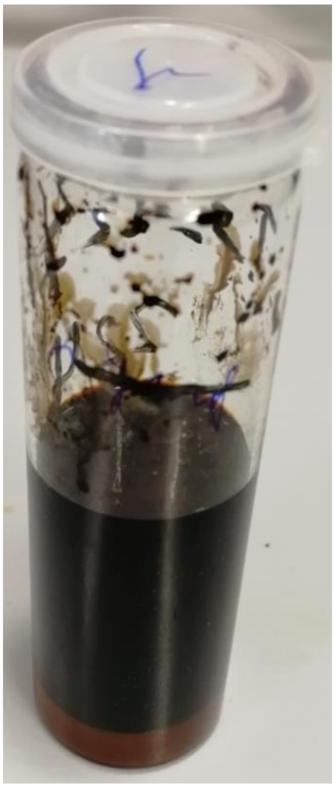

10. Synthetic attempts: saturated ligand precursor

In the following, the synthetic attempts to obtain the saturated ligand precursor (Scheme 1 SI) together with the corresponding analytical data are listed. All reactions were performed in normal atmosphere without dried and degassed chemicals. The degradation mechanism with the respective m/z values is shown in Scheme 2 SI. This chapter is based on the master's thesis of *T.P.S.*[1]

The synthetic approach is similar to the standard synthesis route used for other open-chain iron complexes (**Scheme 1** SI). The first way involves the synthesis of N^1-(pyridin-2-yl)ethane-1,2-diamine[15] and subsequent ring closure to form the imidazoline ring. From 2-(2-imidazolin-1-yl)pyridine, the ligand precursor should have been obtained by reaction with dibromomethane and anion exchange with NH_4PF_6. However, when the ring closure was performed with *N*,*N*-dimethylformamide dimethyl acetal or triethoxymethane, an inseparable mixture of 2-(2-imidazolin-1-yl)pyridine and a side product were obtained. Based on NMR and ESI-MS, the side product is assumed to be the formylated compound *N*-(2-aminoethyl)-*N*-(pyridin-2-yl)formamide (see below). Both in the ring closure employed electrophiles are usually used for the ring formation of 1,2-diamines, but they are also applied in formylation reactions.[16-17] Furthermore, the ratio of the side product increased during workup under basic conditions, indicating hydrolysis of the imidazoline ring resulting in the formylated compound. 2-Imidazolines are known to be hydrolyzed under acidic and basic conditions forming – in the case of alkaline hydrolysis – an amide and in the end the diamine and formic acid, especially when the C2 carbon atom is unsubstituted.[18-20] Substituents with an +I effect such as tertiary, secondary or long primary alkyl groups can increase the resistance of 2-imidazolines against hydrolysis at the C2 carbon atom.[1, 19-20] In addition, ring cleavage was also observed in literature for a similar compound, 2-((2-imidazolin-1-yl)methyl)pyridine.[20] Direct synthesis of 2-imidazoline with 2-chloropyridine leads to the same result as described above. Apparently inseparable mixtures of product and side product are obtained after column chromatography (see below). These observations indicate two possible explanations: A) formylation occurs as a side reaction. However, an intermediate, which is formed first by formylation of secondary amines with *N*,*N*-dimethylformamide dimethyl acetal, namely *N*-formyl-*N,N*-dialkylamine, could not be observed.[1, 16] Also, only one spot was visible in TLC and the formylated side product should in theory be separable in column chromatography. B) Hydrolysis of the imidazoline ring takes place. This would explain the presence of impurities even after column chromatography but also indicate a low stability of the desired product. Both mechanisms are given in **Scheme 2** SI.

Further reaction of 2-(2-imidazolin-1-yl)pyridine - however only impure - with CH_2Br_2 was not successful. In a last approach, the *N*-oxide of 2-bromopyridine was used to potentially increase the selectivity of the reaction by protection of the nucleophilic nitrogen atom and deprotection

before complexation of the final ligand precursor.[21-32] Indeed, the synthesis of 2-(2-imidazolin-1-yl)pyridine 1-oxide was successful without the observation of degradation products, but first coupling reactions with CH_2Br_2 were not promising.

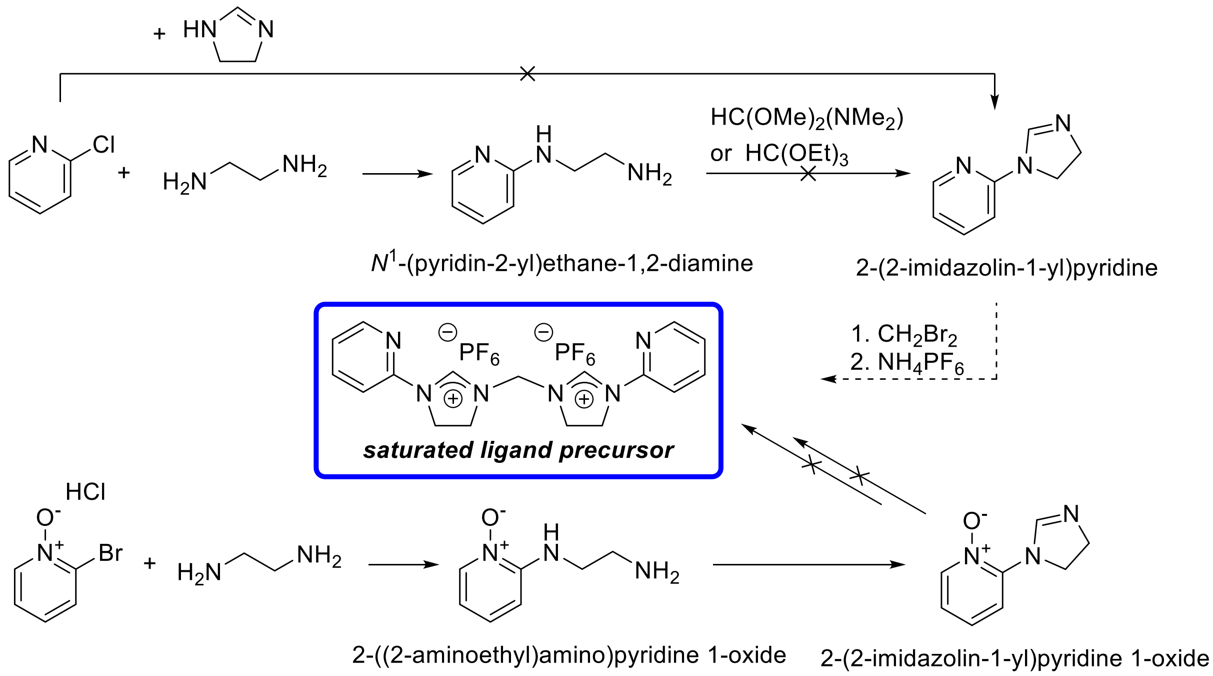

Scheme 1 SI. Planned synthetic route to the saturated ligand precursor.

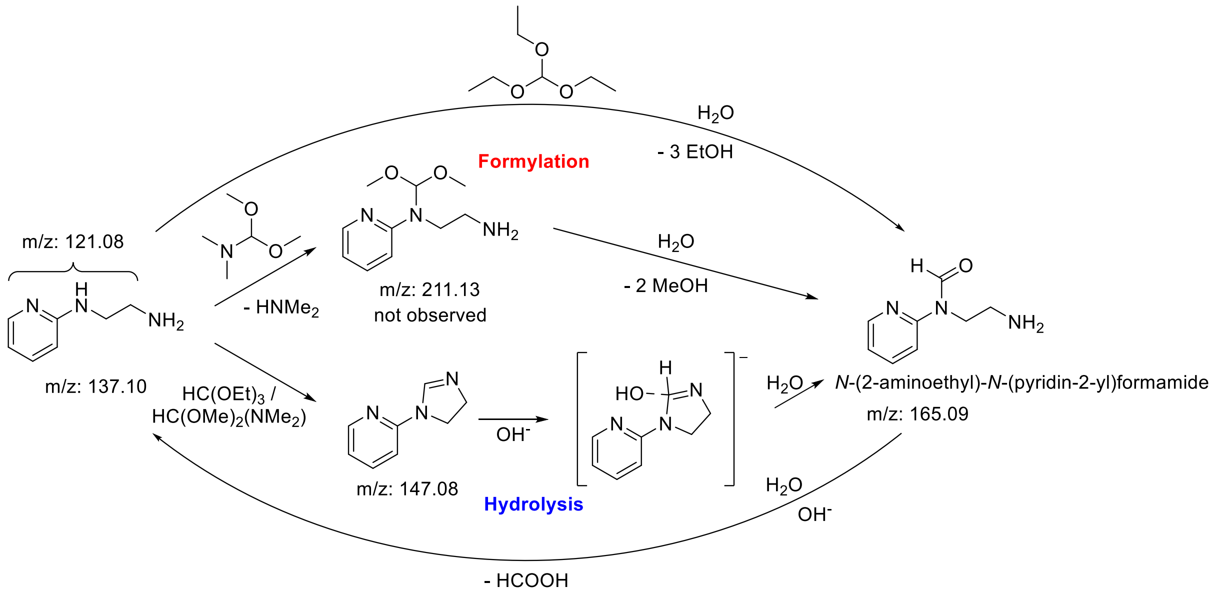

Scheme 2 SI. Proposed formylation and hydrolysis mechanisms. Note, that the displayed structures will be positively ionized in ESI-MS, increasing the m/z by 1 in the case of H^+.

Note, that the ionized m/z value of the postulated formylated side product (*i.e.* [M + H^+] calcd. 166.10) is also matched by the mass of the desired product, 2-(2-imidazolin-1-yl)pyridine, flying with a H_3O^+ molecule ([M + H_3O^+] calcd. 166.10). However, this m/z value is attributed to the proposed formylated side-product due to two reasons: a) if this value is caused by 2-(2-imidazolin-1-yl)pyridine flying with H_3O^+ in the mass spectrum, the ratio between this fragment and

the product alone ([M + H^+] calcd. 148.09) should remain somewhat similar in each mass spectrum. This is, however, not the case. For example, in Figure 52 SI, mainly the signal of 2-(2-imidazolin-1-yl)pyridine is present. b) Due to the presence of (broad) alien proton signals in otherwise purified samples (*i.e.* containing both m/z 148.09 and 166.10 signals), attributable to amine or formaldehyde groups, indicating towards a formylated side product. Furthermore, as described in the main text, both electrophiles are also known for formylation reactions.[16-17] And in addition, the formylated side product can also be formed by hydrolysis, as observed after aqueous alkaline purification with the rise of the 166.10 fragment.

For 2-(2-imidazolin-1-yl)pyridine 1-oxide, it is the other way around: The fragment at 181.94 m/z (Figure 14 SI) is assigned to [M + H_3O^+] (calcd., 182.09) due to the coherent NMR spectrum (Figure 8 SI). A potentially formylated product is thereby excluded ([theoretical mass formylated product + H^+] calcd., 182.09).

New synthetic procedures:

2-Imidazoline.[1] The synthesis of 2-imidazoline is based on the formation of the larger six membered ring 1,4,5,6-tetrahydropyrimidine.[33-34] *N*,*N*-dimethylformamide dimethyl acetal (40.0 g, 44.7 mL, 336 mmol, 1.0 eq.) is added to ethylenediamine (20.2 g, 22.4 mL, 336 mmol, 1.0 eq.) and the mixture is stirred at 110 °C for 1 d. All volatiles are removed *in vacuo* resulting in a light yellow wax. A part of the raw product is sublimed from 50 °C to 90 °C at around 10^{-2} mbar to form colorless crystals. They are removed from the cooling finger in inert atmosphere to obtain 2-imidazoline as a very hygroscopic white solid (7.19 g, 103 mmol, 54% total yield based on the amount of received purified product). 2-Imidazoline forms into a glassy wax/ solid in contact to air moisture. ^{1}H NMR analysis still shows the presence of an impurity, which could be assigned to dimethylformamide. With elemental analysis, the amount of dimethylformamide in the product is calculated to be around 3%. The amine proton signal is barely visible in the ^{1}H NMR spectrum at 7.44 ppm. The residual signals are remaining impurities after sublimation, assigned to DMF based on the signal at 7.98 ppm (C*H*)[35] and because DMF appears to be a usual byproduct when using *N*,*N*-dimethylformamide dimethyl acetal to form the imidazoline ring[36]. ^{1}H NMR (400.13 MHz, D_2O): δ 8.08 (s, 1H, C*H*), 7.44 (s, 1H, N*H*), 3.29 (t, 2H, NCH_2, ^3J = 6.2 Hz), 2.73 (t, 2H, NCH_2, ^3J = 6.2 Hz). ESI-MS (m/z): [M +H^+] calcd., 71.06; found, 71.75 (100); [M +H_3O^+] calcd., 89.07; found, 88.78 (14). Anal. calcd. for $C_3H_6N_2$: C 51.41; H 8.63; N 39.97. Found: C 50.59; H 8.66; N 39.08.

2-((2-Aminoethyl)amino)pyridine 1-oxide. 2-Bromopyridine 1-oxide hydrochloride (510 mg, 2.42 mmol, 1.00 eq.) is suspended in 10 mL MeCN. 5 mL ethylenediamine are added to the suspension under stirring resulting in an orange solution. Then, the mixture is stirred at 90 °C for 20 h. The solvent is removed under vacuum. 25 mL DCM and 25 mL of an aqueous saturated Na_2CO_3 solution are added to the orange oil. The organic phase is separated, and the aqueous phase is extracted with DCM (4 × 25 mL). All organic phases are collected, dried over Na_2SO_4 and filtrated to result in a yellow solution. After removing the solvent, the desired product is obtained as yellow-orange crystals (0.43 g, yield too high due to impurities). The product was used without any further purification in the next synthesis. If necessary, column chromatography can be performed (DCM/MeOH/NEt_3 20:11:1, R_f = 0.22 [UV]). The analytical data is in agreement with the literature.[31] ^{1}H NMR (400.13 MHz, $CDCl_3$): δ 8.10 (dd, ^3J = 6.5, ^4J = 1.2 Hz, 1H, H_{py}), 7.18 (ddd, ^3J = 8.6, ^3J = 7.3, ^4J = 1.2 Hz, 1H, H_{py}), 6.63 (dd, ^3J = 8.5, ^4J = 1.5 Hz, 1H, H_{py}), 6.55 (ddd, ^3J = 7.4, ^3J = 6.5, ^4J = 1.7 Hz, 1H, H_{py}), 3.34 (m, 2H, CH_2), 2.99 (dd, ^3J = 6.5, ^3J = 5.5 Hz, 2H, CH_2). ESI-MS (m/z): [M + H^+] calcd., 154.10; found, 153.92 (100); [M – O] calcd., 137.10; found, 136.91 (42); [M – O – CH_2NH_2] calcd., 107.06; found, 106.89 (65).

2-(2-Imidazolin-1-yl)pyridine 1-oxide. 2-((2-Aminoethyl)amino)pyridine 1-oxide (371 mg, 2.42 mmol, 1.00 eq.) is dissolved in 20 mL MeCN (obtained from solvent purification system, *i.e.* somewhat dry). *N*,*N*-dimethylformamide dimethyl acetal (0.40 mL, 3.00 mmol, 1.24 eq.) is added and the mixture is stirred at 95 °C for 17 h to form an orange solution. The solvent is removed under vacuum. The resulting orange solid is purified by column chromatography (55 g SiO_2, 400 mL DCM/MeOH 4:1, 600 mL DCM/MeOH 3:1) to yield 2-(2-imidazolin-1-yl)pyridine 1-oxide as yellow wax (0.12 g, 0.74 mmol, 30%). TLC: R_f = 0.21 (DCM/MeOH 4:1) [UV] ^{1}H NMR (400.13 MHz, CD_3OD): δ 8.11 (s, 1H, NC*H*N), 8.09 (dd, ^3J = 6.6, ^4J = 1.2 Hz, 1H, H_{py}), 7.45 (ddd, ^3J = 8.8, ^3J = 7.4, ^4J = 1.5 Hz, 1H, H_{py}), 7.00 (dd, ^3J = 8.7, ^4J = 1.5 Hz, 1H, H_{py}), 6.72 (ddd, ^3J = 7.3, ^3J = 6.6, ^4J = 1.6 Hz, 1H, H_{py}), 3.50 (m, 4H, CH_2). ESI-MS (m/z): [M + MeCN] calcd., 204.10; found, 203.95 (64); [M + H_3O^+] calcd., 182.09; found, 181.94 (88); [M + H^+] calcd., 164.08; found, 163.98 (100).

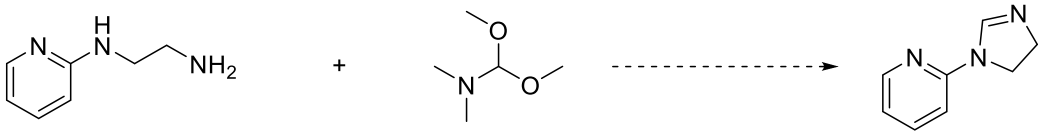

N^1-(pyridin-2-yl)ethane-1,2-diamine

N,*N*-dimethylformamide dimethyl acetal

2-(2-imidazolin-1-yl)pyridine

	Solvent	T [°C]	Reaction time [h]	Comment
**S1**	MeOH	85	14	Mixture of product, potentially formylated side product and educt (Figure 43 SI, Figure 44 SI)
**S2**	–	105	3 d	Reaction of crude product of **S1** again with *N*,*N*-dimethylformamide dimethyl acetal to ensure complete reaction<br>Product signals (Figure 45 SI, Figure 46 SI) but two alien signals in NMR, potentially from formylated side product *N*-(2-aminoethyl)-*N*-(pyridin-2-yl)formamide, with the signal of the formaldehyde group at 8.82 ppm[16]; ESI-MS also showing potentially formylated side product<br><br>To exclude, that the alien proton signals did not derive from a protonation, e.g. the imine group forming an iminium ion, a sample from the product mixture was dissolved in an alkaline aqueous solution (pH = 11 using KOH) and extracted again with DCM.<br>Now, the relative intensity of the formylated side product at 165.97 m/z is higher, than the signal of the product at 148.03 m/z (Figure 47 SI), which was the other way round in the previous mass spectrum (Figure 46 SI), implying a higher concentration of the side product, thus supporting hydrolysis as possible degradation mechanism. In NMR, still broad signals characteristic for amines are visible (Figure 48 SI)

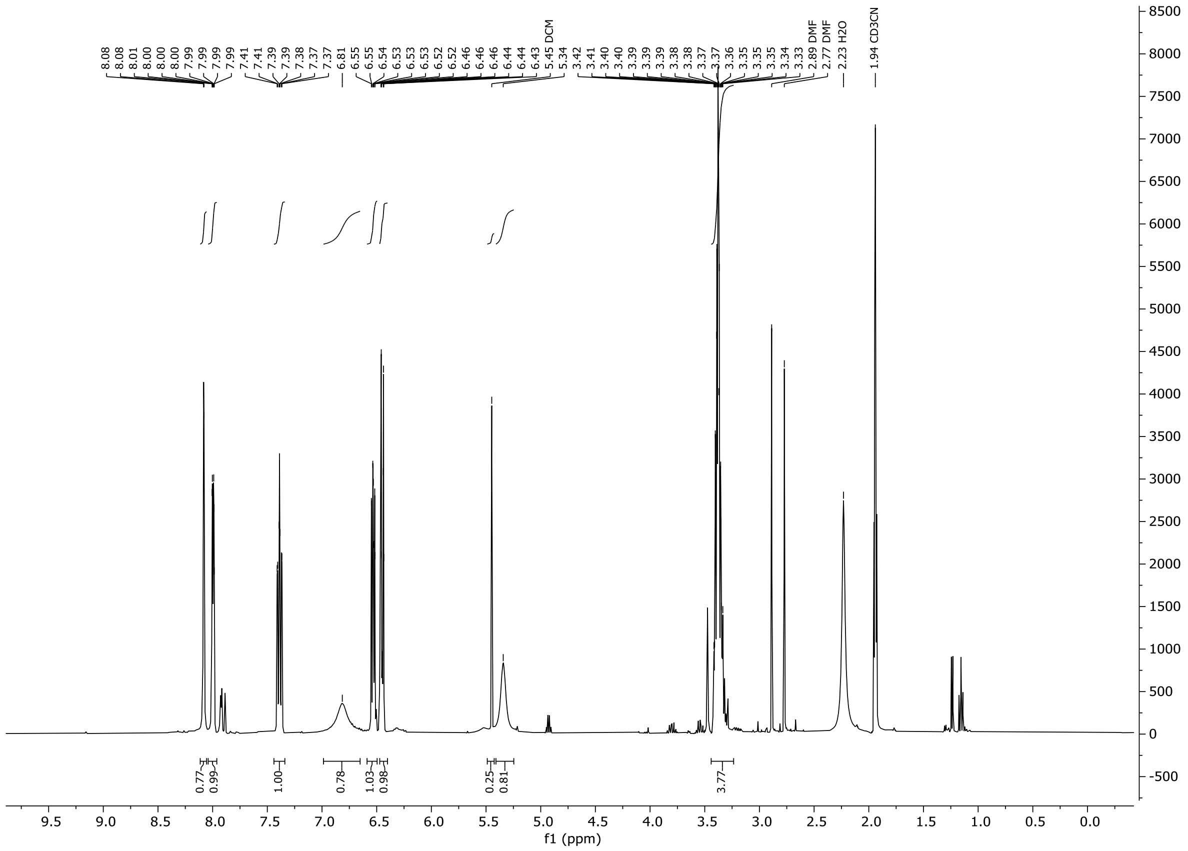

Figure 43 SI. ^{1}H NMR spectrum in CD_3CN from the crude product of **S1** after extraction.

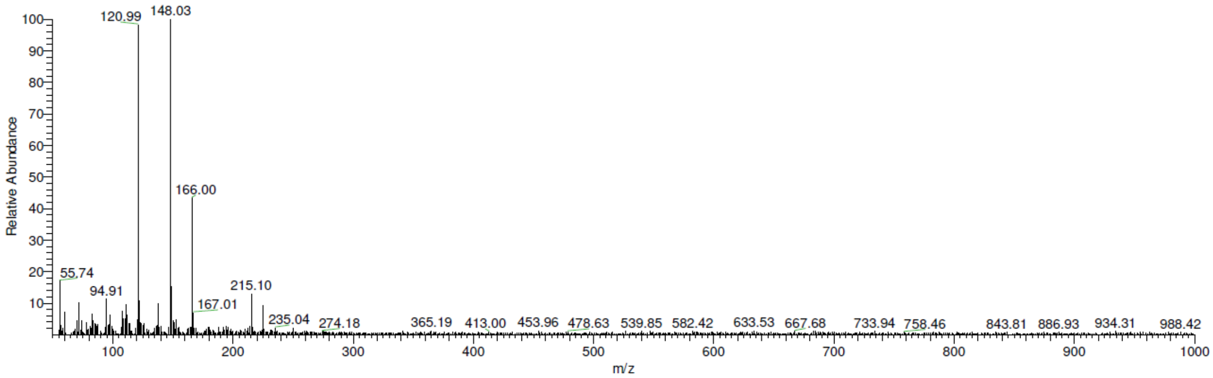

Figure 44 SI. ESI-MS spectrum from the crude product of **S1** after extraction.

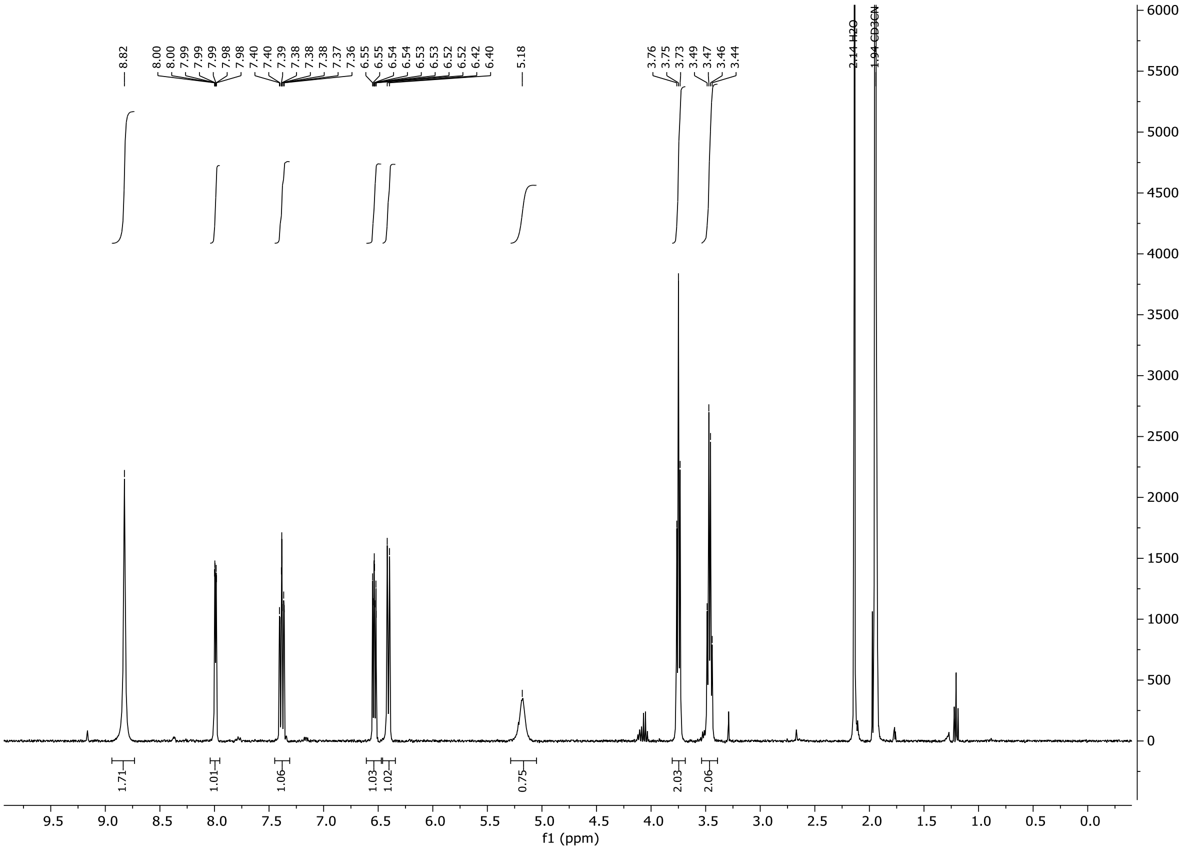

Figure 45 SI. ^{1}H NMR spectrum of **S2** in CD_3CN from the dried slightly yellow oil after column chromatography (80 – 100 % EtOAc in *n*-pentane, R_f = 0.26 for 80 % EtOAc in *n*-pentane).

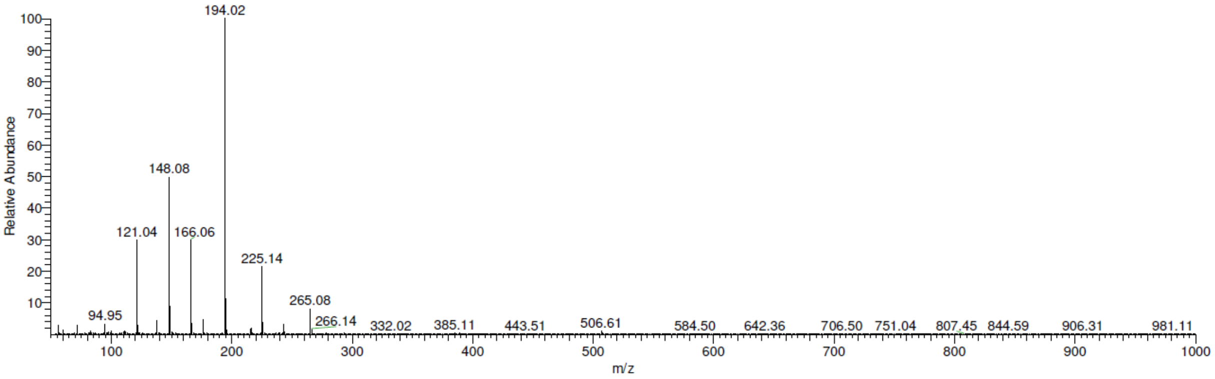

Figure 46 SI. ESI-MS spectrum of **S2** after column chromatography. The peak at 194.02 m/z can be attributed to the product 2-(2-imidazolin-1-yl)pyridine: [M +H^+ +HCOOH (from buffer)] calcd., 194.09; found, 194.02 (100).

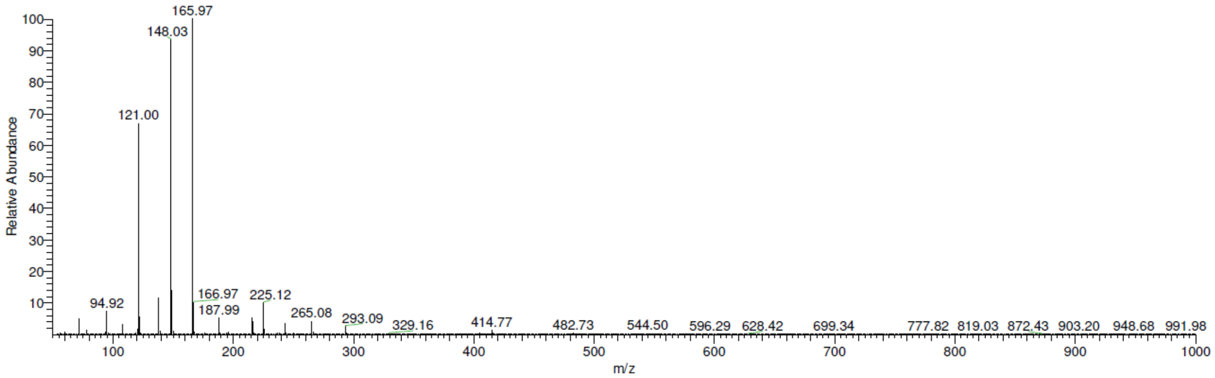

Figure 47 SI. ESI-MS spectrum of **S2** after extraction from alkaline aqueous phase.

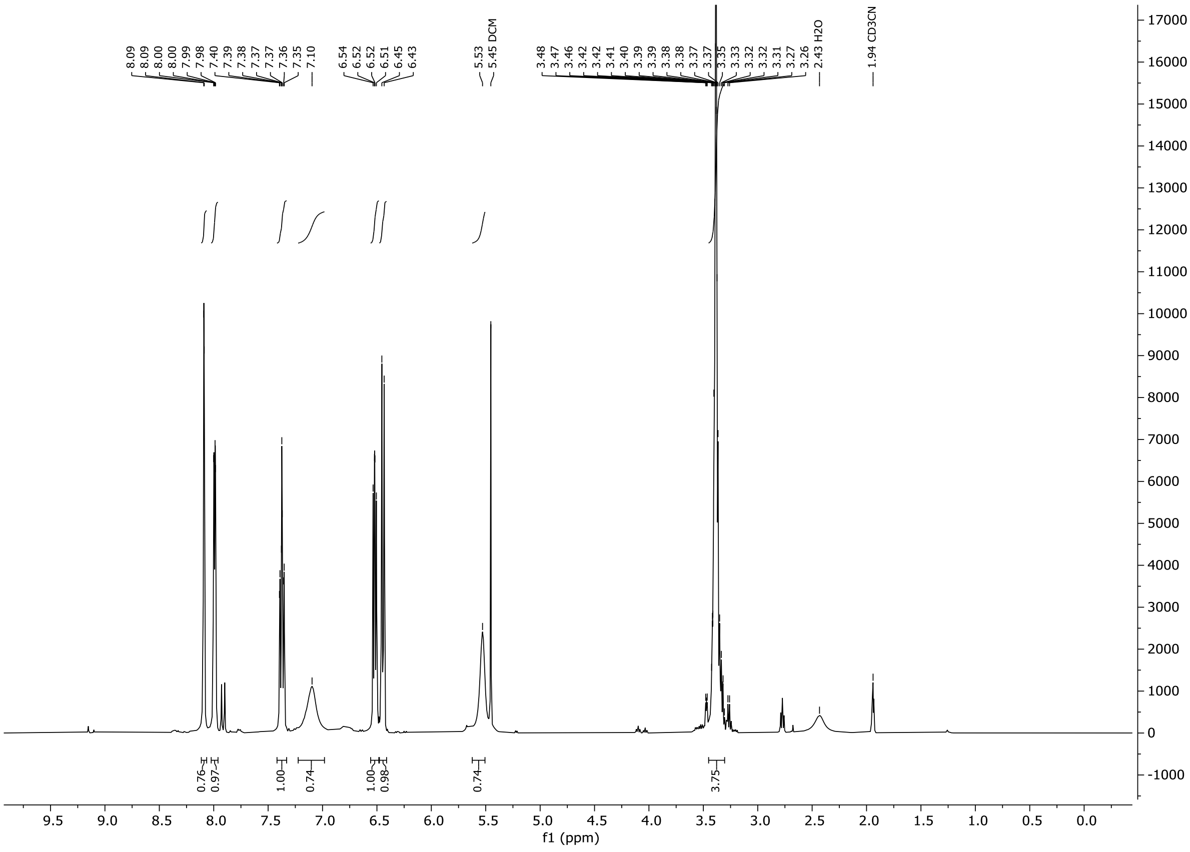

Figure 48 SI. ^{1}H NMR spectrum of **S2** in CD_3CN after extraction from alkaline aqueous phase.

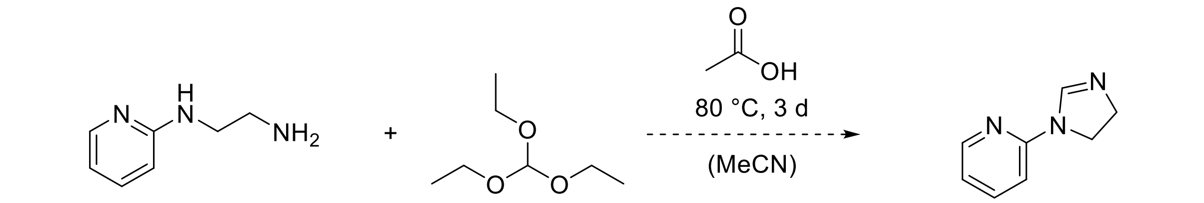

N[1]-(pyridin-2-yl)ethane-1,2-diamine triethoxymethane 2-(2-imidazolin-1-yl)pyridine

	Solvent	T [°C]	Reaction time [h]	Comment
**S3**	MeCN	80	3 d	analog to a literature procedure[37]<br>Product signals and potentially formylated side product after column chromatography (0 – 10 % MeOH in EtOAc, R_f = 0.19 for 10 % MeOH in EtOAc) (Figure 49 SI) and broad alien signals in NMR (Figure 50 SI).<br>Lee *et al.*[20] used triethoxymethane as well to synthesize a similar compound, 2-((2-imidazolin-1-yl)methyl)pyridine. However, no detailed synthesis and purification is described.<br>A sample of the product mixture was subjected to reversed phase column chromatography with capped silica and a gradient of 10 – 90 % MeCN in water. Still no separation of the compounds could be achieved (Figure 51 SI)

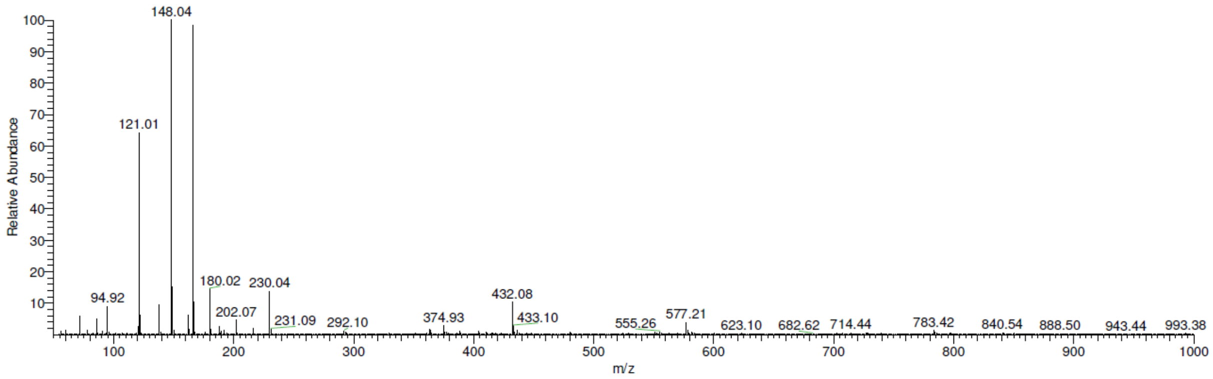

Figure 49 SI. ESI-MS spectrum of **S3** from the third fraction after column chromatography.

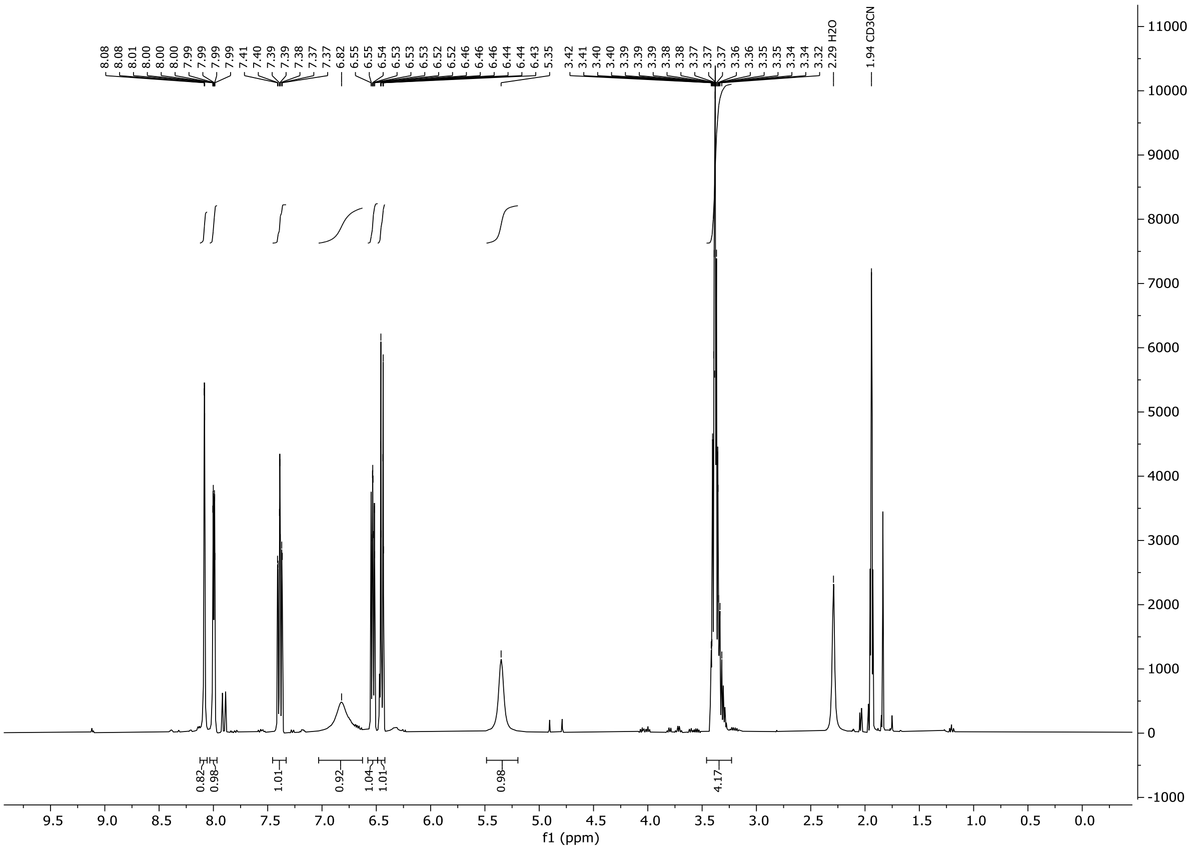

Figure 50 SI. ^{1}H NMR spectrum of **S3** in CD_3CN from the third fraction after column chromatography.

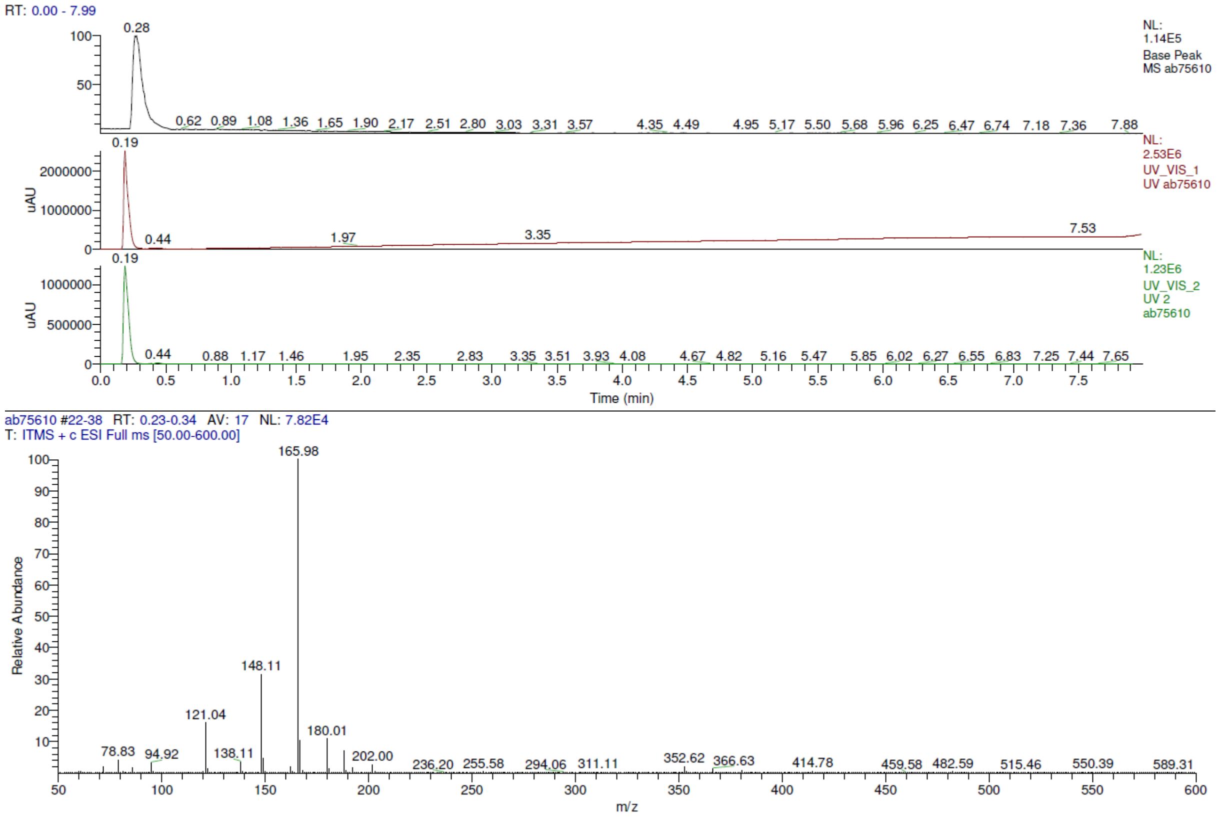

Figure 51 SI. ESI-MS spectrum of **S3** from the oil of the previous third fraction, now in reversed phase chromatography.

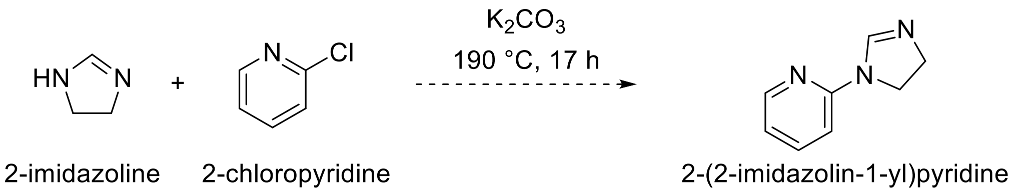

	Solvent	T [°C]	Reaction time [h]	Comment
**S4**	–	190	17	Analog to a literature procedure for a similar synthesis[38] Product signals after column chromatography (10 – 20 % MeOH in EtOAc, R_f = 0.18 for 10 % MeOH in EtOAc). In the mass spectrum mainly product, at 148.01 m/z (Figure 52). However, still two small signals at 120.99 m/z and 165.96 m/z, attributable to the above discussed impurities. This was also confirmed in the NMR, which, despite having assignable product signals, was not clean (Figure 53).

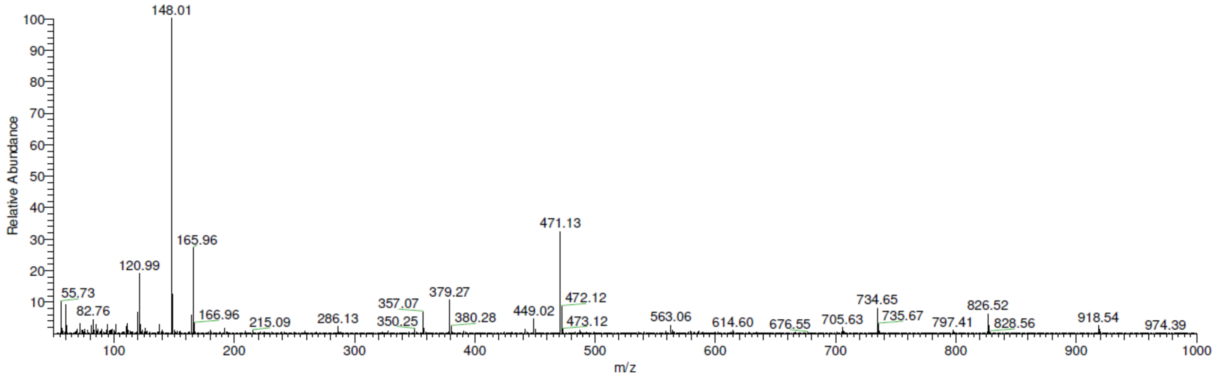

Figure 52 SI. ESI-MS spectrum of **S4** form the third fraction after column chromatography.

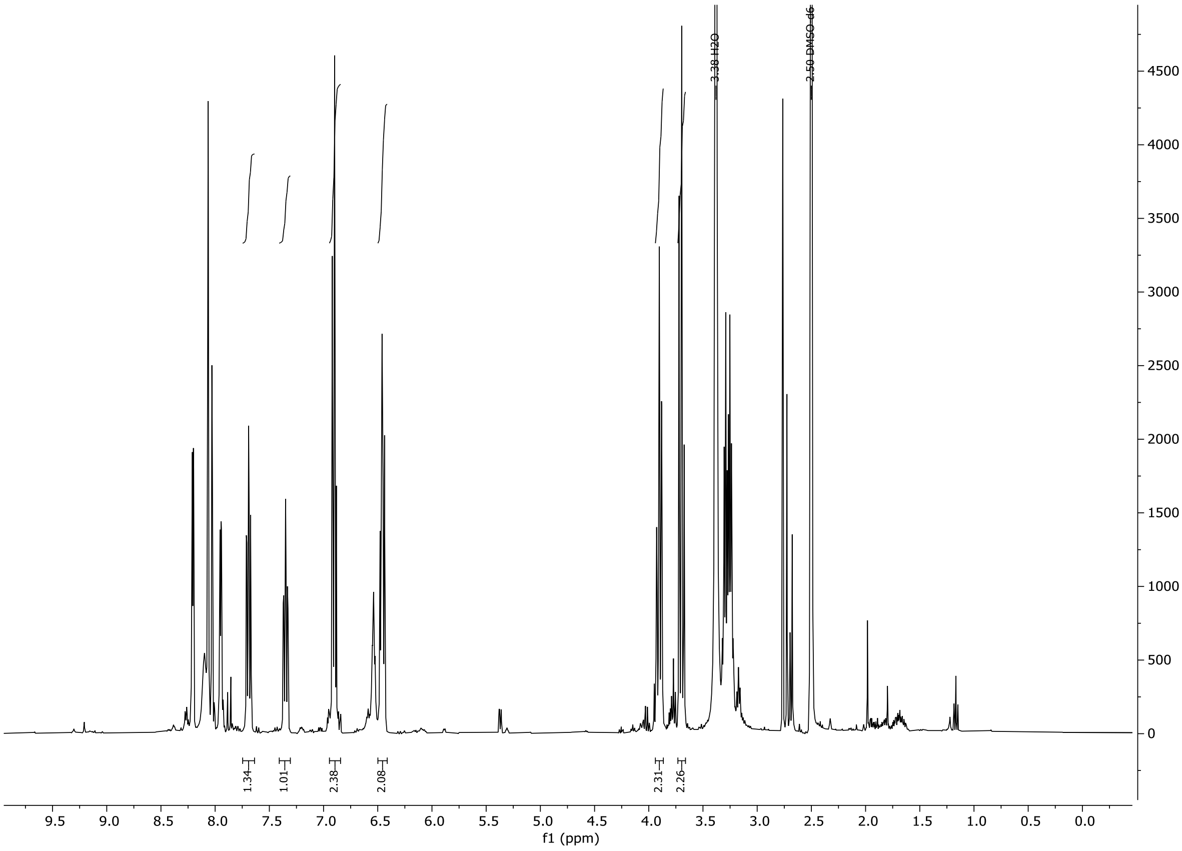

Figure 53 SI. ^{1}H NMR spectrum of **S4** in DMSO-d_6 from the dried third fraction of column chromatography.

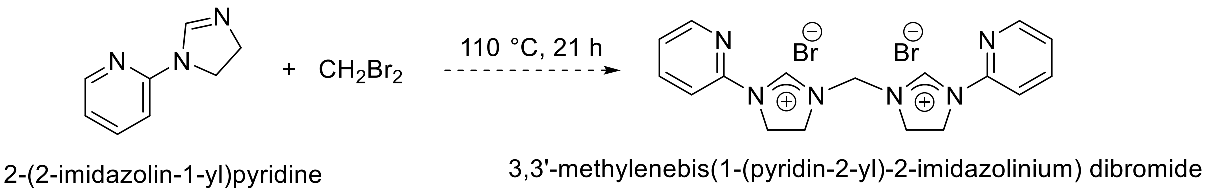

	Solvent	T [°C]	Reaction time [h]	Comment
**S5**	–	110	21	reaction of 2-(2-imidazolin-1-yl)pyridine (from **S2**) - despite only impure - with CH_2Br_2 only one potential product signal (Figure 54 SI): $[HL1]^+$ calcd., 307.17; found, 307.19 (30). Formylated side product and other degradation products are present.

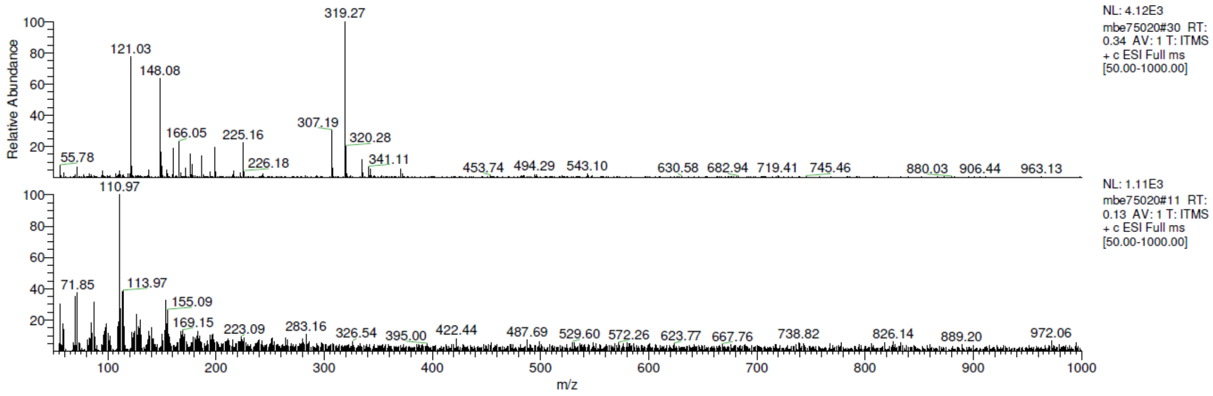

Figure 54 SI. ESI-MS spectrum of **S5** from the crude product.

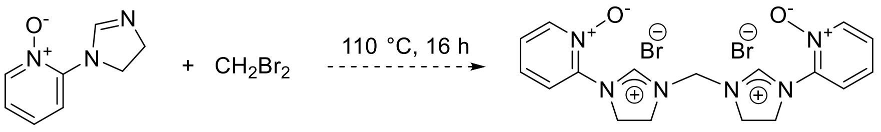

2-(2-imidazolin-1-yl)pyridine 1-oxide

2,2'-(methylenebis(2-imidazolinium-3,1-diyl))bis(pyridine 1-oxide) dibromide

	Solvent	T [°C]	Reaction time [h]	Comment
**S6**	–	110	16	No product formation observed (Figure 55 SI)

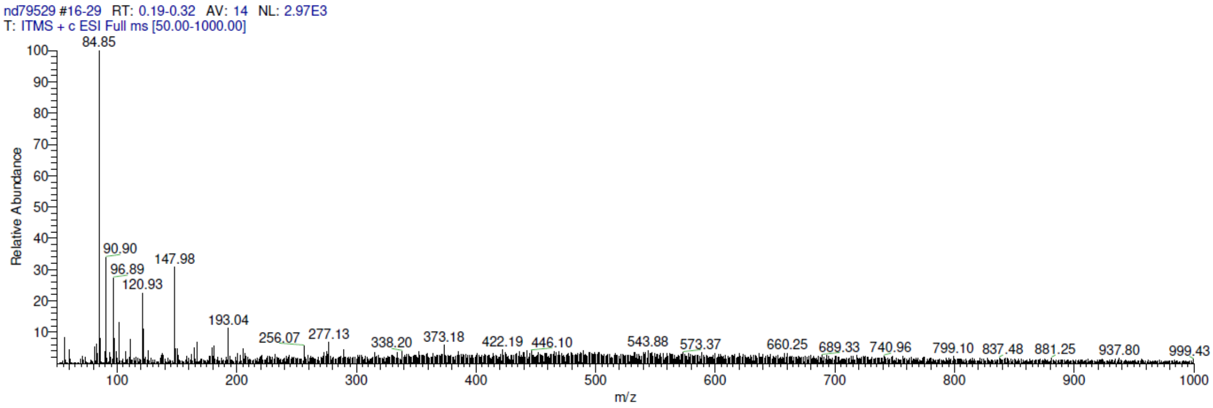

Figure 55 SI. ESI-MS spectrum of **S6** from the crude product.

11. References SI

[1] T. P. Schlachta, master's thesis, Technical University of Munich (Garching, Germany), **2020**.
[2] *APEX suite of crystallographic software*, APEX4, Bruker AXS Inc.: Madison, Wisconsin, USA, **2021**.
[3] *SAINT*, Version 8.38A, Bruker AXS Inc.: Madison, Wisconsin, USA, **2017**.
[4] *SADABS*, Version 2016/2, Bruker AXS Inc.: Madison, Wisconsin, USA, **2016**.
[5] G. M. Sheldrick, *Acta Crystallogr. Sect. C* **2015**, *71*, 3-8, DOI: 10.1107/S2053229614024218.
[6] G. M. Sheldrick, *Acta Crystallographica Section A* **2015**, *71*, 3-8, DOI: 10.1107/S2053273314026370.
[7] C. B. Hübschle, G. M. Sheldrick, B. Dittrich, *J. Appl. Crystallogr.* **2011**, *44*, 1281-1284, DOI: 10.1107/S0021889811043202.
[8] A. J. Wilson, *International Tables for Crystallography,* Kluwer Academic Publishers: Dordrecht, The Nederlands, **1992**.
[9] A. Spek, *Acta Crystallographica Section D* **2009**, *65*, 148-155, DOI: 10.1107/S090744490804362X.
[10] Jmol: an open-source Java viewer for chemical structures in 3D. http://www.jmol.org/
[11] T. P. Schlachta, G. G. Zámbó, M. J. Sauer, I. Rüter, C. A. Hoefer, S. Demeshko, F. Meyer, F. E. Kühn, *J. Catal.* **2023**, *426*, 234-246, DOI: 10.1016/j.jcat.2023.07.018.
[12] A. Poater, F. Ragone, S. Giudice, C. Costabile, R. Dorta, S. P. Nolan, L. Cavallo, *Organometallics* **2008**, *27*, 2679-2681, DOI: 10.1021/om8001119.
[13] A. Poater, F. Ragone, R. Mariz, R. Dorta, L. Cavallo, *Chem. Eur. J.* **2010**, *16*, 14348-14353, DOI: 10.1002/chem.201001938.
[14] L. Falivene, Z. Cao, A. Petta, L. Serra, A. Poater, R. Oliva, V. Scarano, L. Cavallo, *Nat. Chem.* **2019**, *11*, 872-879, DOI: 10.1038/s41557-019-0319-5.
[15] I. Kumpina, N. Brodyagin, J. A. MacKay, S. D. Kennedy, M. Katkevics, E. Rozners, *J. Org. Chem.* **2019**, *84*, 13276-13298, DOI: 10.1021/acs.joc.9b01133.
[16] J. P. Thenot, T. I. Ruo, O. J. Bouwsma, *Anal. Lett.* **1980**, *13*, 759-769, DOI: 10.1080/00032718008077997.
[17] B. Kaboudin, M. Khodamorady, *Synlett* **2010**, *2010*, 2905-2907, DOI: 10.1055/s-0030-1259029.
[18] M. M. Watts, *Journal of the American Oil Chemists' Society* **1990**, *67*, 993-995, DOI: 10.1007/BF02541864.
[19] B. G. Harnsberger, J. L. Riebsomer, *J. Heterocycl. Chem.* **1964**, *1*, 188-192, DOI: 10.1002/jhet.5570010408.
[20] C.-Y. Liao, K.-T. Chan, C.-Y. Tu, Y.-W. Chang, C.-H. Hu, H. M. Lee, *Chem. Eur. J.* **2009**, *15*, 405-417, DOI: 10.1002/chem.200801296.
[21] Y. Wang, L. Zhang, *Synthesis* **2015**, *47*, 289-305, DOI: 10.1055/s-0034-1379884.
[22] S. Youssif, *ARKIVOC* **2001**, *2001*, 242-268, DOI: 10.3998/ark.5550190.0002.116.
[23] O. Toma, N. Mercier, M. Allain, A. Forni, F. Meinardi, C. Botta, *Dalton Trans.* **2015**, *44*, 14589-14593, DOI: 10.1039/C5DT01801C.
[24] T. Riis-Johannessen, L. P. Harding, J. C. Jeffery, R. Moon, C. R. Rice, *Dalton Trans.* **2007**, 1577-1587, DOI: 10.1039/B700539C.
[25] S. Ouizem, D. Rosario-Amorin, D. A. Dickie, R. T. Paine, A. de Bettencourt-Dias, B. P. Hay, J. Podair, L. H. Delmau, *Dalton Trans.* **2014**, *43*, 8368-8386, DOI: 10.1039/C3DT53611D.
[26] D. Cartwright, J. R. Ferguson, T. Giannopoulos, G. Varvounis, B. J. Wakefield, *Tetrahedron* **1995**, *51*, 12791-12796, DOI: 10.1016/0040-4020(95)00734-P.
[27] S. J. Bullock, L. P. Harding, M. P. Moore, A. Mills, S. A. F. Piela, C. R. Rice, L. Towns-Andrews, M. Whitehead, *Dalton Trans.* **2013**, *42*, 5805-5811, DOI: 10.1039/C3DT00090G.

[28] U. Bildziukevich, L. Rárová, D. Šaman, L. Havlíček, P. Drašar, Z. Wimmer, *Steroids* **2013**, *78*, 1347-1352, DOI: 10.1016/j.steroids.2013.10.003.
[29] A. Bolje, J. Košmrlj, *Org. Lett.* **2013**, *15*, 5084-5087, DOI: 10.1021/ol4024584.
[30] J. P. Kutney, R. Greenhouse, *Synth. Commun.* **1975**, *5*, 119-124, DOI: 10.1080/00397917508061441.
[31] S. Ranganathan, Y. B. R. D. Rajesh, I. L. Karle, *Synlett* **2007**, *2007*, 1215-1218, DOI: 10.1055/s-2007-977445.
[32] Y. Wang, Y. Zhang, B. Yang, A. Zhang, Q. Yao, *Org. Biomol. Chem.* **2015**, *13*, 4101-4114, DOI: 10.1039/C5OB00045A.
[33] B. Alici, E. Çetinkaya, B. Çetinkaya, *Heterocycles* **1997**, *45*, 29-36, DOI: 10.3987/COM-96-7511.
[34] N. Aoyagi, Y. Furusho, Y. Sei, T. Endo, *Tetrahedron* **2013**, *69*, 5476-5480, DOI: 10.1016/j.tet.2013.04.110.
[35] G. R. Fulmer, A. J. M. Miller, N. H. Sherden, H. E. Gottlieb, A. Nudelman, B. M. Stoltz, J. E. Bercaw, K. I. Goldberg, *Organometallics* **2010**, *29*, 2176-2179, DOI: 10.1021/om100106e.
[36] M. Bessel, F. Rominger, B. F. Straub, *Synthesis* **2010**, *2010*, 1459-1466, DOI: 10.1055/s-0029-1218702.
[37] Y. Génisson, N. L.-d. Viguerie, C. André, M. Baltas, L. Gorrichon, *Tetrahedron: Asymmetry* **2005**, *16*, 1017-1023, DOI: 10.1016/j.tetasy.2005.01.014.
[38] A. Raba, M. R. Anneser, D. Jantke, M. Cokoja, W. A. Herrmann, F. E. Kühn, *Tetrahedron Lett.* **2013**, *54*, 3384-3387, DOI: 10.1016/j.tetlet.2013.04.060.
